# Inhibition of Chk1 Kills Tetraploid Tumor Cells through a p53-Dependent Pathway

**DOI:** 10.1371/journal.pone.0001337

**Published:** 2007-12-26

**Authors:** Ilio Vitale, Lorenzo Galluzzi, Sonia Vivet, Lisa Nanty, Philippe Dessen, Laura Senovilla, Ken A. Olaussen, Vladimir Lazar, Michelle Prudhomme, Roy M. Golsteyn, Maria Castedo, Guido Kroemer

**Affiliations:** 1 INSERM, U848, Cancer and Immunity, Villejuif, France; 2 Institut Gustave Roussy,Villejuif, France; 3 Université Paris Sud-11, Villejuif, France; 4 Centre National de la Recherche Scientifique (CNRS), FRE2939, Villejuif, France; 5 Unité de Génomique Fonctionnelle, Institut Gustave Roussy,Villejuif, France; 6 Université Blaise Pascal, Synthèse et Etude de Systèmes à Intérêt Biologique, UMR 6504 Centre National de la Recherche Scientifique (CNRS), Aubière, France; 7 Institut de Recherche Servier, Croissy sur Seine, France; Dresden University of Technology, Germany

## Abstract

Tetraploidy constitutes an adaptation to stress and an intermediate step between euploidy and aneuploidy in oncogenesis. Tetraploid cells are particularly resistant against genotoxic stress including radiotherapy and chemotherapy. Here, we designed a strategy to preferentially kill tetraploid tumor cells. Depletion of checkpoint kinase-1 (Chk1) by siRNAs, transfection with dominant-negative Chk1 mutants or pharmacological Chk1 inhibition killed tetraploid colon cancer cells yet had minor effects on their diploid counterparts. Chk1 inhibition abolished the spindle assembly checkpoint and caused premature and abnormal mitoses that led to p53 activation and cell death at a higher frequency in tetraploid than in diploid cells. Similarly, abolition of the spindle checkpoint by knockdown of Bub1, BubR1 or Mad2 induced p53-dependent apoptosis of tetraploid cells. Chk1 inhibition reversed the cisplatin resistance of tetraploid cells *in vitro* and *in vivo*, in xenografted human cancers. Chk1 inhibition activated p53-regulated transcripts including Puma/BBC3 in tetraploid but not in diploid tumor cells. Altogether, our results demonstrate that, in tetraploid tumor cells, the inhibition of Chk1 sequentially triggers aberrant mitosis, p53 activation and Puma/BBC3-dependent mitochondrial apoptosis.

## Introduction

Cancer results from the accumulation of genetic and epigenetic alterations in which genomic instability conditions the progressive deterioration towards an ever more aggressive phenotype. One of the mechanisms of genomic instability involves a transient phase of polyploidization (in most cases tetraploidization), which may result from endoreplication (DNA replication without mitosis), endomitosis (karyokinesis without cytokinesis) [1] or aberrant cell fusion [2], [3]. Tetraploid cells then can undergo asymmetric cell division and/or chromosome loss, leading to aneuploidization and chromosomal instability [4]–[6].

Under normal conditions, a variety of endogenous tumor suppressor gene products prevent the generation of tetraploid cells. For instance, p53 is activated immediately after illicit tetraploidization induced either by fusion [7], endoreplication, or endomitosis [8], and promote cell cycle arrest [9]–[12] and/or the activation of pro-apoptotic genes that cause the elimination of the tetraploid cell by programmed cell death [7], [8], [12], [13]. Inactivation of the p53 pathway is hence permissive for tetraploidization [6]. Similarly, the loss of the tumor suppressor proteins p21 [14], Bax [8], APC [15], or Lats2 [12] facilitates the generation of tetraploid cells. Symmetrically, overexpression of oncogene products such as Aurora kinase A [16] or papillomavirus E6 [17] induces tetraploidization. One possible interpretation of these findings is that a hypothetical “tetraploidy checkpoint” [9] would prevent the generation or propagation of tetraploid cells, by mean of a stable cell cycle arrest or their elimination by apoptosis. Indeed, defective checkpoints have been involved in oncogenesis, at several levels. As an example, it has been shown that the activation of Chk2 helps in the elimination of potentially malignant cells [18], [19], and that familial loss-of-function mutations [20] or an acquired interruption of Chk2 activation [18], [19] contribute to oncogenesis. The checkpoint kinase-1 (Chk1) is also lost in aggressive lymphoid tumors [20]. Nonetheless, Chk1 is involved not only in tumor suppression, and its inhibition can sensitize tumor cells to DNA damage [21]–[24], presumably because the failure to arrest the cell cycle upon DNA damage is a lethal event.

Experimental tetraploidization of p53-negative mammary epithelial cells can be employed as a method to generate transformed, tumorigenic cells, thus providing a proof-of-principle that tetraploidization may constitute an important intermediate step in carcinogenesis [5]. Heterotypic fusion has been described *in vivo*, in rodent models, for instance between tumor cells and infiltrating cells from myeloid origin [25] and may contribute to the plasticity of cancer. A fusogenic retrovirus can induce the transformation of human cells *in vitro* through cell fusion [26]. Accordingly, pre-malignant and malignant tetraploid cells have been documented in precancerous lesions such as Barret's esophagus [27], in pre-invasive lesions of the uterine cervix [17], [28], in laryngeal dysplasia [29], and in chronic ulcerative colitis dysplasia [30]. The presence of sub-clones of tetraploid/octoploid cells in human tumors has been correlated with worse prognosis, for instance in uterine cervix carcinoma [31], squamous cell carcinoma of the head and neck [32], and in poorly differentiated prostate carcinoma [33].

Tetraploid cells are intrinsically resistant against genotoxic stress mediated by ionizing irradiation or by genotoxic agents used for anti-cancer chemotherapy, including platinum compounds (such as cisplatin and oxaliplatin) and topoisomerase inhibitors (such as camptothecin) [8], [25], meaning that tetraploid cells have a high chance to survive apoptosis-inducing regimes. Since polyploid tumor cells accumulate in particular areas of the cancerous lesion, for instance in areas of hypoxia [34], it can be speculated that tetraploid cells might contribute to chemotherapeutic failure.

Based on these considerations, we wondered whether it might be possible to design strategies for the destruction of tetraploid tumor cells. Here, we report that inhibition of one particular drugable kinase, Chk1, leads to the selective destruction of tetraploid cancer cells. In addition, we provide an exhaustive characterization of the pro-apoptotic signal transduction pathway elicited by Chk1 inhibition.

## Results

### Aberrant mitoses of tetraploid cells with an intact spindle assembly checkpoint (SAC)

**Figure 1:**
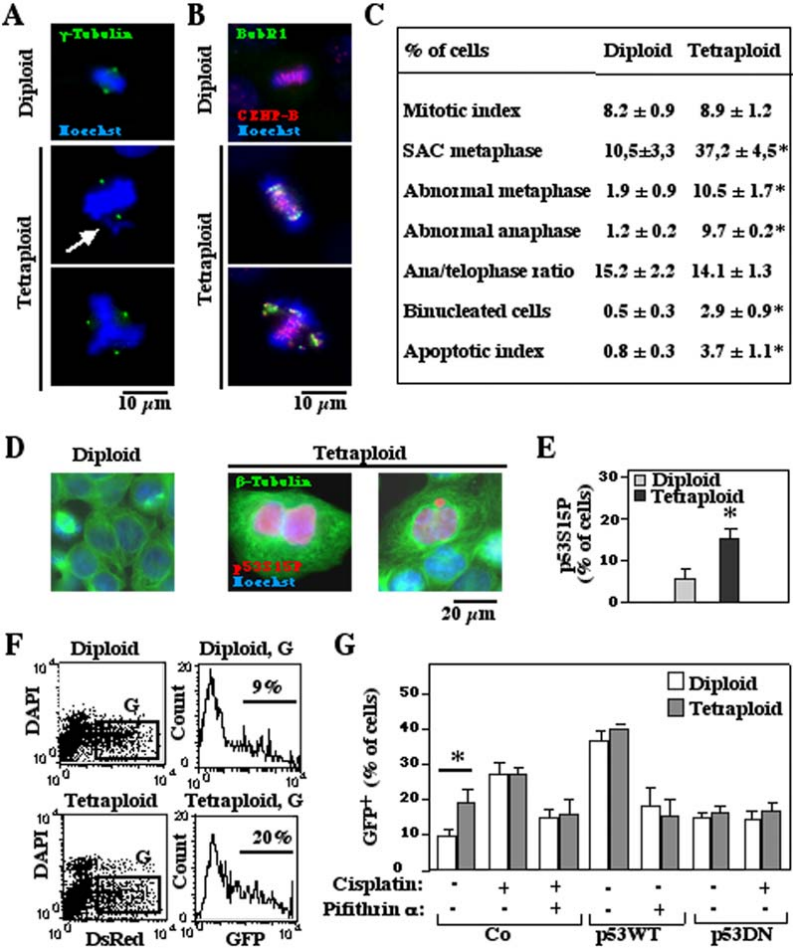
Abnormal mitoses linked to p53 activation in tetraploid HCT116 cells. A. Abnormal mitoses. Tetraploid cells were stained to visualize chromosomes (Hoechst 33342, blue) and γ-tubulin (green). The arrow marks a misaligned chromosome. B. Activation of the spindle assembly checkpoint (SAC) in tetraploid mitoses. Cells are stained to visualize chromosomes (blue), centromeres (CENP-B, red) and the SAC protein BubR1 (green). The white color results from the overlap of the three fluorescence signals, indicating recruitment of BubR1 to centromeres. C. Quantitation of the data obtained in A and B, comparing diploid and tetraploid cells in three independent experiments (X±SEM). D, E. p53 phosphorylation linked to abnormal mitoses. Representative examples of tetraploid cells that show incomplete cytokinesis, binucleation and micronucleation coupled to p53 phosphorylation on serine 15 (detected by immunofluoresence staining) are shown in D and quantified in E. F, G. Evidence for transcriptional activation of p53 in tetraploid cells. Diploid or tetraploid cells were transfected with dsRed (red fluorescence), a p53-inducible GFP construct (green fluorescence), and either empty vector only, a plasmid encoding for wild type p53 or dominant-negative p53 (H175) and then cultured for 48 h in the absence or presence of the p53 inhibitor cyclic pifithrin-α. Cells were labeled with the vital stain DAPI and the frequency of transfected (dsRed-expressing) cells that express GFP was determined by cytofluorometry as shown in D for vector-only controls cultured in the absence of pifithrin. Representative results (X±SEM, n = 3) from three independent experiments are shown in E. Asterisks indicate significant (p<0.01) differences between diploid and tetraploid cells.

Recently, we have developed a panel of tetraploid HCT116 and RKO cell clones that bear exactly twice the normal chromosome content than their diploid precursors, yet lack any other discernible numeric or structural chromosomic aberration [8] [35]. Tetraploid tumor cells exhibit a slightly reduced growth rate, by about 10%, as compared to their diploid precursors [8], a finding that prompted us to investigate the rate and efficacy of mitoses. While there was no difference in the rate of mitotic events between diploid and tetraploid HCT116 cells, we found a significantly increased frequency of abnormal mitoses in tetraploid cells (**Fig. 1**). Such abnormal mitoses were characterized by misaligned chromosomes during metaphase, multipolar (mostly tri- or tetrapolar) metaphases, anaphase bridges and cytokinesis failure resulting into binucleation (**Fig. 1A,C**). Among tetraploid, apparently normal or aberrant metaphases were frequently characterized by the activation of the spindle assembly checkpoint (SAC), as indicated by the presence of BubR1 on kinetochores (**Fig. 1B,C**). Accordingly, SAC was intact in tetraploid cells, because, treatment with nocodazole or docetaxel induced similar percentages of mitotic arrested cells and cell death in tetraploid and diploid clones (**Fig. S1**). Videomicroscopy of the nuclear and cellular divisions of tetraploid cells transfected with a histone H2B-GFP fusion construct (which allows to visualize chromosomes in live cells) confirmed that 10 to 15% of tetraploid mitoses were aberrant (**Videos S1, S2**), while less than 3% of diploid mitoses were abnormal.

A fraction of tetraploid cells displayed an activating phosphorylation of p53 (detectable by immunofluorescence using an antibody that stains p53 phosphorylated on serine 15) within their nuclei, and these phospho-p53-positive cells were in most cases (approximately 80%) bi- or multi-nucleated and/or or contained micronuclei (**Fig. 1D,E**), suggesting that p53 activation resulted from mitotic failure. The large majority of mitoses (as discerned by the presence of mitotic figures after Hoechst 33342 staining) were phospho-p53-negative. Only in a small percentage of mitoses that bears features of imminent apoptosis (with clumpy chromatin condensation), p53 appears to be phosphorylated (not shown). In contrast normal interphases (G1, S or G2) were mostly (>99%) negative for phospho-p53. Only cells with abnormal nuclear morphologies manifested the p53 phosphorylation on Ser15.

The frequency of cells that activated a p53-inducible green fluorescent protein (GFP) construct was higher among tetraploid than among diploid cells (**Fig. 1F,G**). As an internal control that the p53-inducible GFP indeed detected an elevated p53-mediated transactivation, we found that inhibition of p53 either chemically (with cyclic pifithrin-α) or genetically (by transfection with a dominant-negative p53 mutant) inhibited the production of p53-inducible GFP in response to cisplatin down to the background level (**Fig. 1G**). Altogether, these results suggest that catastrophic mitoses are responsible for an enhanced activation of the p53 system in tetraploid cells.

### Aberrant mitoses of tetraploid cells are exacerbated by Chk1 inhibition

**Figure 2:**
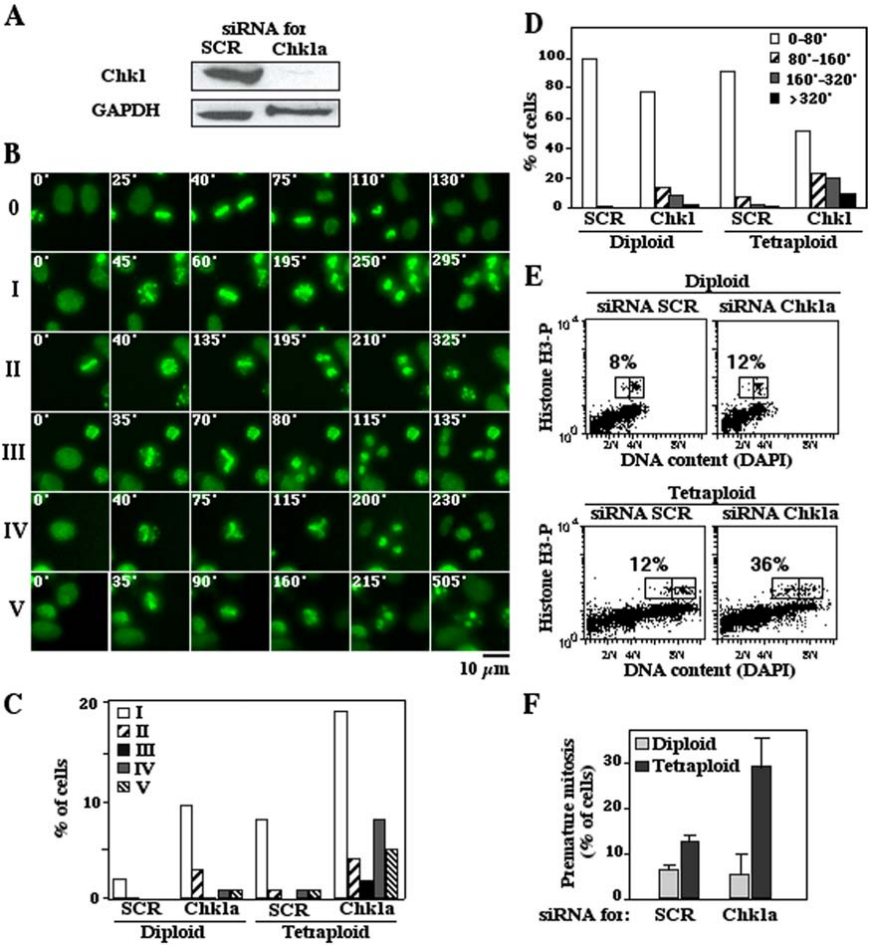
Effect of Chk1 depletion on mitosis and p53 activation in tetraploid cells. A. Efficient Chk1 depletion after transfection with a specific siRNA. Tetraploid RKO cells were transfected with a Chk1-specific siRNA (Chk1a) or scrambled (SCR) control siRNA and the abundance of Chk1 was determined by immunobloting. GAPDH was detected to control equal loading. B–D. Videomicroscopic analyses of tetraploid cell division. Tetraploid RKO cells stably transfected with a histone H2B-GFP fusion construct (green fluorescence marking chromosomes) were transfected with a Chk1-specific siRNA (Chk1a) and monitored 48 h later for abnormal mitosis. Representative sequences of pictures in B illustrate normal mitosis (0), delayed mitotic exit resulting in apparently normal division (I), apoptotic disintegration of daughter cells after cytokinesis (II), abnormal metaphase plates leading to formation of binucleated cells (III), multipolar mitoses (IV) and apoptosis during or shortly after the metaphase (V). The frequency of mitotic aberrations observed in cells that were transfected with a control siRNA (SCR) or the Chk1-depleting siRNA was calculated after having monitored 150 to 200 mitoses (C) and the length of mitosis was computed (D). E, F. Premature mitosis in Chk1-depleted tetraploid cells. Thirty-six hours after transfection with control siRNA (SCR) or Chk1-depleting siRNA, diploid or tetraploid cells were stained to measure DNA content (with DAPI) and histone H3 phosphorylation, followed by cytofluorometric analysis. The rectangle in E marks the population of cells showing the phosphorylation of mitotic histone H3. Numbers refer to percentage of diploid or tetraploid cells with less than 4N or 8N, respectively, that manifest premature mitosis. The quantification is represented in F. (X±SEM, n = 3).

In view of the apparent difficulty of tetraploid cells to successfully complete mitosis, we wondered whether these cells might rely more heavily on the cell cycle checkpoint kinase Chk1 than diploid cells. Transient transfection with a Chk1-specific small interfering RNA (siRNA) (**Fig. 2A**) caused a dramatic increase in aberrant mitoses of tetraploid cells (one third of all mitoses), resulting in delayed mitotic exit, apoptotic disintegration of both daughter cells after cytokinesis, formation of binucleated cells, multipolar mitoses, or apoptosis during or shortly after the metaphase (**Fig. 2B,C,D** **and videos S3, S4, S5, S6, S7, S8**). This correlated with premature mitoses, detectable as events in which the phosphorylation of histone H3 by mitotic kinases occurs in cells that have not duplicated their genome, and hence have less than 8N DNA content (**Fig. 2E,F**). The percentage of premature mitoses was not affected by preincubation with the pancaspase inhibitor Z-VAD.fmk (not shown). The increase in frequency of aberrant or premature mitoses induced by Chk1 inhibition was more pronounced among tetraploid than among diploid cells (**Fig. 2A–F**).

### Chk1 inhibition abolished the spindle assembly checkpoint (SAC) and activates p53

**Figure 3:**
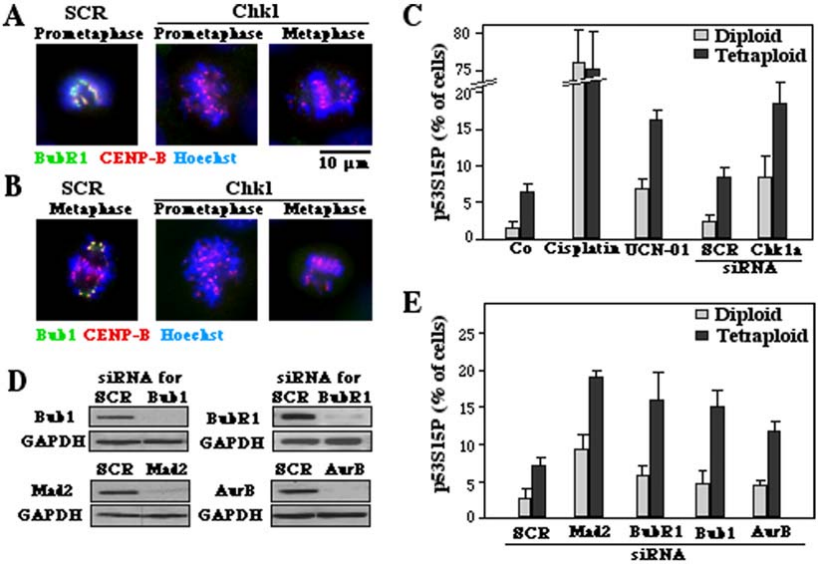
Phosphorylation of p53 on serine 15 in response to SAC inhibition. A, B. Abolition of SAC by Chk1 depletion. Tetraploid cells were either transfected with a scrambled control siRNA (SCR) or with a Chk1-specific siRNA and then subjected 48 h later to immunofluorescence to determine the centromeric location of BubR1 or Bub1 (as in Fig. 1B) as a sign of SAC activation. Note that depletion of Chk1 fully abolished SAC. C. p53 activation by Chk1 inhibition in diploid versus tetraploid cells. Cells treated with 15 µM cisplatin, a Chk1-depleting siRNA (or its control SCR) or the Chk1 inhibitor UCN-01 were stained 48 h later to detect p53 phosphorylation on serine 15. Cisplatin treatment was used as an internal positive control. D. Efficacy of siRNAs directed against SAC proteins, as determined by immunoblot, 48 hours after transfection. E. p53 phosphorylation on serine 15 after depletion of SAC proteins. (X±SEM, n = 3).

The knockdown of Chk1 resulted in a complete abolition of SAC resulting in the failure to recruit BubR1 and Bub1 to kinetochores during the prometaphase and aberrant metaphases for diploid and tetraploid cells (**Fig. 3A,B** for tetraploid cells). Indeed, after Chk1 knockdown less than 30% of prometaphases and aberrant metaphases exhibited the localization of Bub or BubR1 to kinetochores, indicating abolition of SAC in more than 70% of the cells. The percentage of SAC inhibition, as detectable among aberrant metaphases, was similar in diploid and tetraploid cells (although the number of aberrant metaphases is much higher in tetraploid cells, see Fig. 1). Small interfering RNA (siRNA)-mediated depletion of Chk1 or its pharmacological inhibition by UCN-01 (7-hydroxystaurosporine) also resulted in the activating phosphorylation of p53, more frequently in tetraploid than in diploid cells (**Fig. 3C**). Similarly, the knockdown of the essential SAC proteins Bub1, BubR1, Mad2 or Aurora B induced the phosphorylation of p53 on serine 15, more so in tetraploid than in diploid cells (**Fig. 3D,E**). Taken together, these data indicate that Chk1 inhibition abolishes SAC, and that SAC inhibition exacerbates mitotic defects coupled to p53 activation in tetraploid tumor cells.

### Chk1 inhibition kills tetraploid cells

**Figure 4:**
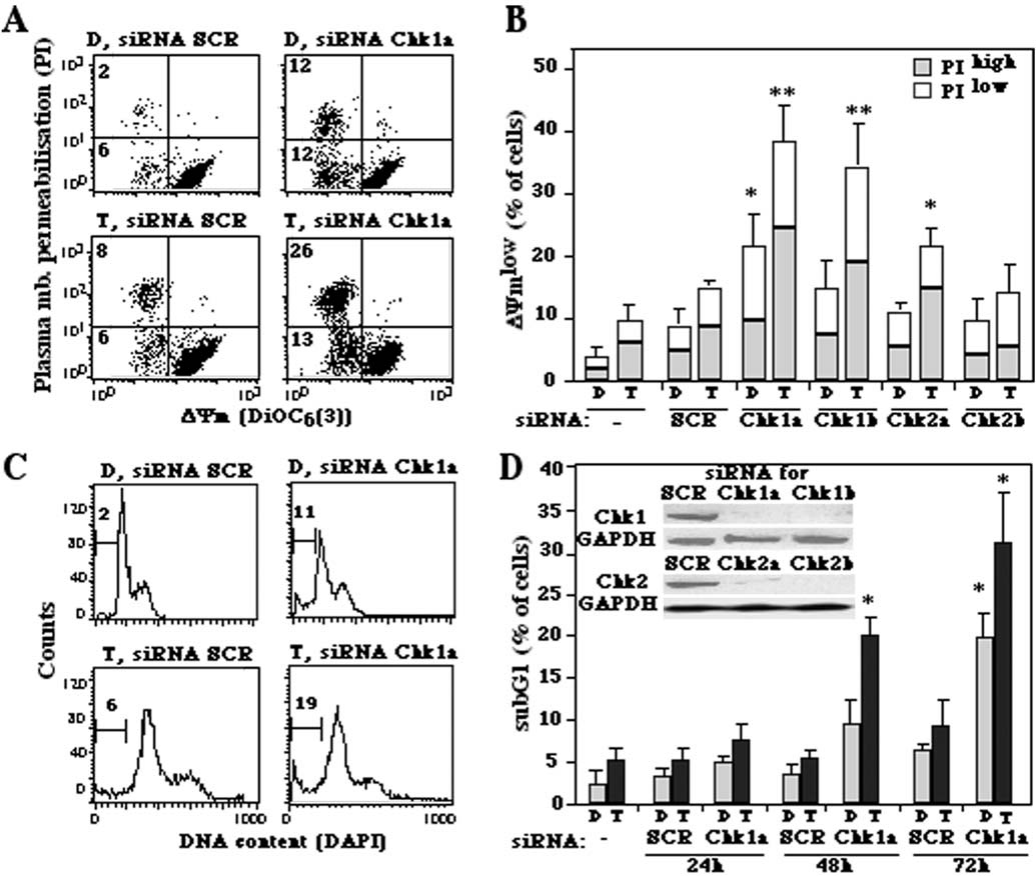
Apoptosis induction by depletion of Chk1 in tetraploid HCT116 cells. A, B. Loss of the mitochondrial transmembrane potential (ΔΨ_m_) and viability induced by Chk1 depletion. Diploid (D) or tetraploid (T) cells were transfected with either of two siRNAs specific for Chk1 (Chk1a, Chk1b) or Chk2 (Chk2a, Chk2b) and stained 48 h later to measure ΔΨ_m_ (with DiOC_6_(3)) and viability (with PI). Representative FACS pictograms are shown in A and data are quantified in B. C, D. Apoptotic DNA loss induced by Chk1 depletion. Cells were stained with DAPI to measure DNA content, 24, 48 or 72 h after transfection with Chk1-depleting siRNA. Data shown in C have been obtained 48 h after transfection. The numbers in C refer to the percentage of cells with a sub-G1 DNA content. Inserts in D demonstrate siRNA-mediated Chk1 and Chk2 depletion, as detected by immunoblots. * p<0.01 and ** p<0.001 as compared to untreated or control siRNA-transfected (SCR) controls. Data represent the mean of three independent experiments in triplicate. (X± SEM).

In view of the mitotic or post-mitotic apoptosis induced by Chk1 depletion (Fig. 2A–C), we quantified the frequency of cell death induced by Chk1 inhibition by cytofluorometric methods. Simultaneous detection of dying cells (which exhibit a dissipated mitochondrial transmembrane potential, i.e. ΔΨ_m_, and hence a reduced DiOC_6_(3) incorporation) and dead cells (which possess permeabilized plasma membranes and hence incorporate the vital dye propidium iodide, i.e. PI) revealed that depletion of Chk1 with either of two distinct siRNAs killed tetraploid HCT116 cells more efficiently than diploid cells. This effect was less pronounced when instead of Chk1, Chk2 was depleted (**Fig. 4A,B**). The downregulation of Chk1 was more efficient in inducing an apoptosis-associated DNA loss (sub-G1 DNA content, as determined by staining with DAPI) among tetraploid than among diploid cells (**Fig. 4C,D**). Similar results were obtained for another colon carcinoma cell line, namely RKO (**Fig. S2 A**) in thus far that Chk1 depletion was more efficient in killing tetraploid than diploid tumor cells.

**Figure 5:**
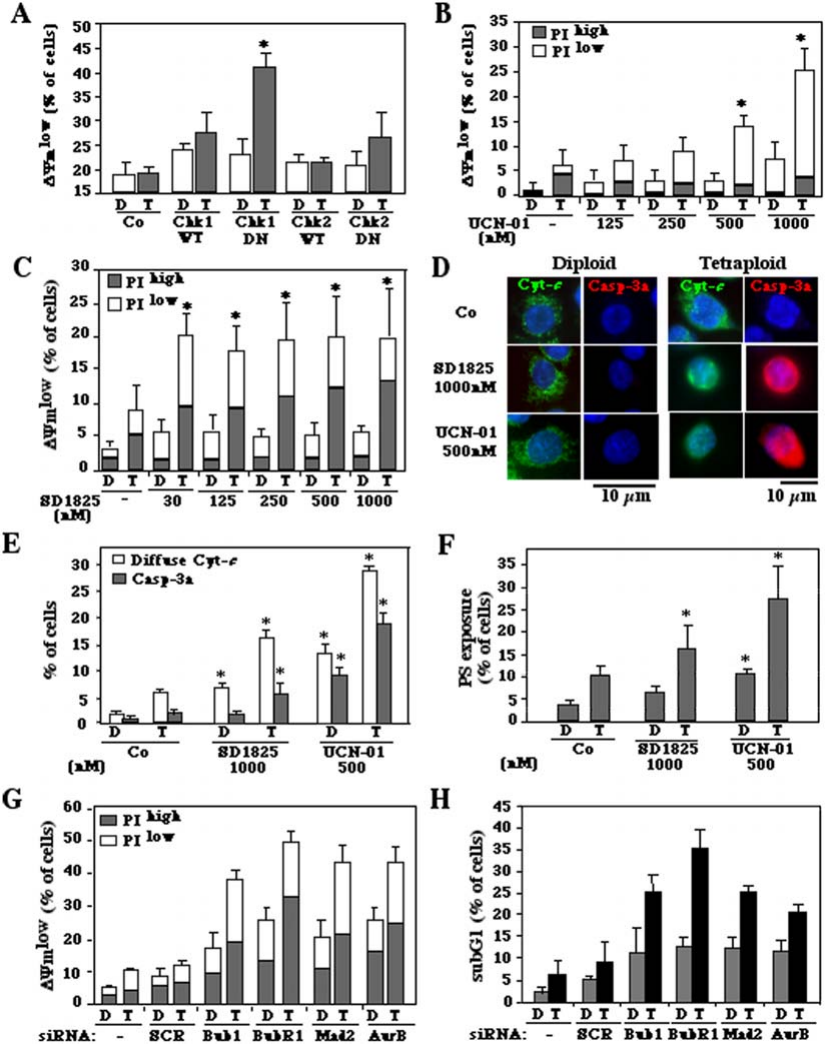
Apoptosis induction by Chk1 or SAC inhibition. A. Chk1 inhibition by a dominant-negative (DN) Chk1 mutant. Diploid or tetraploid HCT116 cells were transfected with vector only (Co) or vectors encoding wild type (WT) or DN Chk1 or Chk2, together with a DsRed-encoding construct. 48 h later, cells were stained with DiOC_6_(3) and the frequency of DiOC_6_(3)^low^ cells was determined among the transfected (dsRed^+^) population. B–F. Chk1 inhibition by pharmacological agents. HCT116 cells were cultured for 48 h in the presence of the indicated concentrations of UCN-01 (B,D) or SD1825 (C,D) and the frequency of dead and dying cells was measured by co-staining with DiOC_6_(3) and PI (as in Fig. 3A). Alternatively, cells cultured on polylysin slides were stained for the simultaneous immunofluorescence detection of mitochondrial cytochrome *c* (Cyt *c*) release and caspase-3 activation (Casp-3a). Representative microfluorographs are shown in D and the frequency of cells showing diffuse Cyt *c* staining and caspase-3 activation are scored in E. Finally, phosphatidylserine exposure was measured in diploid and tetraploid RKO cells by annexin V-FITC staining (F). Asterisks denote significant (p<0.01) effects of Chk1 inhibition. G, H. Preferential mortality of tetraploid cells subjected to SAC inhibition. Diploid or tetraploid cells were depleted from the indicated SAC proteins. Forty-eight hours later, apoptotic events were scored by measuring the frequency of ΔΨ_m_ (with DiOC_6_(3)) and viability (with PI) (G) or sub-G1 cells (H). Columns represent the average of three independent experiments ±SEM.

Since we worried about possible off-target effects of the Chk1-specific siRNAs, we repeated the experiments using alternative methods of Chk1 inhibition. Transfection with a dominant-negative (DN) Chk1 mutant (**Fig. 5A**) or inhibition of Chk1 with UCN-01 or SD1825 (**Fig. 5B–F**) resulted in an enhanced mortality of tetraploid HCT116 cells, with little or no effects on diploid cell. Efficient cell death of tetraploid cells was confirmed in RKO cells after pharmacological inhibition of Chk1 (**Fig. S2 B**). Moreover, alternative strategies to subvert SAC, by knockdown of Bub1, BubR1, Mad2 or Aurora B caused cell death much more efficiently in tetraploid than in diploid cancer cells (**Fig. 5G, H**).

Cell death induced by Chk1 inhibition was accompanied by the hallmarks of apoptosis such as nuclear DNA condensation (Fig. 2B), mitochondrial cytochrome *c* release, caspase-3 activation (Fig. 5D,E), and phosphatidylserine exposure (Fig. 5F). In conclusion, tetraploid cells die from apoptosis after catastrophic mitoses due to SAC inhibition.

### Synergistic killing of tetraploid cells by cisplatin plus Chk1 inhibition

**Figure 6:**
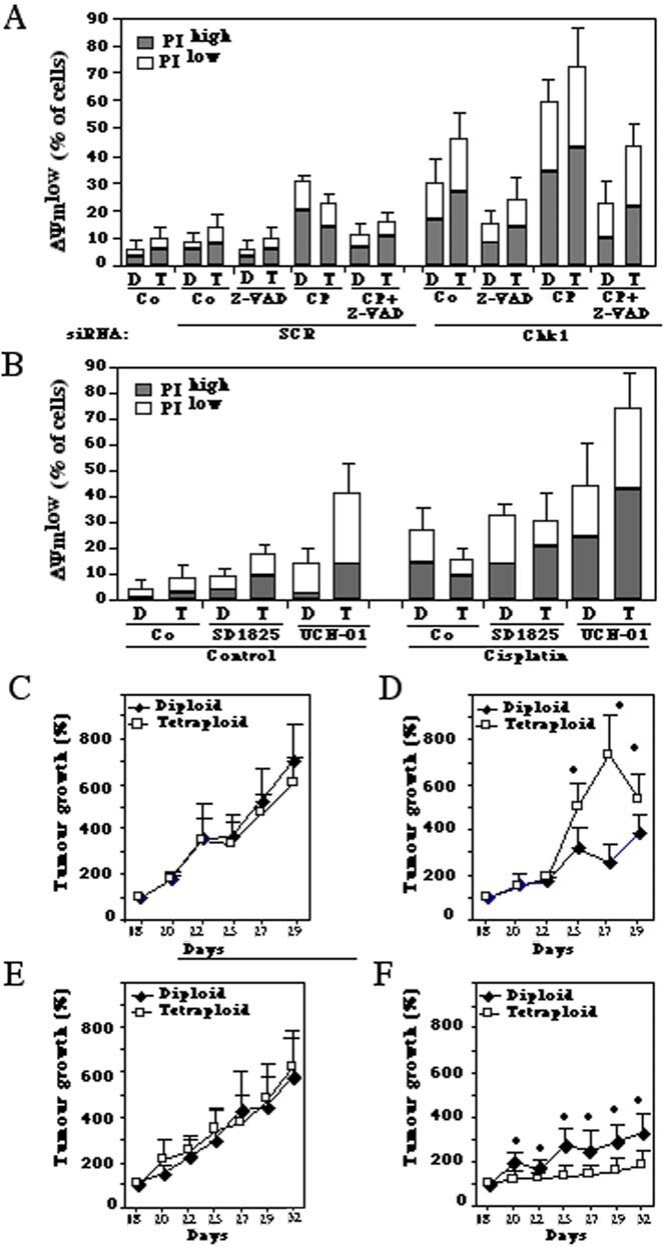
Combined effects of Chk1 inhibition and cisplatin on tetraploid tumor cells *in vitro* and *in vivo.* A. Cytotoxic effects of Chk1 depletion in combination with cisplatin. Diploid (D) and tetraploid (T) HCT116 cells were knocked down for Chk1 (or transfected with control siRNA SCR) for 24 h and then cultured in absence or presence of cisplatin (20 µM) for further 48 h. Finally, the frequency of dying (DiOC_6_(3)^low^ PI^−^) or dead (DiOC_6_(3)^low^ PI^+^) cells was monitored by DiOC_6_(3)/PI staining. B. Cell killing by pharmacological inhibition of Chk1 plus cisplatin. Cells were cultured with cisplatin (20 µM), UCN-01 (500 nM) and/or SD1825 (500 nM) for 48 h, and dead and dying cells were determined as in A. C–E. Combined effects of Chk1 inhibition and cisplatin on tetraploid tumors established *in vivo*. Diploid or tetraploid HCT116 tumors were established *in vivo* and their growth was monitored continuously from day 18 (when tumors measured 125 to 250 mm^3^), when animals were injected with PBS alone (controls in C), cisplatin (D), UCN-01 (E), or with a combination of both (F). Asterisks indicate significant differences between diploid and tetraploid cells (p<0.05, unpaired Student t test). The results shown in Fig. 6C–E are representative for three different experiments.

**Figure 7:**
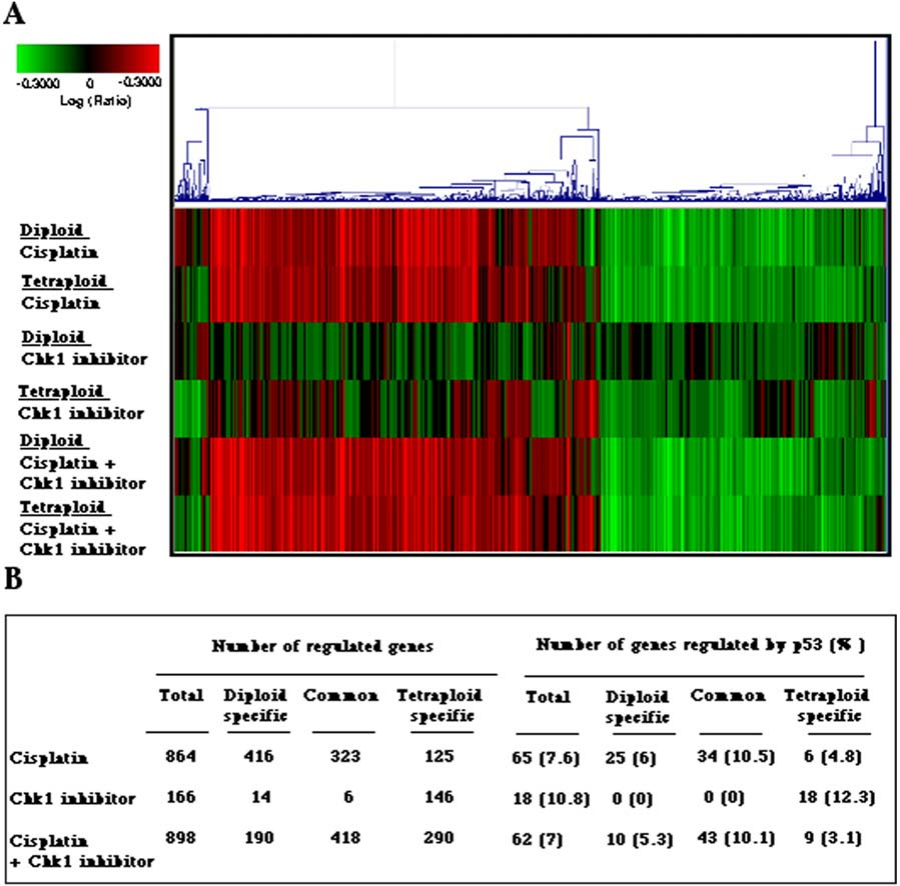
Microarray analyses of the transcriptome of diploid (D) and tetraploid (T) HCT116 cells treated with cisplatin, Chk1 inhibitor or the combination of both. A. Hierarchical cluster analysis of 1411 gene expression profiles in the 6 experimental samples is shown. Each row represents the combination of two dye-swap experiments compared to untreated control cells and each column represents a single gene. Red and green colors indicate an increase and a decrease, respectively, in the expression of genes as compared to unstimulated control cells. B. Overview of the modification of gene expression by cisplatin and Chk1 inhibition in diploid and tetraploid cells (p value<10^−5^).

As compared to their diploid counterparts, tetraploid cancer cells are relatively resistant against DNA damaging agents including cisplatin, both *in vitro* (Ref. [8], **Fig. 6A,B** for HCT116 and **Fig. S2 C** for RKO) and *in vivo* (**Fig. 6C,D**). Although there was no difference in the growth tumors originating from diploid versus tetraploid HCT116 cells inoculated into immunodeficient mice (Fig. 6C), tetraploid tumors responded far less to chemotherapy with cisplatin than diploid cancers (Fig. 6D). Chk1 depletion (Fig. 6A, Fig. S2 C) or inhibition (Fig. 6B) had an additive cytotoxic effect on tetraploid cells *in vitro*, in short-term assays. Treatment of tetraploid HCT116 tumors that had been established in xenotransplanted immunodeficient mice with a subtoxic dose of UCN-01 had no growth-inhibitory effect (**Fig. 6E**). However, the combination of cisplatin plus UCN-01 had a synergistic anti-cancer effect, which was particularly pronounced for tetraploid tumors (**Fig. 6F**). Based on these observations, we decided to explore the effect of cisplatin, Chk1 inhibition and the combination of both on the transcriptome of diploid and tetraploid colon cancer (HCT116) cells. The number of cisplatin-modulated genes showing a statistically relevant altered expression (p value<10^−5^) was higher among diploid than among tetraploid cells (**Fig. 7A,B**), correlating with the higher susceptibility of diploid cells to cisplatin-induced killing (Fig. 6D). In contrast, the number of genes modulated by Chk1 inhibition was significantly higher among tetraploid (152 genes) than among diploid cells (20 genes), with only six genes that were modulated in both diploid and tetraploid cells (Fig. 7A,B), again correlating with the enhanced killing of tetraploid cells by Chk1 inhibitors. The combination of cisplatin and Chk1 inhibition modified a large pool of transcripts that were common to tetraploid and diploid cells, with a higher number of tetraploid- than diploid- specific transcripts (Fig. 7A,B), in correlation with the particularly dramatic effects of the combined therapeutic regimen on tetraploid cells *in vivo* (Fig. 6F).

### Mechanisms of the cytotoxic effects of Chk1 inhibition

**Figure 8:**
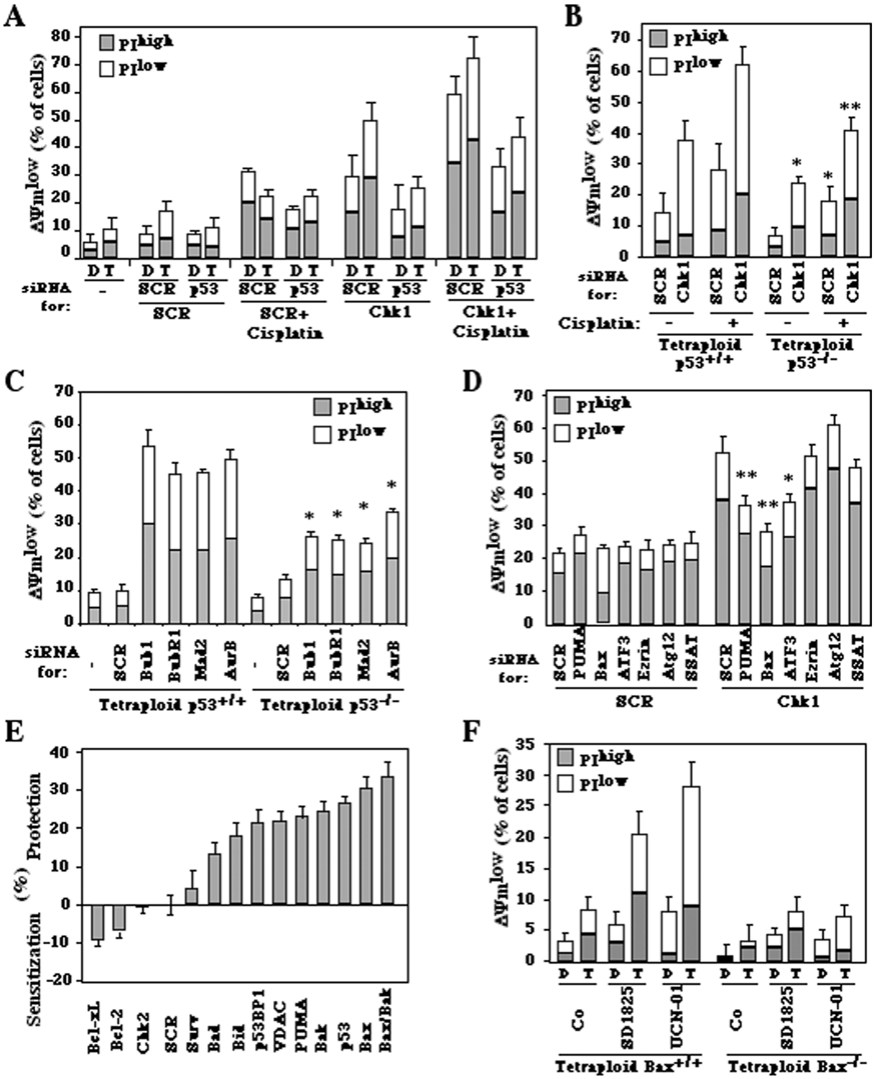
Involvement of p53 and Bcl-2 family proteins in apoptosis induction by Chk1 inhibition. A. Effect of p53 knockdown. Diploid or tetraploid HCT116 cells were transfected with control siRNA (SCR), a p53-specific siRNA, and/or Chk1-depleting siRNA and cultured in the presence of absence of cisplatin during the last 24 hours of the 72-hour experiment. Then, cells were stained with DiOC_6_(3)/PI. B,C. Effect of p53 knockout. Tetraploid HCT116 cells generated on a wild type (WT) or p53 knockout (p53^−/−^) background were treated with the indicated combination of Chk1-depleting siRNA and/or cisplatin (B) or were depleted from SAC proteins (C), followed by determination the frequency of dying (DiOC_6_(3)^low^ PI^−^) or dead (DiOC_6_(3)^low^ PI^+^). Results (X±SEM, n = 3) are representative of three independent determinations. Asterisks indicate a significant protection by p53 deletion. D. Contribution of p53 target genes to Chk1 depletion-induced apoptosis. Tetraploid HCT116 cells were transfected with the indicated siRNAs and the frequency of cell death was determined by DiOC_6_(3)/PI staining. E. Contribution of p53 and pro-apoptotic proteins of Bcl-2 family to the death of HCT116 cells induced by Chk1 depletion. Tetraploid HCT116 cells were subjected to the siRNA-mediated downregulation of Chk1 alone or together with the indicated gene products, followed by measurement of cell viability with a tetrazolium reduction assay. Negative values indicate sensitization to the cytotoxicity of Chk1 depletion while positive values indicate protective effects (X±SEM, n = 3). F. Effect of Bax knockout. Tetraploid HCT116 cells generated on a wild type (WT) or Bax knockout (Bax^−/−^) background were subjected to Chk1 inhibition, followed by determination of the frequency of dead and dying cells 48 h later. Results (X±SEM) are representative of three independent determinations.

Close inspection of the microarray data led us to the discovery that Chk1 inhibition caused the modulation of a particularly elevated percentage (12.3%) of p53 target genes in tetraploid cells (18 p53 target genes among a total of 146 Chk1-modulated genes) but not in diploid cells (none among 14 genes). This percentage is higher than that observed among the set of genes modulated by cisplatin (7.6%) or cisplatin plus Chk1 inhibitor (7.0%) in both diploid and tetraploid cells (Fig. 7B, **Tables S1**). These results corroborate the observation that Chk1 inhibition causes the activating phosphorylation of p53 (Fig. 3C). To clarify whether the transcription of p53 target genes is an epiphenomenon or if it is directly responsible for cell killing by Chk1 inhibition, we depleted p53 with a specific siRNA. p53 knockdown strongly reduced the cytotoxic effect of Chk1 depletion (**Fig. 8A**). Similarly, tetraploid derivatives of p53^−/−^ HCT116 cells, generated as previously described [8], died significantly less than the p53 proficient tetraploid cells in response to the knockdown of Chk1 (**Fig. 8B**) or that of Bub1, BubR1, Mad2 or AuroraB (**Fig. 8C**). In each case, the inhibition of p53 strongly attenuated the cytotoxic effects of cisplatin and/or SAC inhibition.

Next, we downregulated a series of p53 target genes that were strongly induced by Chk1 inhibition in tetraploid cells (**Table S1**). Knockdown of the BH3-only protein Puma/BBC3, of the Bcl-2 antagonist Bax, or of the pro-apoptotic transcription factor ATF3 [36] (**Fig. S3**) attenuated the cytotoxic effect of Chk1 inhibition in tetraploid cells, while the depletion of villin 3 (ezrin), spermidine/spermine N1-acetyltransferase (SSAT) or of the essential autophagy gene Atg12 had no such effect (**Fig. 8D**). These results suggested that the Bcl-2 protein family dictates the fate of Chk1-inhibited tetraploid cells. Accordingly, knockdown of Puma/BBC3 and of the pro-apoptotic multi-domain Bcl-2 family proteins Bax and Bak were as efficient in inhibiting Chk1 depletion-induced cell death as was the downregulation of p53 and the p53 associated protein p53BP1 (**Fig. 8E**). These data could be corroborated at the genetic level. Tetraploid cells derived from parental Bax^−/−^ HCT116, generated as previously described [8], cells failed to die in response to Chk1 depletion (**Fig. 8F**). Moreover, knockdown of the endogenous Bax/Bak antagonists Bcl-2 or Bcl-X_L_ sensitized tetraploid cells to Chk1 inhibition (Fig. 8E). Altogether, these results indicate that Chk1 inhibition kills tetraploid cells via the activation of a p53-dependent pathway that elicits mitochondrial membrane permeabilization through the induction of Puma, which in turn acts on its mitochondrial receptors Bax and Bak.

## Discussion

In the present study, we show that inhibition of Chk1 by small interfering RNAs, dominant-negative mutant Chk1, or pharmacological compounds is particularly toxic on tetraploid tumor cells that otherwise are relatively resistant against genotoxic agents. The mechanism accounting for the preferential killing of tetraploid cells appears to involve the abolition of the spindle assembly checkpoint (SAC), premature entry in mitosis, failed and catastrophic mitotic events, as well as p53 activation, transcription of pro-apoptotic p53 target genes including the BH3 only protein Puma and induction of the mitochondrial pathway of apoptosis.

Although this sequence of events is well documented in this paper, it is at variance with previous knowledge on cell death induction by Chk1 inhibition. First, Chk1 activation reportedly causes the phosphorylation of p53, resulting in its stabilization and its transcriptional activation [37]. On the contrary, here we show that the inhibition of Chk1 can activate p53, as indicated by an increased p53 phosphorylation and an increased transcription of p53 target genes. Second, Chk1 inhibition by UCN-01 or other chemical Chk1 inhibitors has been shown to sensitize preferentially p53-deficient cells [38]–[40]. In contrast with this notion, we show here that, at least for tetraploid cells, p53 activation is required for the cytotoxic effect of Chk1 inhibition. Thus, inhibition of p53 (chemically or genetically, by knockdown or knockout) strongly reduced cell killing by Chk1 inhibition or depletion.

What may be the mechanism through which Chk1 inhibition is particularly toxic for tetraploid cells? Reportedly, Chk1 is required for optimal progression of replication forks during normal S phase of vertebrate cells [41] and avoids the accumulation of DNA breaks at fragile sites [42]. On theoretical grounds, the replication of a tetraploid set of chromosomes may be particularly vulnerable to perturbations, which ultimately results in delayed or incomplete DNA replication followed by premature and catastrophic mitosis. Chk1 depletion can favor the premature activation of the Cdk1/cyclin B1 complex [43], before the completion of the S phase, which leads to mitotic defects with chromosome misalignment during metaphase, chromosome lagging during anaphase, and kinetochore defects within the regions of misaligned/lagging chromosomes [44]. Hence, Chk1 depletion may amplify the natural tendency of tetraploid cells to die during or shortly after mitosis.

There is an alternative and additional explanation for the preferential toxicity of Chk1 inhibition on tetraploid cells that is supported by our data. Chk1 is required for spindle checkpoint function, both in normal and in taxol-treated cells [45]. As shown here, tetraploid cells have an intrinsic propensity to undergo mitotic catastrophe although they have an intact spindle assembly checkpoint (SAC) and even tend to spontaneously activate SAC. Chk1 depletion compromises the spindle checkpoint by negatively affecting the Aurora-B-dependent recruitment of BubR1 to kinetochores [45]. We have confirmed this finding, showing that Chk1 inhibition prevents the recruitment of both BubR1 and Bub1 to the centromeres of tetraploid cells during the prometaphase or aberrant metaphases. Moreover, we have found that inhibition of SAC by depletion of essential SAC proteins (such as Bub1, BubR1 or Mad2) or inhibition of Aurora-B had similar effects as Chk1 inhibition, namely a selective toxicity for tetraploid as compared to diploid cells. The abolition of SAC killed tetraploid cells through the activation of p53, exactly as this has been observed for Chk1 inhibition. Chk1 inhibition caused the abrogation of SAC both in diploid and tetraploid cell lines; nevertheless, SAC is more frequently activated in untreated tetraploid cells, correlating with an elevated frequency of abnormal mitoses. Hence, it appears plausible that Chk1 inhibition kills tetraploid cells through its capacity to abolish SAC and because tetraploids rely more heavily on an intact SAC than diploid cells.

Chk1 inhibitors including UCN-01 are being introduced into clinical trials [24] in combination with genotoxic agents such as cytarabine (for the treatment of acute myeloid leukemia) [46] or cisplatin (for the treatment of advanced solid tumors) [47], and proof has been obtained that UCN-01 can actually inhibit Chk1 *in vivo*, in tumor cells [46], although the inhibitor may act on other kinases including Chk2 and PDK1 as well, at least *in vitro*. As shown here, Chk1 inhibition by UCN-01 sensitizes tetraploid cells to cisplatin-induced cell death, rendering otherwise chemoresistant tetraploid tumors amenable to treatment, *in vivo*, in a model of xenotransplanted human tumors. The mechanism through which Chk1 inhibition (by UCN-01 or by two distinct specific siRNAs) can restore the cisplatin sensitivity of tetraploid cells is elusive. Based on the microarray data and cytotoxic effects, it appears that diploid cells are relatively refractory to the apoptosis-inducing and to the transcriptome-modifying effects of Chk1 inhibition (as compared to tetraploid cells), while tetraploid cells are relatively resistant against the cytocidal and transcriptional effect of cisplatin (as compared to diploid cells). The combination of both cisplatin and Chk1 inhibition had very similar effects on diploid and tetraploid cells in thus far that it provoked cell death at a substantially similar level and regulated a largely overlapping panel of genes at the transcriptional level. Apparently, p53 target genes are particularly important in mediating the combined lethal effect of cisplatin and Chk1 inhibition, both in diploid and tetraploid cells. The DNA damage-elicited kinase ATR is elicited by cisplatin [48], [49] and may participate in the activation of Chk1, which in turn mediates the cisplatin-induced cell cycle arrest [50], [51]. Hence, inhibition of Chk1 may subvert a DNA damage-elicited cell cycle checkpoint, thereby preventing DNA repair and favoring an apoptotic response.

The challenge for future studies will be to determine whether other specific Chk1 inhibitors can overcome chemoresistance of tumor cells *in vivo*, in suitable preclinical models as well as in clinical trials. It will be particularly interesting to determine whether Chk1 inhibitors can be used for the treatment or even for the prophylaxis of polyploidization-associated neoplasias.

## Materials and Methods

### Cell lines, culture, transfection and siRNA

Tetraploid cells were generated from diploid WT, p53^−/−^, Bax^−/−^ (gift from Bert Vogelstein) HCT116 and RKO diploid precursor as previously described in [8], [35]. Diploid or tetraploid HCT116 cells were grown in McCoy's 5A medium supplemented with 10% FCS. RKO clones transfected with the cDNA encoding H2B-GFP were cultured in the presence of blasticidine (20 µg/ml). All media and supplements for cell culture were purchased from Gibco-Invitrogen (Carlsbad, USA). For each experiment at least three clones diploid and tetraploid were used. Subconfluent cultures were cultured in the absence or in the presence of cisplatin (20 µM, Sigma-Aldrich, St. Louis, USA) and/or Chk1 inhibitors (UCN-01 from the NCI, USA; SD1825, from Servier, Neuilly-sur-Seine, France, see **Fig. S4**, IC_50_ for recombinant Chk1 *in vitro*: 29 nM) at indicated concentrations for 24–48 hours, in the presence or in the absence of the pan-caspase inhibitor Z-valine-alanine-aspartate- fluoromethyl ketone (Z-VAD-fmk, 100 µM) or of the p53 inhibitor cyclic pifithrin-α (20 µM). Cells were transfected with pRc/CMV containing human wild-type p53 (WT) or mutant (H175) p53 (gift of T. Soussi, Institut Curie, Paris, France) under the control of the cytomegalovirus (CMV) promoter [52]. Alternatively, cells were transfected with CMV-driven expression plasmids containing WT or DN Chk1 (kinase dead, gift by K.K. Khanna) [53], WT or DN Chk2 [54], or a p53-inducible green fluorescence protein (GFP) plasmid (gift from K. Wiman, Karolinska Cancer Center, Stockolm, Sweden) [55]. Transfections were performed by adding lipofectamine™ 2000 (Invitrogen) complexed with plasmid (final concentration: 1 µg/ml) 24 hours before the addition of cyclic pifithrin-α (15 µM; Sigma-Aldrich) or cisplatin (20 µM; Sigma-Aldrich). In some experiments, transfections were performed in the presence of pDsRed2 vector (0.2 µg/ml; Clontech, Palo Alto, USA) to identify transfected cells. After additional 48 hours, cells were subjected to cytofluorometric analysis of viability.

The knock-down of Atg12, ATF3, Aurora B, Bad, Bax a, Bcl-2, Bcl-X_L_, Bid, Bub1, BubR1, Chk1, Chk2, Mad2, p53, p53BP1, PUMA, spermidine/spermine N1-acetyltransferase (SSAT), Survivin, VDAC1, villin 3 (ezrin), was performed with siRNAs purchased from Sigma-Proligo (The Woodlands, USA, siRNA sequences in **Table S2**); the down-regulation of Bak and Bax was performed with siRNAs (Hs_BAK_5, Hs_BAX_5 and Hs_BAX_10 HP Validated siRNAs, respectively) purchased from Qiagen (Hilden, Germany). As a control, a siRNA specific for emerin [56] as well as unrelated control siRNAs (scramble, SCR) were used. Cells were cultured in 12-well plates and transfected at 30–40% confluence by adding HiPerFect (Qiagen, Hilden, Germany) complexed with siRNA (final concentration: 20nM) as described previously [57]. 72 h later, the efficiency of transfection was determined by immunoblot.

### Measurement of cell viability with a tetrazolium reduction assay

3.5×10^3^ HCT116 cells were seeded in 100 µl of McCoy's 5A medium in 96-well plates and transfected after 24h, as follows. 2.5 pmol of each siRNA plus 2.5 pmol of Chk-1 specific siRNA or control siRNA (SCR), dissolved in 10 µl of serum-free, antibiotic-free, DMEM:F12 (1∶1) with L-glutamine but no phenol red were mixed with 0.5 µl of HiPerFect transfection reagent (Qiagen) dissolved in 10 µl of the same medium and let stand at room temperature for 30 min. Thereafter, transfection complexes (20 µl) were added to the cultures. Transfected cells were maintained for 48 h prior to cell viability assays based on the cleavage of the tetrazolium salt WST-1 (Roche Diagnostics, Germany).

### Staining of live cells, immunofluorescence and videomicroscopy

For the simultaneous assessment of mitochondrial apoptosis and plasma membrane permeabilization, live cells were stained with 3,3′dihexiloxalocarbocyanine iodide or tetramethylrhodamine methylester (DiOC_6_(3) 40 nM or TMRM 150 nM, emitting in green or in red respectively; Molecular Probes-Invitrogen) which measure ΔΨ_m_, and propidium iodide (PI, 2 µg/ml, Sigma-Aldrich) or 4′,6-diamidino-2-phenylindole (DAPI, 10 µM, Molecular Probes-Invitrogen), vital dyes that incorporate only into dead cells), for 30 min at 37°C [58]. Exposure of phosphatidylserine (PS) was evaluated using annexin V-FITC (Molecular Probes, Invitrogen). For simultaneous measurement of DNA content and Histone H3 phosphorylation, cells were fixed with ethanol (70% v:v), permeabilized with tween (0.25% v:v) and stained with DAPI (10 µM, Molecular Probes-Invitrogen) and a rabbit antisera specific for Histone H3 phosphorylated (Upstate, Lake Placid, USA). Cytofluorometric analyses were performed on a FACS Vantage (Becton Dickinson, San Jose, USA) equipped with a 70 µm nozzle. Data were statistically evaluated using Cell Quest software (Becton Dickinson).

Alternatively, for fluorescence microscopy, cells were fixed with paraformaldehyde (4% w:v) then stained with rabbit antisera specific for p53 phophorylated on serine 15 (Cell Signaling Technology Inc., Danvers, USA), and for activated caspase-3 (Casp-3a, Cell Signaling Technology Inc.) or with mouse antisera specific for cytochrome *c* (cyt c, BD Biosciences, San Jose, USA), β-tubulin, or γ-tubulin (both from Sigma-Aldrich) [7], [59].

To detect Bub1, BubR1 and CENP-B, cells were fixed in 4% (w/v) paraformaldehyde in PIPES buffer (80 mM PIPES, 5 mM EGTA, 2 mM MgCl2) and co-imunostained with antibodies that recognize Bub1 (rabbit polyclonal IgG, Chemicon International, Temecula, USA), BubR1 (mouse monoclonal antibody, BD Biosciences) and CENP-B (mouse monoclonal IgG, Santa Cruz Biotechnology, San Jose, USA) (Vitale et al., 2007).

After the incubation with the primary antibody, slides were incubated with goat anti-rabbit or anti-mouse IgG conjugated to Alexa 568 or to Alexa 488 fluorochrome (Molecular Probes-Invitrogen).

Chromatin was stained with Hoechst 33342 (2 µM, Molecular Probes-Invitrogen). RKO cells expressing histone H2B-GFP were cultured in the 35mm glass bottom culture dishes (MatTek Corporation, Ashland, USA), maintained at a constant temperature of 37°C in an atmosphere with 5% CO2, and were subjected to pulsed observations using an LSM 510 laser-scanning confocal microscope (Zeiss, Oberkochen, Germany).

### Quantitation of protein expression

Protein samples were prepared from HCT116 or RKO cells in lysis buffer. Aliquots of protein extracts (50 µg/lane) were subjected to immunoblots using antibodies specific for Bcl-2 (Santa Cruz Biotechnology), Bub1 (Chemicon International), CENP-B (Santa Cruz Biotechnology), BubR1 (BD Biosciences), Chk1 (Santa Cruz Biotechnology), Chk2 (Upstate), Mad2 (Santa Cruz Biotechnology), VDAC (anti-porin 31HL, Calbiochem International, Temecula, USA) and GAPDH (Chemicon, CA) monitored as loading control.

### Microarray analyses

We used Agilent long (60bp) oligonucleotide microarrays and the dual color analysis method in which probes from specimens and from the reference are differentially labeled with Cyanine 5 and Cyanine 3 as described previously (Castedo et al, 2006). We performed a set of 6 dye swap experiments to compare RNAs obtained, in each case, from tetraploid and diploid HCT116 cell lines cultured in the presence of cisplatin, UCN-01 and a combination of both. In each experiment, the reference was RNAs from cell lines without treatment. From each of the 6 combined experiments, signatures (list of accession numbers) at p-value of 10^−5^ are extracted and annotated with updated databases as Entrez-Gene. A list of 275 p53 regulated genes are defined in Ingenuity Pathways Analysis program (by a query of genes for which p53 directly interact with DNA, Ingenuity, Mountain View, USA, http://www.ingenuity.com/products/pathways_analysis.html).

### *In vivo* model

Athymic *nu/nu* six-week-old female mice (IGR animal facility) were inoculated s.c in 200 µl of PBS with 3×10^6^ diploid or tetraploid HCT116 cells into the lower flank as previously described [60]. When tumors reached 125 mm^3^, mice received i.p. either 200 µl of PBS1X, 5mg/kg of cisplatin three times a week during 3 weeks, UCN-01 (7.5 mg/kg/daily) for 5 consecutive days or a combination of cisplatin plus UCN-01. Tumor growth was evaluated twice a week using a caliper. The mean of the tumor volume at each point was normalized in each group to the mean volume measured at the first injection. All animals were maintained in specific pathogen-free conditions and all experiments followed the FELASA guidelines.

## Supporting Information

Table S1 (2.69 MB DOC)

Table S2 (0.07 MB DOC)

Figure S1 Intact SAC in tetraploid HCT116 cells. Diploid (D) or tetraploid (T) HCT116 cells were left untreated or cultured in the presence of docetaxel (1 µM) or nocodazole (1 µM) for the indicated period (24 h in A and C) and subjected to cell cycle analyses (A, B) by labeling with 4,6-diamidino-2-phenylindole (DAPI) or stained for the detection of phospho-histone H3, a histone that is specifically phosphorylated during mitosis (C, D). Representative FACS data are shown in A and C and quantitative data (X±SEM, n = 3) are shown in B and D. In addition the toxicity of docetaxel and nocodazole was determined by staining with DiOC6(3)/PI, yielding information on the frequency of dying (DiOC6(3)low PI-) or dead (DiOC6(3)low PI+) cells (E)
(2.88 MB TIF)

Figure S2 Apoptosis induction by depletion or inhibition of Chk1 in tetraploid RKO cells. A. Chk1 depletion kills tetraploid RKO colon cancer cells. RKO cells were transfected with siRNAs that deplete Chk1 (Chk1a) or Chk2 (Chk2a), as demonstrated by the immunoblot performed 48 h after transfection. The frequency of dying (ΔΨmlow) cells was determined by staining with tetramethyl rhodamine methylester (TMRM, 150 nM, 15 min). Asterisks mark significant (p<0.01) effects of Chk1 depletion. B. Chk1 inhibition by SD1825 kills tetraploid RKO cells. RKO cells were cultured with the indicated doses of SD1825, and the frequency of dead and dying cells was measured by simultaneous staining with DiOC6(3) and PI (as in Fig. 3A) 48 h later. C. Combined effects of Chk1 inhibition and cisplatin (20 µM) on tetraploid RKO cells. Diploid or tetraploid RKO cells were treated by siRNAs targeting emerin (as a negative control), VDAC1 (as a positive control of apoptosis inhibition), Chk1 or Chk2, followed by staining with TMRM to measure ΔΨm dissipation
(1.49 MB TIF)

Figure S3 The efficacy of the siRNAs specific for Puma, ATF3 and Bax was determined by immunoblot, 48 hours after transfection of tetraploid HCT116 cells with control scrambled (SCR) siRNA or the indicated specific siRNAs. The polyclonal rabbit antibodies specific for Puma, ATF3 and Bax were from Ψ ProSci Incorporated, Santa Cruz Biotechnology, and Upstate Biotechnology, respectively. Equal loading was determined with anti-actin antibody (monoclonal mouse IgG1 from AbCys)
(3.69 MB TIF)

Figure S4 Chemical structure of SD 1825 and its IC50 for recombinant Chk1
(0.96 MB TIF)

Video S1 Abnormal mitoses in untreated tetraploid RKO cells expressing histone H2B-GFP. The video shows an example of a cell that undergoes an apparently normal mitosis, followed by near-to-complete karyokinesis, furrow regression and formation of one single binucleate cell, as well as a cell arrested in metaphase
(1.74 MB AVI)

Video S2 Abnormal mitosis in untreated tetraploid RKO cells expressing histone H2B-GFP. The video exemplifies an aborted cell division leading to the formation of a single daughter cell
(1.53 MB AVI)

Video S3 Normal mitosis in Chk-1 depleted tetraploid RKO cells expressing histone H2B-GFP. Selected pictures are shown in Fig. 2A, 0
(1.10 MB AVI)

Video S4 Delayed mitosis in Chk-1 depleted tetraploid RKO cells expressing histone H2B-GFP. Selected pictures are shown in Fig. 2A, I
(1.15 MB AVI)

Video S5 Delayed mitosis leading to apoptosis of both daughter cells in Chk-1 depleted tetraploid RKO cells expressing histone H2B-GFP. Selected pictures are shown in Fig. 2A, II
(0.61 MB AVI)

Video S6 Aberrant mitosis with an abnormal metaphase plate in Chk-1 depleted tetraploid RKO cells expressing histone H2B-GFP. Note that the final result of the process is the generation of one single binucleated cell. Selected pictures are shown in Fig. 2A, III
(1.83 MB AVI)

Video S7 Tripolar mitosis in Chk-1 depleted tetraploid RKO cells expressing histone H2B-GFP. Selected pictures are shown in Fig. 2A, IV
(1.07 MB AVI)

Video S8 Apoptosis during the metaphase of Chk-1 depleted tetraploid RKO cells expressing histone H2B-GFP. Selected pictures are shown in Fig. 2A, V
(1.74 MB AVI)
