# Supplementary material for: Inhibition of Chk1 Kills Tetraploid Tumor Cells through a p53-Dependent Pathway
Source: PLoS One. 2007 Dec 26;2(12):e1337. doi: 10.1371/journal.pone.0001337 (PMC2131784; doi:10.1371/journal.pone.0001337)
Supplement: Table S1 — (2.69 MB DOC) [file pone.0001337.s001.doc]

| **Table S1:** | | | | |
| --- | --- | --- | --- | --- |
|  | | | | |
| **A. Modification of gene expression by Chk1 inhibition in diploid and tetraploid cells** | | | | |
|  |  |  |  |  |
| **Chk1 inhibitor Diploid specific** | | | | |
|  |  |  |  |  |
| **Accession** | **Symbol** |  | **FoldChangeD** | **p53 regulated** |
|  |  |  |  |  |
| NM_001511 | CXCL1 |  | **2.21** | - |
| NM_005581 | BCAM |  | **2.16** | - |
| NM_005532 | IFI27 |  | **1.74** | - |
| NM_003641 | IFITM1 |  | **1.56** | - |
| AF220415 | - |  | **1.53** | - |
| NM_001548 | IFIT1 |  | **1.50** | - |
| NM_005573 | LMNB1 |  | **1.35** | - |
| NM_022873 | IFI6 |  | **1.34** | - |
| NM_005319 | HIST1H1C |  | **1.30** | - |
| NM_023080 | C8orf33 |  | **1.25** | - |
| NM_022117 | TSPYL2 |  | **-1.21** | - |
| AF132203 | SCD |  | **-1.24** | - |
| NM_145244 | DDIT4L |  | **-1.27** | - |
| NM_000782 | CYP24A1 |  | **-1.28** | - |
| AI792678 | - |  | **-1.31** | - |
| NM_033251 | RPL13 |  | **-2.09** | - |
|  |  |  |  |  |
| **Chk1 inhibitor Common** | | | | |
|  |  |  |  |  |
| **Accession** | **Symbol** | **FoldChangeT** | **FoldChangeD** | **p53 regulated** |
|  |  |  |  |  |
| NM_024508 | ZBED2 | **-1.32** | **1.45** | - |
| NM_014260 | PFDN6 | **1.25** | **1.37** | - |
| NM_006509 | RELB | **1.43** | **1.32** | - |
| BX538238 | MALAT1 | **-1.29** | **1.32** | - |
| NM_005101 | ISG15 | **1.33** | **1.28** | - |
| NM_002952 | RPS2 | **-2.12** | **-2.42** | - |
|  |  |  |  |  |
| **Chk1 inhibitor Tetraploid specific** | | | | |
|  |  |  |  |  |
| **Accession** | **Symbol** | **FoldChangeT** |  | **p53 regulated** |
|  |  |  |  |  |
| AB033060 | AHRR | **2.74** |  | - |
| NM_015508 | TIPARP | **2.45** |  | - |
| NM_000693 | ALDH1A3 | **2.43** |  | - |
| NM_000499 | CYP1A1 | **2.30** |  | - |
| NM_005329 | HAS3 | **2.26** |  | - |
| NM_003714 | STC2 | **2.01** |  | - |
| NM_003379 | VIL2 | **1.97** |  | VIL2 |
| AL833872 | - | **1.94** |  | - |
| NM_032957 | - | **1.81** |  | - |
| BC033829 | - | **1.75** |  | - |
| CR627234 | - | **1.71** |  | - |
| NM_002010 | FGF9 | **1.70** |  | - |
| AF086414 | - | **1.68** |  | - |
| NM_004864 | GDF15 | **1.64** |  | GDF15 |
| NM_001964 | EGR1 | **1.64** |  | - |
| NM_002228 | JUN | **1.61** |  | - |
| AK027191 | - | **1.58** |  | - |
| NM_001450 | - | **1.57** |  | - |
| NM_004293 | GDA | **1.56** |  | - |
| NM_005902 | SMAD3 | **1.53** |  | - |
| NM_138931 | BCL6 | **1.52** |  | - |
| NM_005318 | H1F0 | **1.51** |  | - |
| NM_004031 | IRF7 | **1.51** |  | - |
| BC004960 | MGC10955 | **1.51** |  | - |
| NM_001001552 | LEMD1 | **1.50** |  | - |
| NM_024101 | MLPH | **1.50** |  | - |
| NM_003786 | - | **1.46** |  | - |
| NM_003897 | IER3 | **1.46** |  | - |
| NM_144497 | AKAP12 | **1.46** |  | AKAP12 |
| NM_005953 | MT2A | **1.45** |  | - |
| NM_004024 | ATF3 | **1.45** |  | ATF3 |
| BC007034 | - | **1.45** |  | - |
| NM_004561 | OVOL1 | **1.45** |  | - |
| NM_182532 | TMEM61 | **1.45** |  | - |
| NM_000228 | - | **1.44** |  | - |
| NM_002970 | SAT | **1.43** |  | SAT |
| NM_002309 | LIF | **1.42** |  | LIF |
| NM_014417 | BBC3 | **1.42** |  | BBC3 |
| NM_002402 | MEST | **1.41** |  | - |
| NM_005952 | MT1X | **1.41** |  | - |
| NM_183422 | TSC22D1 | **1.40** |  | - |
| NM_005950 | MT1G | **1.40** |  | - |
| NM_017983 | WIPI1 | **1.39** |  | - |
| NM_004695 | SLC16A5 | **1.38** |  | - |
| NM_175622 | MT1JP | **1.37** |  | - |
| NM_020947 | KIAA1609 | **1.36** |  | - |
| NM_005947 | MT1B | **1.36** |  | - |
| NM_021005 | NR2F2 | **1.36** |  | - |
| X97261 | MT1L | **1.35** |  | - |
| AL833749 | LOC146439 | **1.35** |  | - |
| NM_006516 | SLC2A1 | **1.35** |  | SLC2A1 |
| NM_005951 | MT1H | **1.34** |  | - |
| NM_033520 | C19orf33 | **1.34** |  | - |
| NM_006096 | NDRG1 | **1.34** |  | NDRG1 |
| NM_024928 | OBFC1 | **1.34** |  | - |
| NM_014316 | CARHSP1 | **1.33** |  | - |
| NM_031213 | FAM108A1 | **1.33** |  | - |
| XM_372040 | - | **1.33** |  | - |
| NM_148965 | - | **1.33** |  | - |
| NM_175617 | MT1E | **1.33** |  | - |
| NM_003315 | DNAJC7 | **1.33** |  | - |
| AK125231 | - | **1.31** |  | - |
| BC069216 | - | **1.31** |  | - |
| NM_001673 | ASNS | **1.31** |  | - |
| AA307830 | GAS5 | **1.30** |  | - |
| NM_016270 | KLF2 | **1.29** |  | - |
| NM_002658 | PLAU | **1.28** |  | - |
| NM_004707 | ATG12 | **1.28** |  | - |
| NM_001195 | BFSP1 | **1.28** |  | - |
| NM_021199 | SQRDL | **1.28** |  | - |
| NM_031858 | NBR1 | **1.27** |  | - |
| NM_153256 | C10orf47 | **1.27** |  | - |
| NM_002751 | MAPK11 | **1.27** |  | - |
| NM_002306 | LGALS3 | **1.27** |  | - |
| NM_032947 | MST150 | **1.26** |  | - |
| NM_176870 | MT1M | **1.26** |  | - |
| NR_002205 | FTHL12 | **1.26** |  | - |
| NM_024897 | PAQR6 | **1.26** |  | - |
| NM_002032 | FTH1 | **1.26** |  | - |
| NM_144505 | KLK8 | **1.25** |  | - |
| NM_021173 | POLD4 | **1.24** |  | - |
| NM_005693 | NR1H3 | **1.23** |  | - |
| NM_016491 | MRPL37 | **-1.21** |  | - |
| NM_012458 | TIMM13 | **-1.21** |  | - |
| NM_001905 | CTPS | **-1.22** |  | - |
| NM_002106 | H2AFZ | **-1.22** |  | - |
| BF368083 | - | **-1.23** |  | - |
| NM_005654 | NR2F1 | **-1.23** |  | - |
| BC063888 | - | **-1.23** |  | - |
| NM_004559 | YBX1 | **-1.24** |  | - |
| NM_003113 | SP100 | **-1.24** |  | - |
| NM_031844 | HNRPU | **-1.24** |  | - |
| NM_181578 | RFC5 | **-1.24** |  | - |
| NM_002916 | RFC4 | **-1.24** |  | RFC4 |
| NM_004642 | CDK2AP1 | **-1.25** |  | - |
| NM_005520 | HNRPH1 | **-1.25** |  | - |
| NM_001444 | FABP5 | **-1.25** |  | - |
| NM_012291 | ESPL1 | **-1.25** |  | - |
| NM_002452 | - | **-1.25** |  | - |
| NM_002539 | ODC1 | **-1.26** |  | - |
| NM_003134 | SRP14 | **-1.26** |  | - |
| NM_153201 | HSPA8 | **-1.26** |  | HSPA8 |
| NM_007086 | WDHD1 | **-1.26** |  | - |
| NM_017751 | - | **-1.26** |  | - |
| NM_002128 | HMGB1 | **-1.27** |  | - |
| NM_003539 | HIST1H4D | **-1.27** |  | - |
| XM_497271 | HMGB1 | **-1.27** |  | - |
| NM_005441 | CHAF1B | **-1.28** |  | - |
| NM_182687 | PKMYT1 | **-1.28** |  | - |
| NM_006807 | CBX1 | **-1.28** |  | - |
| NM_001069 | TUBB2A | **-1.30** |  | - |
| NM_005782 | THOC4 | **-1.30** |  | - |
| NM_005348 | HSP90AA1 | **-1.30** |  | HSP90AA1 |
| NM_012124 | CHORDC1 | **-1.31** |  | - |
| NM_001101 | ACTB | **-1.31** |  | - |
| NM_001540 | HSPB1 | **-1.31** |  | HSPB1 |
| NM_018101 | CDCA8 | **-1.31** |  | - |
| NM_022346 | HCAP-G | **-1.32** |  | - |
| NM_003504 | CDC45L | **-1.32** |  | - |
| NM_014750 | DLG7 | **-1.32** |  | - |
| NM_018685 | ANLN | **-1.32** |  | - |
| NM_177987 | TUBB8 | **-1.33** |  | - |
| NM_001255 | CDC20 | **-1.33** |  | - |
| NM_005483 | CHAF1A | **-1.33** |  | - |
| NM_032525 | TUBB6 | **-1.33** |  | - |
| NM_014736 | KIAA0101 | **-1.35** |  | - |
| NM_004526 | MCM2 | **-1.35** |  | - |
| NM_001379 | DNMT1 | **-1.36** |  | - |
| NM_207312 | - | **-1.36** |  | - |
| NM_178014 | TUBB | **-1.36** |  | - |
| BC046178 | - | **-1.36** |  | - |
| NM_007355 | HSP90AB1 | **-1.36** |  | HSP90AB1 |
| NM_006597 | HSPA8 | **-1.36** |  | HSPA8 |
| NM_006000 | TUBA1 | **-1.36** |  | - |
| CR616772 | HSP90AB1 | **-1.36** |  | HSP90AB1 |
| BF511442 | HIST1H2BD | **-1.37** |  | - |
| NM_025019 | TUBA4 | **-1.37** |  | - |
| NM_014791 | MELK | **-1.37** |  | - |
| NM_032636 | PSRC1 | **-1.37** |  | - |
| NM_002388 | MCM3 | **-1.37** |  | - |
| NM_015161 | ARL6IP | **-1.38** |  | ARL6IP |
| NM_001237 | CCNA2 | **-1.38** |  | - |
| NM_004260 | RECQL4 | **-1.38** |  | - |
| NM_007294 | - | **-1.38** |  | - |
| NM_006739 | MCM5 | **-1.38** |  | - |
| AI015919 | - | **-1.39** |  | - |
| NM_018492 | PBK | **-1.39** |  | - |
| NM_001009936 | PHF19 | **-1.40** |  | - |
| NM_003107 | SOX4 | **-1.40** |  | - |
| NM_006461 | SPAG5 | **-1.40** |  | - |
| NM_145697 | CDCA1 | **-1.40** |  | - |
| XM_293276 | - | **-1.40** |  | - |
| NM_031966 | CCNB1 | **-1.40** |  | CCNB1 |
| NM_002358 | MAD2L1 | **-1.41** |  | - |
| NM_032704 | TUBA6 | **-1.41** |  | - |
| NM_021992 | TMSL8 | **-1.41** |  | TMSL8 |
| NM_024094 | DCC1 | **-1.42** |  | - |
| NM_181803 | UBE2C | **-1.42** |  | - |
| NM_002129 | HMGB2 | **-1.42** |  | - |
| NM_016095 | GINS2 | **-1.43** |  | - |
| NM_015341 | BRRN1 | **-1.44** |  | - |
| NM_004111 | FEN1 | **-1.44** |  | - |
| BC000712 | - | **-1.44** |  | - |
| NM_013282 | UHRF1 | **-1.44** |  | - |
| NM_003686 | EXO1 | **-1.44** |  | - |
| NM_019013 | FAM64A | **-1.45** |  | - |
| NM_032997 | - | **-1.45** |  | - |
| NM_001168 | - | **-1.47** |  | - |
| NM_024745 | SHCBP1 | **-1.47** |  | - |
| NM_152562 | CDCA2 | **-1.48** |  | - |
| NM_001067 | TOP2A | **-1.48** |  | TOP2A |
| NM_014501 | UBE2S | **-1.48** |  | - |
| NM_006082 | K-ALPHA-1 | **-1.49** |  | - |
| NM_030919 | FAM83D | **-1.49** |  | - |
| NM_031299 | CDCA3 | **-1.50** |  | - |
| NM_005733 | KIF20A | **-1.52** |  | - |
| NM_080668 | CDCA5 | **-1.52** |  | - |
| CR749222 | - | **-1.59** |  | - |
| BC044933 | - | **-1.61** |  | - |

| **B. Modification of gene expression by Cisplatin in diploid and tetraploid cells** | | | | |
| --- | --- | --- | --- | --- |
|  |  |  |  |  |
| **Cisplatin Diploid specific** | | | | |
|  |  |  |  |  |
| **Accession** | **Symbol** |  | **FoldChangeD** | **p53 regulated** |
|  |  |  |  |  |
| NM_020777 | SORCS2 |  | **5.98** | - |
| NM_012396 | PHLDA3 |  | **2.83** | PHLDA3 |
| NM_000584 | IL8 |  | **2.57** | - |
| NM_014508 | APOBEC3C |  | **2.52** | - |
| NM_004425 | ECM1 |  | **2.46** | - |
| NM_005532 | IFI27 |  | **2.44** | - |
| NM_057749 | CCNE2 |  | **2.36** | - |
| NM_003265 | TLR3 |  | **2.35** | - |
| NM_000526 | KRT14 |  | **2.34** | - |
| NM_007135 | ZNF79 |  | **2.26** | - |
| X82545 | - |  | **2.16** | - |
| CA450379 | - |  | **2.10** | - |
| NM_014578 | RHOD |  | **2.07** | - |
| NM_022767 | ISG20L1 |  | **1.99** | - |
| NM_182573 | LYPD5 |  | **1.99** | - |
| NM_005072 | SLC12A4 |  | **1.98** | - |
| NM_002231 | CD82 |  | **1.98** | - |
| NM_007312 | HYAL1 |  | **1.95** | - |
| NM_004292 | RIN1 |  | **1.92** | - |
| NM_001002914 | KCTD11 |  | **1.92** | - |
| NM_178564 | NRBP2 |  | **1.90** | - |
| NM_018265 | C1orf106 |  | **1.89** | - |
| NM_014398 | LAMP3 |  | **1.89** | - |
| NM_199132 | ZNF468 |  | **1.87** | - |
| NM_000270 | NP |  | **1.86** | - |
| NM_001312 | CRIP2 |  | **1.85** | - |
| NM_176096 | - |  | **1.85** | - |
| BX648822 | CEP152 |  | **1.84** | - |
| NM_022873 | IFI6 |  | **1.84** | - |
| NM_002344 | LTK |  | **1.84** | - |
| NM_001345 | - |  | **1.84** | - |
| NM_016291 | IHPK2 |  | **1.82** | - |
| NM_153687 | IKIP |  | **1.81** | - |
| XR_000211 | - |  | **1.80** | - |
| AK054960 | - |  | **1.78** | - |
| CD359021 | - |  | **1.76** | - |
| AL117661 | - |  | **1.75** | - |
| NM_002275 | KRT15 |  | **1.75** | KRT15 |
| BC039151 | - |  | **1.75** | - |
| NM_019112 | ABCA7 |  | **1.75** | - |
| NM_002123 | HLA-DQB1 |  | **1.75** | - |
| NM_001003845 | SP5 |  | **1.74** | - |
| NM_018602 | DNAJA4 |  | **1.72** | - |
| NM_207380 | FLJ43339 |  | **1.72** | - |
| NM_021114 | SPINK2 |  | **1.71** | - |
| NM_003302 | TRIP6 |  | **1.71** | - |
| NM_006019 | TCIRG1 |  | **1.71** | - |
| NM_005755 | EBI3 |  | **1.70** | - |
| NM_002250 | KCNN4 |  | **1.70** | - |
| NM_004628 | XPC |  | **1.69** | XPC |
| AB002391 | - |  | **1.69** | - |
| AF318360 | MNT |  | **1.68** | - |
| NM_031207 | HYI |  | **1.68** | - |
| NM_019600 | KIAA1370 |  | **1.68** | - |
| NM_175744 | RHOC |  | **1.68** | - |
| NM_173714 | - |  | **1.68** | - |
| NM_002997 | - |  | **1.68** | - |
| NM_178453 | MGC52282 |  | **1.67** | - |
| NM_001531 | MR1 |  | **1.67** | - |
| NM_007026 | DUSP14 |  | **1.66** | - |
| NM_013290 | PSMC3IP |  | **1.65** | - |
| NM_018425 | PI4KII |  | **1.65** | - |
| AL832613 | HCP1 |  | **1.65** | - |
| NM_138934 | EGFL8 |  | **1.65** | - |
| NM_133180 | EPS8L1 |  | **1.65** | - |
| NM_138764 | BAX |  | **1.64** | BAX |
| NM_021980 | - |  | **1.63** | - |
| NM_001550 | - |  | **1.63** | - |
| NM_033388 | ATG16L2 |  | **1.63** | - |
| NM_002391 | - |  | **1.62** | - |
| NM_000676 | ADORA2B |  | **1.62** | - |
| NM_003516 | HIST2H2AA3 |  | **1.62** | - |
| NM_004417 | DUSP1 |  | **1.62** | DUSP1 |
| NM_024009 | GJB3 |  | **1.61** | - |
| AF132197 | - |  | **1.61** | - |
| NM_014376 | CYFIP2 |  | **1.60** | CYFIP2 |
| NM_021260 | ZFYVE1 |  | **1.60** | - |
| NM_004295 | TRAF4 |  | **1.60** | TRAF4 |
| BC015064 | TOB1 |  | **1.60** | - |
| AK057241 | - |  | **1.60** | - |
| NM_015003 | - |  | **1.60** | - |
| NM_001976 | ENO3 |  | **1.59** | - |
| NM_000135 | FANCA |  | **1.59** | - |
| NM_001172 | ARG2 |  | **1.59** | - |
| BC002881 | C15orf42 |  | **1.59** | - |
| NM_016323 | HERC5 |  | **1.58** | - |
| NM_001034 | RRM2 |  | **1.58** | - |
| NM_152718 | VWCE |  | **1.57** | - |
| M28016 | - |  | **1.57** | - |
| NM_020998 | MST1 |  | **1.57** | MST1 |
| NM_004091 | E2F2 |  | **1.57** | - |
| NM_022092 | CHTF18 |  | **1.57** | - |
| NM_021020 | LZTS1 |  | **1.57** | - |
| NM_012079 | DGAT1 |  | **1.56** | - |
| NM_000213 | ITGB4 |  | **1.56** | - |
| NM_004699 | FAM50A |  | **1.56** | - |
| NM_138809 | LOC134147 |  | **1.56** | - |
| NM_001321 | CSRP2 |  | **1.55** | - |
| NM_017818 | WDR8 |  | **1.55** | - |
| NM_134270 | - |  | **1.54** | - |
| AF216862 | - |  | **1.54** | - |
| AY029066 | - |  | **1.54** | - |
| NM_021820 | C6orf75 |  | **1.54** | - |
| AL137347 | - |  | **1.54** | - |
| AL365412 | - |  | **1.54** | - |
| NM_172229 | - |  | **1.54** | - |
| NM_014734 | KIAA0247 |  | **1.53** | - |
| NM_004370 | COL12A1 |  | **1.53** | - |
| NM_002999 | SDC4 |  | **1.53** | - |
| NM_007283 | MGLL |  | **1.53** | - |
| AK128746 | - |  | **1.53** | - |
| NM_006763 | BTG2 |  | **1.53** | BTG2 |
| NM_013299 | SAC3D1 |  | **1.53** | - |
| NM_006315 | PCGF3 |  | **1.53** | - |
| NM_153213 | ARHGEF19 |  | **1.52** | - |
| AF277181 | - |  | **1.52** | - |
| NM_005501 | - |  | **1.52** | - |
| NM_003844 | TNFRSF10A |  | **1.51** | TNFRSF10A |
| NM_030949 | PPP1R14C |  | **1.51** | - |
| NM_006291 | TNFAIP2 |  | **1.51** | TNFAIP2 |
| NM_006253 | PRKAB1 |  | **1.50** | - |
| NM_173466 | - |  | **1.50** | - |
| NM_002178 | IGFBP6 |  | **1.49** | - |
| AK027463 | - |  | **1.49** | - |
| NM_145906 | RIOK3 |  | **1.49** | - |
| XM_370839 | LOC653061 |  | **1.48** | - |
| AL137398 | - |  | **1.48** | - |
| NM_005379 | MYO1A |  | **1.48** | - |
| NM_198471 | ANKRD47 |  | **1.48** | - |
| NM_139160 | DEPDC7 |  | **1.48** | - |
| BC079831 | LOC284184 |  | **1.48** | - |
| NM_002960 | S100A3 |  | **1.47** | - |
| NM_173475 | DCUN1D3 |  | **1.47** | - |
| NM_001122 | ADFP |  | **1.47** | - |
| NM_198284 | LOC349114 |  | **1.47** | - |
| BC037255 | LOC389634 |  | **1.47** | - |
| NM_001197 | BIK |  | **1.47** | BIK |
| NM_014045 | LRP10 |  | **1.47** | - |
| AF391805 | - |  | **1.46** | - |
| NM_024733 | ZNF665 |  | **1.46** | - |
| NM_175047 | - |  | **1.46** | - |
| NM_024663 | NPEPL1 |  | **1.46** | - |
| NM_198489 | CCDC84 |  | **1.46** | - |
| NM_019601 | SUSD2 |  | **1.46** | - |
| NM_015257 | KIAA0286 |  | **1.45** | - |
| NM_001628 | AKR1B1 |  | **1.45** | - |
| NM_144707 | PROM2 |  | **1.45** | - |
| NM_001033 | RRM1 |  | **1.45** | - |
| BC063625 | KRTAP2-4 |  | **1.44** | - |
| CR602592 | - |  | **1.44** | - |
| NM_002876 | RAD51C |  | **1.44** | - |
| NM_144576 | COQ10A |  | **1.44** | - |
| NM_002292 | LAMB2 |  | **1.44** | - |
| NM_021972 | SPHK1 |  | **1.44** | - |
| NM_000407 | GP1BB |  | **1.44** | - |
| NM_016282 | AK3 |  | **1.43** | - |
| NM_003331 | TYK2 |  | **1.43** | - |
| CV815552 | - |  | **1.43** | - |
| AF187554 | MALAT1 |  | **1.43** | - |
| NM_001102 | ACTN1 |  | **1.43** | - |
| BC038432 | - |  | **1.43** | - |
| NM_203282 | ZNF539 |  | **1.43** | - |
| NM_032951 | MLXIPL |  | **1.42** | - |
| NM_001983 | ERCC1 |  | **1.42** | ERCC1 |
| NM_005402 | RALA |  | **1.42** | - |
| NM_017656 | ZNF562 |  | **1.42** | - |
| NM_015655 | ZNF337 |  | **1.42** | - |
| NM_030937 | CCNL2 |  | **1.42** | - |
| NM_001831 | - |  | **1.41** | - |
| NM_019848 | SLC10A3 |  | **1.41** | - |
| NM_032865 | TNS4 |  | **1.41** | - |
| NM_033306 | CASP4 |  | **1.41** | CASP4 |
| NM_198282 | LOC340061 |  | **1.41** | - |
| NM_012266 | DNAJB5 |  | **1.41** | - |
| NM_206920 | MAMDC4 |  | **1.41** | - |
| XM_499519 | - |  | **1.41** | - |
| NM_001067 | TOP2A |  | **1.41** | TOP2A |
| AF271776 | - |  | **1.41** | - |
| NM_022765 | MICAL1 |  | **1.41** | - |
| AK092942 | LOC653056 |  | **1.40** | - |
| NM_018943 | TUBA8 |  | **1.40** | - |
| NM_024015 | HOXB4 |  | **1.40** | - |
| NM_178546 | - |  | **1.40** | - |
| NM_004060 | CCNG1 |  | **1.40** | CCNG1 |
| NM_033120 | NKD2 |  | **1.40** | - |
| NM_145172 | WDR63 |  | **1.39** | - |
| AK098314 | - |  | **1.39** | - |
| NM_006647 | NOXA1 |  | **1.39** | - |
| NM_014316 | CARHSP1 |  | **1.39** | - |
| NM_138770 | - |  | **1.39** | - |
| NM_003166 | - |  | **1.39** | - |
| NM_017570 | OPLAH |  | **1.39** | - |
| AK097080 | LOC653340 |  | **1.39** | - |
| NM_139266 | STAT1 |  | **1.38** | - |
| NM_003979 | GPRC5A |  | **1.38** | - |
| NM_003315 | DNAJC7 |  | **1.38** | - |
| NM_000434 | NEU1 |  | **1.38** | - |
| NM_032854 | CORO6 |  | **1.38** | - |
| CR600908 | - |  | **1.38** | - |
| NM_003196 | TCEA3 |  | **1.38** | - |
| NM_012081 | ELL2 |  | **1.38** | - |
| NM_024798 | SNX22 |  | **1.38** | - |
| NM_032997 | - |  | **1.38** | - |
| NM_030927 | TSPAN14 |  | **1.38** | - |
| NM_032850 | ZFYVE19 |  | **1.37** | - |
| NM_014010 | ASTN2 |  | **1.37** | - |
| NM_138435 | FAM83F |  | **1.37** | - |
| AL136837 | DKFZp434F142 | | **1.37** | - |
| NM_005125 | CCS |  | **1.37** | - |
| NM_016573 | GMIP |  | **1.37** | - |
| NM_025149 | FLJ20920 |  | **1.37** | - |
| AY033611 | - |  | **1.37** | - |
| NM_017613 | DONSON |  | **1.37** | - |
| AY008274 | - |  | **1.36** | - |
| NM_001320 | CSNK2B |  | **1.36** | - |
| NM_001789 | CDC25A |  | **1.36** | - |
| NM_000156 | GAMT |  | **1.36** | - |
| NM_001932 | MPP3 |  | **1.36** | - |
| NM_000071 | CBS |  | **1.36** | - |
| NM_001958 | EEF1A2 |  | **1.36** | - |
| BI031372 | - |  | **1.35** | - |
| AK021957 | COL27A1 |  | **1.35** | - |
| NM_018454 | - |  | **1.35** | - |
| NM_005168 | RND3 |  | **1.35** | - |
| AK123446 | - |  | **1.35** | - |
| NM_018044 | - |  | **1.35** | - |
| NM_015219 | - |  | **1.35** | - |
| NM_001010914 | LOC400986 |  | **1.35** | - |
| NM_183372 | NBPF11 |  | **1.34** | - |
| NM_145645 | NSUN5B |  | **1.34** | - |
| NM_152858 | WTAP |  | **1.34** | - |
| NM_181471 | RFC2 |  | **1.34** | - |
| NM_007056 | SFRS16 |  | **1.34** | - |
| XM_497783 | LOC441964 |  | **1.34** | - |
| NM_016496 | MARCH2 |  | **1.34** | - |
| AK131226 | - |  | **1.34** | - |
| NM_133645 | - |  | **1.34** | - |
| CR613736 | KRT8 |  | **1.34** | - |
| AK057071 | - |  | **1.33** | - |
| NM_020247 | CABC1 |  | **1.33** | - |
| NM_000688 | ALAS1 |  | **1.33** | - |
| NM_024038 | C19orf43 |  | **1.32** | - |
| AL122109 | - |  | **1.32** | - |
| NM_145686 | MAP4K4 |  | **1.32** | - |
| NM_016352 | CPA4 |  | **1.32** | - |
| NM_018950 | HLA-F |  | **1.32** | - |
| NM_001022 | RPS19 |  | **1.32** | - |
| NM_024629 | MLF1IP |  | **1.32** | - |
| NM_022839 | MRPS11 |  | **1.32** | - |
| NM_017722 | TRMT1 |  | **1.32** | - |
| NM_012210 | TRIM32 |  | **1.32** | - |
| NM_000224 | KRT18 |  | **1.32** | - |
| NM_025165 | ELL3 |  | **1.32** | - |
| AL137382 | - |  | **1.32** | - |
| AF231919 | - |  | **1.31** | - |
| NM_032560 | KIAA2010 |  | **1.31** | - |
| NM_194281 | C18orf37 |  | **1.31** | - |
| AB017116 | - |  | **1.31** | - |
| NM_023008 | FLJ12949 |  | **1.31** | - |
| NM_019052 | CCHCR1 |  | **1.31** | - |
| NM_033520 | C19orf33 |  | **1.31** | - |
| NM_004231 | ATP6V1F |  | **1.31** | - |
| NM_022827 | SPATA20 |  | **1.31** | - |
| NM_016062 | FAM96B |  | **1.31** | - |
| NM_016538 | SIRT7 |  | **1.31** | - |
| AK095459 | - |  | **1.31** | - |
| NM_015994 | ATP6V1D |  | **1.31** | - |
| NM_138768 | MYEOV |  | **1.31** | - |
| NM_015518 | - |  | **1.30** | - |
| NM_002650 | PIK4CA |  | **1.30** | - |
| NM_004193 | GBF1 |  | **1.30** | - |
| BC020891 | - |  | **1.30** | - |
| NM_001823 | CKB |  | **1.30** | - |
| NM_015644 | TTLL3 |  | **1.30** | - |
| NM_004603 | STX1A |  | **1.30** | - |
| NM_015094 | HIC2 |  | **1.30** | - |
| NM_001540 | HSPB1 |  | **1.29** | HSPB1 |
| NM_007152 | ZNF195 |  | **1.29** | - |
| AK092921 | - |  | **1.29** | - |
| NM_006435 | IFITM2 |  | **1.29** | - |
| NM_144626 | TMEM125 |  | **1.29** | - |
| AK092751 | LOC286254 |  | **1.29** | - |
| NM_005514 | HLA-B |  | **1.29** | - |
| NM_024667 | VPS37B |  | **1.29** | - |
| NM_015675 | GADD45B |  | **1.29** | - |
| NM_079425 | - |  | **1.29** | - |
| NM_015654 | NAT9 |  | **1.29** | - |
| XM_293276 | - |  | **1.29** | - |
| NM_001493 | GDI1 |  | **1.28** | - |
| NM_024097 | C1orf50 |  | **1.28** | - |
| NM_004927 | MRPL49 |  | **1.28** | - |
| NM_014297 | ETHE1 |  | **1.28** | - |
| U41420 | HLA-C |  | **1.28** | - |
| NM_177939 | - |  | **1.28** | - |
| BC033256 | FAM83H |  | **1.28** | - |
| NM_014463 | LSM3 |  | **1.27** | - |
| NM_006007 | ZA20D2 |  | **1.27** | - |
| NM_015517 | MIZF |  | **1.27** | - |
| NM_014851 | KLHL21 |  | **1.27** | - |
| NM_006001 | - |  | **1.27** | - |
| NM_145294 | - |  | **1.27** | - |
| NM_014623 | MEA1 |  | **1.27** | - |
| NM_178191 | ATPIF1 |  | **1.27** | - |
| S81524 | - |  | **1.27** | - |
| NM_016470 | C20orf111 |  | **1.27** | - |
| AK095847 | - |  | **1.27** | - |
| AK122734 | - |  | **1.27** | - |
| NM_000636 | SOD2 |  | **1.27** | SOD2 |
| NM_004594 | SLC9A5 |  | **1.26** | - |
| NM_145249 | - |  | **1.26** | - |
| NM_005112 | - |  | **1.26** | - |
| NM_007065 | CDC37 |  | **1.26** | - |
| BC017996 | - |  | **1.26** | - |
| NM_175609 | ARFGAP1 |  | **1.26** | - |
| NM_004879 | EI24 |  | **1.26** | EI24 |
| NM_000178 | GSS |  | **1.25** | - |
| NM_003429 | ZNF85 |  | **1.25** | - |
| NM_176880 | TRA16 |  | **1.25** | - |
| NM_006696 | BRD8 |  | **1.25** | - |
| NM_024094 | DCC1 |  | **1.25** | - |
| NM_025204 | RP3-402G11.12 | | **1.25** | - |
| AB002313 | PLXNB2 |  | **1.25** | PLXNB2 |
| NM_182687 | PKMYT1 |  | **1.25** | - |
| NM_052960 | RBP7 |  | **1.24** | - |
| NM_002896 | RBM4 |  | **1.24** | - |
| NM_006833 | COPS6 |  | **1.24** | - |
| NM_004328 | BCS1L |  | **1.24** | - |
| NM_181531 | BTN2A2 |  | **1.24** | - |
| NM_006460 | HEXIM1 |  | **1.23** | - |
| NM_016175 | LOC51149 |  | **1.23** | - |
| NM_018447 | TMEM111 |  | **1.23** | - |
| NM_153256 | C10orf47 |  | **1.23** | - |
| NM_004909 | CSAG2 |  | **1.22** | - |
| NM_007294 | - |  | **1.22** | - |
| AK027211 | - |  | **1.22** | - |
| NM_001404 | EEF1G |  | **1.22** | - |
| NM_145201 | NAPRT1 |  | **1.21** | - |
| NM_005441 | CHAF1B |  | **1.21** | - |
| NM_003086 | SNAPC4 |  | **1.21** | - |
| NM_001813 | CENPE |  | **-1.19** | - |
| NM_018084 | KIAA1212 |  | **-1.21** | - |
| NM_005898 | GPIAP1 |  | **-1.21** | - |
| NM_152828 | SNX3 |  | **-1.21** | - |
| NM_018983 | NOLA1 |  | **-1.21** | - |
| AK023152 | - |  | **-1.22** | - |
| NM_181886 | UBE2D3 |  | **-1.23** | - |
| NM_002719 | PPP2R5C |  | **-1.23** | - |
| NM_005500 | SAE1 |  | **-1.23** | - |
| NM_080596 | HIST1H2AH |  | **-1.23** | - |
| CR595813 | - |  | **-1.23** | - |
| NM_145808 | MTPN |  | **-1.24** | - |
| NM_144578 | C14orf32 |  | **-1.24** | - |
| NM_018087 | TMEM48 |  | **-1.24** | - |
| NM_018352 | FLJ11184 |  | **-1.25** | - |
| NM_006406 | PRDX4 |  | **-1.25** | - |
| NM_000179 | MSH6 |  | **-1.25** | - |
| NM_138393 | REEP6 |  | **-1.25** | - |
| NM_000108 | DLD |  | **-1.25** | - |
| NM_053004 | GNB1L |  | **-1.25** | - |
| BC063888 | - |  | **-1.25** | - |
| NM_015960 | CUTC |  | **-1.25** | - |
| NM_031157 | HNRPA1 |  | **-1.25** | - |
| BC010266 | HNRPA1 |  | **-1.25** | - |
| NM_006756 | TCEA1 |  | **-1.25** | - |
| NM_016422 | RNF141 |  | **-1.26** | - |
| NM_207368 | LOC348262 |  | **-1.26** | - |
| NM_013943 | CLIC4 |  | **-1.26** | CLIC4 |
| NM_005001 | NDUFA7 |  | **-1.26** | - |
| NM_205842 | NCKAP1 |  | **-1.27** | - |
| NM_007208 | MRPL3 |  | **-1.27** | - |
| NM_018956 | C9orf9 |  | **-1.27** | - |
| NM_000026 | ADSL |  | **-1.27** | - |
| NM_002906 | RDX |  | **-1.27** | - |
| NM_014017 | MAPBPIP |  | **-1.27** | - |
| NM_002375 | MAP4 |  | **-1.28** | - |
| NM_004453 | ETFDH |  | **-1.28** | - |
| NM_004125 | LOC552891 |  | **-1.28** | - |
| NM_175617 | MT1E |  | **-1.28** | - |
| NM_019063 | EML4 |  | **-1.28** | - |
| NM_016097 | IER3IP1 |  | **-1.28** | - |
| NM_006807 | CBX1 |  | **-1.28** | - |
| XM_062025 | - |  | **-1.28** | - |
| NM_007270 | FKBP9 |  | **-1.29** | - |
| CR593246 | - |  | **-1.29** | - |
| AL117568 | - |  | **-1.29** | - |
| NM_001419 | ELAVL1 |  | **-1.29** | - |
| NM_002808 | PSMD2 |  | **-1.30** | - |
| NM_182903 | KIF9 |  | **-1.30** | - |
| AK002014 | - |  | **-1.30** | - |
| NM_002402 | MEST |  | **-1.30** | - |
| NM_025158 | RUFY1 |  | **-1.30** | - |
| NM_203380 | ACSL5 |  | **-1.30** | - |
| NM_006388 | HTATIP |  | **-1.30** | - |
| NM_017917 | C14orf10 |  | **-1.31** | - |
| NM_018048 | FLJ10292 |  | **-1.31** | - |
| XM_372996 | LOC391555 |  | **-1.31** | - |
| NM_145303 | LOC202459 |  | **-1.31** | - |
| XM_296117 | - |  | **-1.31** | - |
| NM_004607 | TBCA |  | **-1.32** | - |
| BG719281 | - |  | **-1.32** | - |
| NM_002528 | NTHL1 |  | **-1.32** | - |
| NM_032549 | IMMP2L |  | **-1.32** | - |
| NM_006827 | TMED10 |  | **-1.33** | - |
| NM_018361 | AGPAT5 |  | **-1.33** | - |
| NM_006330 | LYPLA1 |  | **-1.33** | - |
| NM_015957 | APIP |  | **-1.33** | - |
| NM_145040 | PRKCDBP |  | **-1.33** | - |
| NM_002136 | HNRPA1 |  | **-1.33** | - |
| NM_018024 | C8orf32 |  | **-1.33** | - |
| NM_001979 | EPHX2 |  | **-1.33** | - |
| NM_024604 | FLJ21908 |  | **-1.33** | - |
| NM_020536 | CSRP2BP |  | **-1.33** | - |
| NM_016108 | AIG1 |  | **-1.33** | - |
| NM_153344 | C6orf141 |  | **-1.34** | - |
| BC062731 | - |  | **-1.34** | - |
| NM_014026 | DCPS |  | **-1.34** | - |
| NM_018394 | ABHD10 |  | **-1.34** | - |
| NM_014254 | TMEM5 |  | **-1.34** | - |
| NM_139076 | CCDC98 |  | **-1.34** | - |
| NM_182760 | SUMF1 |  | **-1.35** | - |
| NM_016059 | PPIL1 |  | **-1.35** | - |
| NM_017905 | TMCO3 |  | **-1.35** | - |
| NM_002709 | PPP1CB |  | **-1.35** | - |
| NM_024834 | C10orf119 |  | **-1.35** | - |
| NM_005327 | HADHSC |  | **-1.35** | - |
| NM_194247 | HNRPA3 |  | **-1.35** | - |
| NM_005869 | SDCCAG10 |  | **-1.35** | - |
| NM_199342 | CCDC23 |  | **-1.35** | - |
| NM_005100 | - |  | **-1.36** | - |
| BX537987 | TRIM59 |  | **-1.36** | - |
| NM_004134 | HSPA9B |  | **-1.36** | - |
| NM_138436 | C8orf40 |  | **-1.36** | - |
| NM_018434 | RNF130 |  | **-1.36** | - |
| NM_006055 | LANCL1 |  | **-1.36** | - |
| NM_032466 | ASPH |  | **-1.36** | - |
| NM_003129 | SQLE |  | **-1.37** | - |
| CR603865 | - |  | **-1.38** | - |
| NM_032208 | ANTXR1 |  | **-1.38** | - |
| NM_014857 | RABGAP1L |  | **-1.38** | - |
| NM_032947 | MST150 |  | **-1.39** | - |
| NM_152726 | EFHA1 |  | **-1.39** | - |
| BC033829 | - |  | **-1.40** | - |
| AJ420574 | - |  | **-1.40** | - |
| NM_013322 | SNX10 |  | **-1.40** | - |
| NM_032188 | MYST1 |  | **-1.40** | - |
| NM_020946 | DENND1A |  | **-1.41** | - |
| NM_014641 | MDC1 |  | **-1.41** | - |
| AB037851 | KIAA1430 |  | **-1.41** | - |
| NM_002485 | NBN |  | **-1.41** | - |
| NM_016315 | GULP1 |  | **-1.41** | - |
| NM_032026 | TATDN1 |  | **-1.41** | - |
| NM_004176 | - |  | **-1.41** | - |
| NM_006324 | CFDP1 |  | **-1.42** | - |
| NM_033360 | KRAS |  | **-1.42** | - |
| AK098081 | - |  | **-1.42** | - |
| NM_006364 | SEC23A |  | **-1.42** | - |
| NM_001453 | FOXC1 |  | **-1.43** | - |
| AI792678 | - |  | **-1.43** | - |
| AK074291 | - |  | **-1.44** | - |
| NM_000245 | MET |  | **-1.44** | - |
| NM_001498 | GCLC |  | **-1.44** | - |
| NM_018374 | TMEM106B |  | **-1.45** | - |
| NM_058179 | PSAT1 |  | **-1.46** | - |
| BC071732 | PTMA |  | **-1.46** | - |
| BG178211 | - |  | **-1.46** | - |
| NM_152524 | SGOL2 |  | **-1.47** | - |
| NM_022740 | HIPK2 |  | **-1.47** | - |
| NM_212554 | LOC399818 |  | **-1.47** | - |
| AF155662 | - |  | **-1.47** | - |
| AK021858 | - |  | **-1.47** | - |
| NM_018076 | ARMC4 |  | **-1.48** | - |
| NM_019061 | MTMR12 |  | **-1.48** | - |
| NM_024420 | PLA2G4A |  | **-1.48** | - |
| BC009038 | - |  | **-1.49** | - |
| AK021751 | - |  | **-1.50** | - |
| NM_031466 | NIBP |  | **-1.50** | - |
| NM_018660 | ZNF395 |  | **-1.50** | - |
| NM_002073 | GNAZ |  | **-1.50** | - |
| NM_182314 | COVA1 |  | **-1.51** | - |
| NM_016270 | KLF2 |  | **-1.53** | - |
| NM_080653 | ATP6V1E2 |  | **-1.53** | - |
| NM_005114 | HS3ST1 |  | **-1.53** | HS3ST1 |
| NM_004753 | DHRS3 |  | **-1.53** | - |
| NM_003325 | HIRA |  | **-1.54** | - |
| NM_003045 | SLC7A1 |  | **-1.54** | - |
| NM_002737 | PRKCA |  | **-1.54** | PRKCA |
| NM_016056 | TMBIM4 |  | **-1.54** | - |
| NM_144497 | AKAP12 |  | **-1.54** | AKAP12 |
| N21624 | YWHAE |  | **-1.54** | - |
| NM_175863 | ARID1B |  | **-1.55** | - |
| CR598364 | - |  | **-1.55** | - |
| NM_006047 | RBM12 |  | **-1.57** | - |
| NM_022743 | SMYD3 |  | **-1.58** | - |
| NM_015440 | MTHFD1L |  | **-1.58** | - |
| NM_153208 | IQCK |  | **-1.59** | - |
| NM_001605 | AARS |  | **-1.61** | - |
| NM_012193 | FZD4 |  | **-1.61** | - |
| NM_003680 | YARS |  | **-1.61** | - |
| NM_006391 | IPO7 |  | **-1.62** | - |
| NM_021158 | TRIB3 |  | **-1.62** | - |
| NM_032042 | C5orf21 |  | **-1.63** | - |
| NM_024060 | AHNAK |  | **-1.64** | - |
| NM_001964 | EGR1 |  | **-1.66** | - |
| NM_004563 | PCK2 |  | **-1.66** | - |
| NM_052965 | C1orf19 |  | **-1.67** | - |
| NM_002047 | GARS |  | **-1.67** | - |
| NM_145244 | DDIT4L |  | **-1.69** | - |
| NM_015508 | TIPARP |  | **-1.72** | - |
| NM_003104 | SORD |  | **-1.72** | - |
| AK091132 | - |  | **-1.73** | - |
| NM_006903 | - |  | **-1.73** | - |
| NM_033402 | LRRCC1 |  | **-1.74** | - |
| NM_032438 | L3MBTL3 |  | **-1.75** | - |
| NM_001010971 | SAMD13 |  | **-1.75** | - |
| NM_033515 | ARHGAP18 |  | **-1.77** | - |
| NM_173576 | MKX |  | **-1.77** | - |
| AF130080 | - |  | **-1.79** | - |
| NM_012331 | MSRA |  | **-1.85** | - |
| NM_018291 | FLJ10986 |  | **-1.91** | - |
| BF210146 | - |  | **-1.93** | - |
| NM_022126 | LHPP |  | **-1.96** | - |
| NM_004457 | ACSL3 |  | **-1.98** | ACSL3 |
| NM_033251 | RPL13 |  | **-2.03** | - |
| NM_000218 | KCNQ1 |  | **-2.07** | - |
| NM_002952 | RPS2 |  | **-2.11** | - |
| AB033060 | AHRR |  | **-2.14** | - |
| BX538293 | - |  | **-2.17** | - |
| NM_005581 | BCAM |  | **-2.22** | - |
| CR627234 | - |  | **-2.27** | - |
| NM_016144 | COMMD10 |  | **-2.27** | - |
|  |  |  |  |  |
|  |  |  |  |  |
| **Cisplatin Common** | | | | |
|  |  |  |  |  |
| **Accession** | **Symbol** | **FoldChangeT** | **FoldChangeD** | **p53 regulated** |
|  |  |  |  |  |
| NM_004881 | TP53I3 | **6.64** | **4.95** | TP53I3 |
| NM_000952 | PTAFR | **5.67** | **4.91** | - |
| NM_004864 | GDF15 | **2.84** | **3.12** | GDF15 |
| NM_018837 | SULF2 | **3.68** | **3.05** | - |
| NM_000389 | CDKN1A | **4.02** | **2.95** | - |
| NM_139314 | - | **2.10** | **2.95** | - |
| NM_017957 | EPN3 | **2.62** | **2.81** | - |
| NM_022772 | EPS8L2 | **3.17** | **2.79** | - |
| NM_078467 | CDKN1A | **4.11** | **2.79** | - |
| AK023754 | - | **2.69** | **2.75** | - |
| NM_182507 | LOC144501 | **2.63** | **2.75** | - |
| NM_004024 | ATF3 | **3.26** | **2.71** | ATF3 |
| NM_004073 | PLK3 | **3.17** | **2.66** | - |
| NM_002639 | SERPINB5 | **3.04** | **2.64** | SERPINB5 |
| NM_020299 | AKR1B10 | **3.63** | **2.56** | - |
| NM_000107 | DDB2 | **3.14** | **2.50** | DDB2 |
| NM_001450 | - | **2.44** | **2.45** | - |
| NM_000835 | GRIN2C | **2.88** | **2.44** | - |
| NM_000227 | LAMA3 | **2.99** | **2.43** | - |
| NM_031308 | EPPK1 | **2.51** | **2.43** | - |
| BC006795 | MGC5370 | **2.93** | **2.42** | - |
| NM_006142 | SFN | **2.98** | **2.38** | SFN |
| NM_004110 | - | **2.90** | **2.36** | - |
| NM_016606 | REEP2 | **1.94** | **2.36** | - |
| BC062776 | - | **2.57** | **2.30** | - |
| NM_007021 | C10orf10 | **2.09** | **2.26** | - |
| NM_032943 | SYTL2 | **1.96** | **2.23** | - |
| NM_198545 | C1orf187 | **2.33** | **2.23** | - |
| NM_014278 | HSPA4L | **2.21** | **2.23** | - |
| NM_001873 | CPE | **1.85** | **2.20** | - |
| NM_001613 | ACTA2 | **2.36** | **2.20** | ACTA2 |
| AK056245 | - | **2.23** | **2.17** | - |
| NM_000228 | - | **2.08** | **2.15** | - |
| NM_004750 | CRLF1 | **2.01** | **2.14** | - |
| NM_006504 | PTPRE | **2.00** | **2.12** | - |
| NM_021615 | CHST6 | **1.93** | **2.10** | - |
| NM_013376 | SERTAD1 | **2.05** | **2.10** | - |
| NM_005101 | ISG15 | **2.11** | **2.07** | - |
| NM_004031 | IRF7 | **1.85** | **2.06** | - |
| AK025631 | - | **2.50** | **2.05** | - |
| NM_152426 | APOBEC3D | **2.23** | **2.03** | - |
| NM_002228 | JUN | **2.03** | **2.02** | - |
| NM_001670 | ARVCF | **2.07** | **2.00** | - |
| AK024898 | - | **2.14** | **2.00** | - |
| NM_018370 | FLJ11259 | **1.99** | **1.99** | - |
| NM_023915 | GPR87 | **2.66** | **1.98** | - |
| NM_004561 | OVOL1 | **1.47** | **1.97** | - |
| NM_201612 | IKIP | **2.11** | **1.96** | - |
| NM_007110 | TEP1 | **1.93** | **1.95** | - |
| NM_005682 | - | **1.95** | **1.94** | - |
| NM_006622 | PLK2 | **3.40** | **1.94** | PLK2 |
| NM_001853 | COL9A3 | **2.22** | **1.93** | - |
| NM_023039 | ANKRA2 | **1.99** | **1.92** | - |
| NM_001856 | COL16A1 | **1.82** | **1.92** | - |
| NM_001657 | AREG | **1.62** | **1.91** | - |
| NM_006516 | SLC2A1 | **1.60** | **1.91** | SLC2A1 |
| NM_003620 | PPM1D | **2.29** | **1.89** | PPM1D |
| NM_001086 | AADAC | **2.45** | **1.87** | - |
| NM_016399 | TRIAP1 | **2.32** | **1.86** | - |
| NM_001924 | GADD45A | **2.11** | **1.86** | GADD45A |
| NM_004419 | DUSP5 | **2.02** | **1.85** | DUSP5 |
| NM_002125 | HLA-DRB5 | **1.81** | **1.85** | - |
| NM_005971 | FXYD3 | **1.93** | **1.83** | FXYD3 |
| NM_004900 | APOBEC3B | **2.02** | **1.83** | - |
| NM_001009991 | SYTL3 | **1.90** | **1.82** | - |
| NM_021732 | AVPI1 | **1.76** | **1.82** | - |
| NM_004148 | NINJ1 | **1.79** | **1.82** | - |
| NM_016201 | AMOTL2 | **1.89** | **1.81** | - |
| XM_291270 | - | **1.94** | **1.81** | - |
| NM_006806 | BTG3 | **1.90** | **1.80** | - |
| NM_001430 | EPAS1 | **1.46** | **1.78** | - |
| NM_004760 | STK17A | **1.97** | **1.78** | - |
| NM_032324 | C1orf57 | **1.73** | **1.77** | - |
| NM_021137 | TNFAIP1 | **1.61** | **1.77** | - |
| NM_005978 | S100A2 | **2.66** | **1.77** | - |
| BC041772 | - | **1.58** | **1.76** | - |
| NM_152392 | AHSA2 | **1.99** | **1.76** | - |
| XM_290516 | - | **2.02** | **1.76** | - |
| NM_003842 | TNFRSF10B | **2.05** | **1.75** | TNFRSF10B |
| AW972815 | - | **1.97** | **1.75** | - |
| NM_032463 | LAT2 | **1.74** | **1.75** | - |
| NM_004636 | SEMA3B | **2.04** | **1.74** | - |
| NM_006404 | PROCR | **1.81** | **1.73** | - |
| S73202 | - | **1.63** | **1.73** | - |
| AB058761 | ZNF469 | **1.78** | **1.73** | - |
| NM_006426 | DPYSL4 | **2.15** | **1.73** | - |
| BC001973 | - | **1.62** | **1.72** | - |
| NM_002201 | ISG20 | **1.64** | **1.71** | - |
| NM_032510 | PARD6G | **1.62** | **1.71** | - |
| NM_016423 | ZNF219 | **1.89** | **1.71** | - |
| NM_001004431 | METRNL | **1.60** | **1.71** | - |
| BE378852 | - | **2.16** | **1.70** | - |
| NM_021127 | PMAIP1 | **2.15** | **1.70** | PMAIP1 |
| BC007947 | - | **1.75** | **1.70** | - |
| NM_015713 | RRM2B | **1.62** | **1.69** | RRM2B |
| AL832534 | - | **1.62** | **1.69** | - |
| NM_014454 | SESN1 | **2.14** | **1.69** | SESN1 |
| NM_015920 | RPS27L | **2.44** | **1.68** | - |
| NM_001311 | CRIP1 | **2.01** | **1.67** | - |
| NM_003584 | DUSP11 | **1.59** | **1.67** | - |
| NM_004431 | EPHA2 | **1.62** | **1.66** | EPHA2 |
| NM_003029 | SHC1 | **1.53** | **1.66** | - |
| NM_012242 | DKK1 | **1.50** | **1.66** | DKK1 |
| NM_032102 | SRP46 | **1.78** | **1.65** | - |
| NM_006736 | DNAJB2 | **1.51** | **1.65** | - |
| NM_016545 | IER5 | **1.88** | **1.64** | - |
| NM_145323 | OSBPL3 | **1.94** | **1.64** | - |
| NM_004324 | - | **1.73** | **1.64** | - |
| NM_002135 | NR4A1 | **1.86** | **1.64** | - |
| NM_001719 | BMP7 | **1.58** | **1.63** | - |
| NM_004138 | KRTHA3A | **1.61** | **1.63** | - |
| NM_006187 | OAS3 | **1.86** | **1.62** | - |
| NM_003975 | SH2D2A | **1.81** | **1.62** | - |
| NM_000476 | AK1 | **2.08** | **1.62** | AK1 |
| NM_019556 | MOSPD1 | **1.45** | **1.61** | - |
| NM_000593 | TAP1 | **1.72** | **1.61** | TAP1 |
| NM_024310 | PLEKHF1 | **1.81** | **1.61** | - |
| NM_006663 | PPP1R13L | **1.53** | **1.61** | - |
| NM_000147 | FUCA1 | **1.46** | **1.60** | - |
| NM_032872 | SYTL1 | **1.66** | **1.60** | - |
| NM_002592 | PCNA | **1.86** | **1.60** | PCNA |
| NM_020448 | NPAL3 | **1.45** | **1.60** | - |
| NM_145886 | - | **2.56** | **1.60** | - |
| NM_000581 | - | **1.74** | **1.60** | - |
| NM_031449 | DKFZp761I2123 | **1.45** | **1.59** | - |
| NM_001007139 | - | **1.49** | **1.59** | - |
| CR749856 | LOC388558 | **1.84** | **1.58** | - |
| NM_031885 | BBS2 | **1.36** | **1.58** | - |
| NM_000043 | FAS | **2.06** | **1.58** | FAS |
| BX538238 | MALAT1 | **1.58** | **1.57** | - |
| NM_173809 | - | **1.69** | **1.57** | - |
| NM_001010925 | ANKRD19 | **1.96** | **1.57** | - |
| AB095939 | - | **1.64** | **1.56** | - |
| NM_030912 | TRIM8 | **1.57** | **1.55** | - |
| NM_014994 | MAPKBP1 | **1.53** | **1.55** | - |
| NM_006000 | TUBA1 | **1.71** | **1.55** | - |
| NM_024508 | ZBED2 | **-1.36** | **1.55** | - |
| NM_024101 | MLPH | **1.34** | **1.55** | - |
| NM_022074 | FAM111A | **1.64** | **1.55** | - |
| NM_001333 | CTSL2 | **1.48** | **1.54** | - |
| NM_203302 | MGC70863 | **1.57** | **1.54** | - |
| NM_182919 | TICAM1 | **1.42** | **1.53** | - |
| NM_004472 | FOXD1 | **1.58** | **1.53** | - |
| NM_014301 | NIFUN | **1.58** | **1.53** | - |
| NM_006096 | NDRG1 | **1.35** | **1.53** | NDRG1 |
| NM_004165 | RRAD | **1.65** | **1.53** | - |
| AK125231 | - | **1.65** | **1.53** | - |
| NM_000854 | GSTT2 | **1.63** | **1.52** | - |
| NM_020375 | C12orf5 | **1.84** | **1.52** | - |
| NM_024723 | - | **1.66** | **1.51** | - |
| NM_002751 | MAPK11 | **1.72** | **1.51** | - |
| NM_001878 | CRABP2 | **1.71** | **1.51** | - |
| BC000632 | - | **1.61** | **1.51** | - |
| NM_006698 | BLCAP | **1.63** | **1.50** | - |
| NM_001548 | IFIT1 | **1.36** | **1.50** | - |
| M27126 | - | **1.65** | **1.50** | - |
| NM_080821 | C20orf108 | **1.54** | **1.50** | - |
| NM_022370 | ROBO3 | **1.53** | **1.50** | - |
| NM_001554 | CYR61 | **2.16** | **1.50** | - |
| NM_203394 | E2F7 | **1.93** | **1.50** | - |
| NM_005749 | TOB1 | **1.51** | **1.50** | - |
| NM_003273 | TM7SF2 | **1.43** | **1.50** | - |
| NM_016368 | ISYNA1 | **1.82** | **1.50** | - |
| NM_000930 | PLAT | **1.58** | **1.49** | - |
| NM_001008401 | FLJ16231 | **1.72** | **1.49** | - |
| NM_005485 | - | **1.46** | **1.49** | - |
| NM_018043 | TMEM16A | **1.54** | **1.49** | - |
| NM_003651 | CSDA | **1.45** | **1.48** | - |
| NM_003869 | CES2 | **1.50** | **1.48** | - |
| NM_004925 | AQP3 | **1.58** | **1.48** | - |
| NM_014172 | PHPT1 | **1.63** | **1.47** | - |
| NM_033245 | PML | **1.79** | **1.47** | - |
| AK024303 | - | **1.88** | **1.47** | - |
| AK024480 | - | **1.38** | **1.47** | - |
| NM_014059 | RGC32 | **2.06** | **1.47** | - |
| NM_006266 | RALGDS | **1.41** | **1.47** | - |
| NM_033285 | TP53INP1 | **1.92** | **1.46** | - |
| NM_004993 | ATXN3 | **1.55** | **1.46** | - |
| NM_052901 | - | **1.43** | **1.46** | - |
| NM_004099 | - | **1.47** | **1.46** | - |
| NM_032876 | JUB | **1.50** | **1.45** | - |
| NM_018161 | NADSYN1 | **1.76** | **1.45** | - |
| NM_013390 | TMEM2 | **1.44** | **1.45** | - |
| K03200 | LOC440995 | **1.35** | **1.44** | - |
| NM_031904 | FKSG44 | **1.67** | **1.44** | - |
| NM_015954 | DERA | **1.33** | **1.44** | - |
| AK126845 | - | **1.41** | **1.44** | - |
| BC069216 | - | **1.33** | **1.44** | - |
| NM_148965 | - | **1.57** | **1.43** | - |
| NM_012168 | FBXO2 | **1.47** | **1.43** | - |
| NM_014417 | BBC3 | **1.40** | **1.43** | BBC3 |
| NM_005620 | S100A11 | **1.38** | **1.43** | - |
| AK096811 | - | **1.51** | **1.43** | - |
| NM_198686 | RAB15 | **1.72** | **1.43** | - |
| NM_003761 | VAMP8 | **1.48** | **1.43** | - |
| AB041269 | LOC160313 | **1.42** | **1.42** | - |
| NM_013937 | OR11A1 | **1.52** | **1.42** | - |
| NM_000181 | GUSB | **1.37** | **1.42** | - |
| NM_017527 | LY6K | **3.27** | **1.41** | - |
| NM_016029 | DHRS7 | **1.36** | **1.41** | - |
| NM_025194 | ITPKC | **1.38** | **1.41** | - |
| AK091153 | - | **1.29** | **1.41** | - |
| NM_004281 | BAG3 | **1.33** | **1.40** | - |
| NM_152640 | DCP1B | **1.42** | **1.40** | - |
| NM_004747 | DLG5 | **1.41** | **1.40** | - |
| BC040043 | - | **1.54** | **1.40** | - |
| NM_002658 | PLAU | **1.31** | **1.39** | - |
| NM_052871 | MGC4677 | **1.56** | **1.39** | - |
| NM_004999 | MYO6 | **1.30** | **1.39** | - |
| NM_198446 | C1orf122 | **1.48** | **1.38** | - |
| NM_032034 | SLC4A11 | **1.60** | **1.38** | - |
| NM_002386 | MC1R | **1.28** | **1.38** | - |
| NM_003992 | CLK3 | **1.40** | **1.38** | - |
| NM_001153 | ANXA4 | **1.30** | **1.38** | - |
| NM_006831 | HEAB | **1.44** | **1.38** | - |
| NM_021825 | MDS025 | **1.47** | **1.37** | - |
| NM_002307 | LGALS7 | **1.63** | **1.37** | - |
| NM_032525 | TUBB6 | **1.36** | **1.37** | - |
| AF086126 | - | **1.41** | **1.37** | - |
| NM_020418 | - | **1.54** | **1.37** | - |
| NM_002818 | PSME2 | **1.32** | **1.36** | - |
| BC014776 | - | **1.57** | **1.36** | - |
| NM_172199 | - | **1.48** | **1.36** | - |
| XM_371762 | - | **1.33** | **1.36** | - |
| NM_015246 | MGRN1 | **1.40** | **1.36** | - |
| NM_021077 | NMB | **1.40** | **1.36** | - |
| NM_021173 | POLD4 | **1.32** | **1.36** | - |
| NM_005415 | SLC20A1 | **1.35** | **1.35** | - |
| NM_021005 | NR2F2 | **1.27** | **1.35** | - |
| NM_174905 | FAM98C | **1.69** | **1.35** | - |
| NM_003449 | TRIM26 | **1.46** | **1.34** | - |
| NM_005689 | ABCB6 | **1.53** | **1.34** | - |
| NM_001747 | CAPG | **1.30** | **1.34** | - |
| NM_022343 | C9orf19 | **1.27** | **1.34** | - |
| NM_153337 | - | **1.44** | **1.34** | - |
| NM_002134 | HMOX2 | **1.36** | **1.34** | - |
| NM_000169 | GLA | **1.45** | **1.33** | - |
| NM_006509 | RELB | **1.21** | **1.33** | - |
| AL117478 | - | **1.28** | **1.33** | - |
| NM_198457 | ZNF600 | **1.58** | **1.33** | - |
| NM_001712 | CEACAM1 | **1.62** | **1.33** | - |
| NM_001001433 | STX16 | **1.39** | **1.33** | - |
| NM_014736 | KIAA0101 | **1.65** | **1.32** | - |
| U14391 | MYO1E | **1.47** | **1.32** | - |
| NM_018193 | KIAA1794 | **1.54** | **1.32** | - |
| NM_007275 | TUSC2 | **1.43** | **1.32** | - |
| NM_024041 | - | **1.38** | **1.31** | - |
| NM_001800 | CDKN2D | **1.41** | **1.30** | CDKN2D |
| AK092260 | DUSP5P | **1.93** | **1.30** | - |
| NM_006802 | SF3A3 | **1.42** | **1.29** | - |
| BC000845 | - | **1.57** | **1.27** | - |
| NM_017491 | WDR1 | **1.25** | **1.27** | - |
| NM_003686 | EXO1 | **1.49** | **1.27** | - |
| M21963 | HLA-C | **1.29** | **1.27** | - |
| NM_020947 | KIAA1609 | **1.29** | **1.27** | - |
| NM_016551 | TM7SF3 | **1.42** | **1.27** | - |
| NM_001625 | AK2 | **1.38** | **1.27** | - |
| XM_498018 | - | **1.33** | **1.27** | - |
| NM_002117 | HLA-C | **1.29** | **1.27** | - |
| NM_001054 | - | **1.37** | **1.27** | - |
| NM_133491 | SAT2 | **1.29** | **1.26** | - |
| NM_152318 | C12orf45 | **1.43** | **1.26** | - |
| NM_002133 | HMOX1 | **1.26** | **1.24** | - |
| NM_015996 | SIDT2 | **1.24** | **1.24** | - |
| BC010117 | - | **1.31** | **1.23** | - |
| NM_001107 | - | **1.38** | **1.23** | - |
| NM_002493 | - | **-1.28** | **-1.21** | - |
| NM_002807 | PSMD1 | **-1.32** | **-1.23** | - |
| NM_002296 | LBR | **-1.50** | **-1.25** | - |
| NM_003816 | ADAM9 | **-1.48** | **-1.25** | - |
| NM_006793 | PRDX3 | **-1.27** | **-1.26** | - |
| NM_012201 | GLG1 | **-1.34** | **-1.27** | - |
| NM_172251 | MRPL54 | **-1.26** | **-1.27** | - |
| NM_007350 | PHLDA1 | **-1.41** | **-1.27** | PHLDA1 |
| NM_007273 | PHB2 | **-1.26** | **-1.28** | - |
| NM_001358 | DHX15 | **-1.31** | **-1.28** | - |
| NM_032860 | LTV1 | **-1.39** | **-1.29** | - |
| NM_000904 | NQO2 | **-1.39** | **-1.29** | - |
| NM_004642 | CDK2AP1 | **-1.26** | **-1.29** | - |
| NM_138369 | FAM44B | **-1.43** | **-1.31** | - |
| BE898801 | - | **-1.44** | **-1.31** | - |
| NM_001469 | XRCC6 | **-1.34** | **-1.31** | - |
| NM_001875 | CPS1 | **-1.56** | **-1.32** | - |
| NM_012207 | HNRPH3 | **-1.22** | **-1.32** | - |
| NM_139177 | SLC39A11 | **-1.68** | **-1.32** | - |
| NM_001675 | ATF4 | **-1.34** | **-1.32** | ATF4 |
| NM_139207 | NAP1L1 | **-1.45** | **-1.33** | - |
| XM_496593 | - | **-1.26** | **-1.33** | - |
| NM_012227 | GTPBP6 | **-1.37** | **-1.33** | - |
| NM_000304 | PMP22 | **-1.26** | **-1.33** | - |
| NM_032814 | TMEM118 | **-1.44** | **-1.33** | - |
| NM_004990 | MARS | **-1.66** | **-1.33** | - |
| NM_006993 | NPM3 | **-1.36** | **-1.34** | - |
| NM_001827 | CKS2 | **-1.36** | **-1.34** | - |
| NM_031989 | PCBP2 | **-1.50** | **-1.34** | - |
| CR616535 | - | **-1.38** | **-1.35** | - |
| NM_005038 | PPID | **-1.29** | **-1.35** | - |
| NM_003662 | PIR | **-1.59** | **-1.35** | - |
| NM_032730 | RTN4IP1 | **-1.48** | **-1.35** | - |
| NM_013238 | DNAJC15 | **-1.27** | **-1.35** | - |
| NM_004559 | YBX1 | **-1.22** | **-1.35** | - |
| NM_002128 | HMGB1 | **-1.31** | **-1.35** | - |
| NM_176870 | MT1M | **-1.46** | **-1.36** | - |
| XM_047355 | DCAMKL3 | **-1.33** | **-1.37** | - |
| BC009510 | - | **-1.24** | **-1.37** | - |
| NM_021198 | CTDSP1 | **-1.41** | **-1.37** | - |
| NM_002208 | ITGAE | **-1.33** | **-1.38** | - |
| NM_014412 | CACYBP | **-1.33** | **-1.38** | - |
| NM_001280 | CIRBP | **-1.39** | **-1.39** | - |
| NM_032565 | EBPL | **-1.48** | **-1.39** | - |
| AB020684 | - | **-1.41** | **-1.39** | - |
| NM_003113 | SP100 | **1.46** | **-1.40** | - |
| BC045572 | - | **-1.44** | **-1.40** | - |
| NM_002823 | PTMA | **-1.54** | **-1.40** | - |
| CR595483 | - | **-1.43** | **-1.41** | - |
| XM_497271 | HMGB1 | **-1.39** | **-1.41** | - |
| NM_001168 | - | **-1.31** | **-1.41** | - |
| NM_003011 | SET | **-1.46** | **-1.41** | - |
| XM_498178 | - | **-1.48** | **-1.42** | - |
| BQ317309 | - | **-1.48** | **-1.42** | - |
| NM_007235 | XPOT | **-1.49** | **-1.42** | - |
| NM_001004419 | CLEC2D | **-1.41** | **-1.42** | - |
| NM_002520 | NPM1 | **-1.45** | **-1.42** | - |
| NM_017867 | C4orf27 | **-1.47** | **-1.42** | - |
| NM_004516 | ILF3 | **-1.42** | **-1.43** | - |
| NM_005915 | MCM6 | **-1.23** | **-1.43** | - |
| NM_199185 | NPM1 | **-1.49** | **-1.44** | - |
| NM_005381 | NCL | **-1.27** | **-1.44** | - |
| NM_000097 | CPOX | **-1.70** | **-1.44** | CPOX |
| NM_145729 | MRPL24 | **-1.39** | **-1.44** | - |
| NM_001533 | HNRPL | **-1.30** | **-1.45** | - |
| NM_006401 | ANP32B | **-1.49** | **-1.45** | - |
| NM_153824 | PYCR1 | **-1.59** | **-1.45** | - |
| NM_003017 | SFRS3 | **-1.51** | **-1.45** | SFRS3 |
| AY007110 | - | **-1.38** | **-1.45** | - |
| NM_017425 | SPA17 | **-1.59** | **-1.45** | - |
| NM_207578 | PRKACB | **-1.77** | **-1.46** | - |
| NM_030920 | ANP32E | **-1.48** | **-1.46** | - |
| NM_080820 | HARS2 | **-1.66** | **-1.47** | - |
| XM_496355 | - | **-1.48** | **-1.47** | - |
| NM_003517 | HIST2H2AC | **-1.90** | **-1.47** | - |
| NM_014750 | DLG7 | **-1.52** | **-1.48** | - |
| NM_006739 | MCM5 | **-1.26** | **-1.48** | - |
| AF170294 | PTMAP7 | **-1.52** | **-1.48** | - |
| NM_002156 | HSPD1 | **-1.45** | **-1.48** | - |
| NM_005063 | SCD | **-1.74** | **-1.48** | - |
| AK092810 | - | **-1.64** | **-1.49** | - |
| NM_004483 | GCSH | **-1.64** | **-1.49** | - |
| CR616772 | HSP90AB1 | **-1.59** | **-1.50** | HSP90AB1 |
| NM_145269 | - | **-1.39** | **-1.50** | - |
| NM_003486 | SLC7A5 | **-1.77** | **-1.50** | - |
| NM_005826 | HNRPR | **-1.34** | **-1.50** | - |
| NM_005517 | HMGN2 | **-1.25** | **-1.50** | HMGN2 |
| NM_005602 | CLDN11 | **-1.60** | **-1.50** | - |
| NM_206808 | CLYBL | **-1.99** | **-1.52** | - |
| NM_007355 | HSP90AB1 | **-1.62** | **-1.52** | HSP90AB1 |
| AF132203 | SCD | **-1.84** | **-1.52** | - |
| NM_002137 | HNRPA2B1 | **-1.44** | **-1.52** | - |
| NM_033429 | CALML4 | **-1.61** | **-1.53** | - |
| NM_002106 | H2AFZ | **-1.42** | **-1.54** | - |
| NM_001432 | EREG | **-1.94** | **-1.54** | - |
| NM_003600 | - | **-1.42** | **-1.54** | - |
| NM_080927 | DCBLD2 | **-1.60** | **-1.55** | - |
| NM_006743 | - | **-1.59** | **-1.56** | - |
| NM_000903 | NQO1 | **-1.64** | **-1.56** | - |
| AF152351 | - | **-1.36** | **-1.56** | - |
| NM_006276 | SFRS7 | **-1.42** | **-1.56** | - |
| NM_005481 | THRAP5 | **-1.56** | **-1.58** | - |
| NM_031370 | HNRPD | **-1.62** | **-1.58** | - |
| NM_016138 | COQ7 | **-1.56** | **-1.58** | - |
| NM_004336 | BUB1 | **-1.75** | **-1.59** | BUB1 |
| NM_006716 | DBF4 | **-1.55** | **-1.59** | - |
| NM_001673 | ASNS | **-2.88** | **-1.59** | - |
| XM_374273 | LOC389669 | **-1.39** | **-1.61** | - |
| NM_003539 | HIST1H4D | **-1.52** | **-1.62** | - |
| NM_002138 | HNRPD | **-1.57** | **-1.62** | - |
| AI015919 | - | **-1.55** | **-1.62** | - |
| NM_031372 | HNRPDL | **-1.59** | **-1.62** | - |
| NM_003544 | HIST1H4B | **-1.55** | **-1.62** | - |
| NM_003546 | HIST1H4L | **-1.53** | **-1.63** | - |
| XM_372040 | - | **-2.93** | **-1.63** | - |
| NM_014595 | NT5C | **-1.42** | **-1.64** | - |
| BC090057 | - | **-1.51** | **-1.65** | - |
| NM_003542 | HIST1H4C | **-1.55** | **-1.65** | - |
| NM_033661 | WDR4 | **-1.77** | **-1.66** | - |
| NM_021066 | HIST1H2AJ | **-1.48** | **-1.66** | - |
| NM_018205 | LRRC20 | **-1.83** | **-1.66** | - |
| NM_013293 | TRA2A | **-1.40** | **-1.66** | - |
| NM_014899 | RHOBTB3 | **-1.54** | **-1.66** | - |
| NM_001970 | EIF5A | **-1.72** | **-1.67** | - |
| NM_031966 | CCNB1 | **-1.84** | **-1.67** | CCNB1 |
| NM_005733 | KIF20A | **-1.69** | **-1.67** | - |
| NM_005321 | HIST1H1E | **-1.43** | **-1.72** | - |
| NM_021009 | UBC | **-2.25** | **-1.73** | - |
| NM_004853 | STX8 | **-2.07** | **-1.74** | - |
| NM_002010 | FGF9 | **-1.84** | **-1.78** | - |
| BC044619 | FLJ42709 | **-1.81** | **-1.78** | - |
| NM_001255 | CDC20 | **-1.69** | **-1.80** | - |
| NM_005794 | - | **-2.00** | **-1.85** | - |
| NM_005654 | NR2F1 | **-1.77** | **-1.85** | - |
| CR626729 | - | **-1.57** | **-1.85** | - |
| NM_080650 | ATPBD4 | **-2.02** | **-1.85** | - |
| NM_012458 | TIMM13 | **-1.59** | **-1.92** | - |
| NM_182908 | DHRS2 | **-2.15** | **-1.96** | - |
| NM_006026 | H1FX | **-1.79** | **-1.99** | - |
| NM_018669 | - | **-2.11** | **-2.13** | - |
| BF511442 | HIST1H2BD | **-2.05** | **-2.16** | - |
| AF108138 | - | **-2.07** | **-2.24** | - |
| BC041925 | SLC7A11 | **-3.44** | **-2.29** | - |
| CA310244 | HIST1H2BN | **-2.12** | **-2.30** | - |
|  |  |  |  |  |
| **Cisplatin Tetraploid specific** | | | | |
|  |  |  |  |  |
| **Accession** | **Symbol** | **FoldChangeT** |  | **p53 regulated** |
|  |  |  |  |  |
| NM_130778 | - | **3.39** |  | - |
| NM_020672 | S100A14 | **1.95** |  | - |
| NM_003004 | SECTM1 | **1.80** |  | - |
| NM_199461 | NANOS1 | **1.79** |  | - |
| NM_173660 | C4orf25 | **1.79** |  | - |
| BC012203 | - | **1.76** |  | - |
| NM_003282 | TNNI2 | **1.73** |  | - |
| NM_002276 | KRT19 | **1.67** |  | - |
| NM_014475 | DHDH | **1.66** |  | - |
| NM_000693 | ALDH1A3 | **1.65** |  | - |
| NM_016357 | LIMA1 | **1.60** |  | - |
| NM_014568 | GALNT5 | **1.58** |  | - |
| NM_006640 | 9-sep | **1.56** |  | - |
| NM_014624 | S100A6 | **1.55** |  | - |
| NM_033380 | COL4A5 | **1.53** |  | - |
| NM_003841 | TNFRSF10C | **1.51** |  | TNFRSF10C |
| NM_003622 | PPFIBP1 | **1.51** |  | - |
| NM_182532 | TMEM61 | **1.50** |  | - |
| AB007877 | HSPA12A | **1.49** |  | - |
| NM_006084 | ISGF3G | **1.42** |  | - |
| NM_000713 | BLVRB | **1.41** |  | - |
| NM_024599 | RHBDF2 | **1.40** |  | - |
| NM_032957 | - | **1.40** |  | - |
| NM_000527 | LDLR | **1.39** |  | - |
| NM_002166 | ID2 | **1.38** |  | ID2 |
| NM_024836 | ZNF672 | **1.38** |  | - |
| NM_005399 | PRKAB2 | **1.37** |  | - |
| NM_002245 | KCNK1 | **1.37** |  | - |
| AK026078 | - | **1.37** |  | - |
| NM_182565 | FAM100B | **1.36** |  | - |
| NM_203305 | FAM102A | **1.36** |  | - |
| NM_020248 | CTNNBIP1 | **1.36** |  | - |
| NM_024928 | OBFC1 | **1.36** |  | - |
| NM_032772 | ZNF503 | **1.35** |  | - |
| NM_014860 | SUPT7L | **1.35** |  | - |
| AK055659 | TSPAN5 | **1.34** |  | - |
| NM_021724 | NR1D1 | **1.34** |  | - |
| NM_000712 | BLVRA | **1.34** |  | - |
| NM_000158 | GBE1 | **1.33** |  | - |
| NM_004161 | RAB1A | **1.32** |  | - |
| NM_152737 | RNF182 | **1.31** |  | - |
| NM_173551 | ANKS6 | **1.31** |  | - |
| NM_002970 | SAT | **1.30** |  | SAT |
| NM_032901 | C12orf62 | **1.30** |  | - |
| NM_018428 | UTP6 | **1.29** |  | - |
| NM_203463 | LASS6 | **1.28** |  | - |
| AK002107 | - | **1.28** |  | - |
| NM_144505 | KLK8 | **1.27** |  | - |
| NM_002087 | GRN | **1.27** |  | - |
| NM_145006 | SUSD3 | **1.26** |  | - |
| NM_030926 | ITM2C | **1.26** |  | - |
| NM_201535 | NDRG2 | **1.26** |  | - |
| NM_000320 | QDPR | **1.25** |  | - |
| NM_153425 | TRADD | **1.25** |  | - |
| NM_031484 | MARVELD1 | **1.25** |  | - |
| NM_052951 | DNTTIP1 | **1.25** |  | - |
| NM_002127 | HLA-G | **1.23** |  | - |
| NM_000311 | PRNP | **1.23** |  | - |
| NM_001001790 | FBXO10 | **-1.22** |  | - |
| BC063642 | PDE4DIP | **-1.23** |  | - |
| NM_017735 | TTC27 | **-1.24** |  | - |
| NM_020062 | SLC2A4RG | **-1.24** |  | - |
| NM_005175 | ATP5G1 | **-1.24** |  | - |
| NM_004541 | NDUFA1 | **-1.25** |  | - |
| NM_144998 | STRA13 | **-1.25** |  | - |
| NM_001614 | ACTG1 | **-1.25** |  | - |
| NM_003510 | HIST1H2AK | **-1.26** |  | - |
| NM_033416 | IMP4 | **-1.26** |  | - |
| NM_016548 | GOLPH2 | **-1.26** |  | - |
| NM_021064 | HIST1H2AG | **-1.27** |  | - |
| AF355801 | CYP3A5 | **-1.27** |  | - |
| NM_032356 | LSMD1 | **-1.27** |  | - |
| NM_018010 | IFT57 | **-1.27** |  | - |
| AK055809 | - | **-1.27** |  | - |
| NM_019610 | RBMXL1 | **-1.27** |  | - |
| NM_001416 | EIF4A1 | **-1.28** |  | - |
| NM_001687 | - | **-1.28** |  | - |
| NM_003146 | SSRP1 | **-1.28** |  | - |
| NM_016404 | HSPC152 | **-1.29** |  | - |
| NM_000189 | HK2 | **-1.29** |  | - |
| NM_006761 | YWHAE | **-1.30** |  | - |
| NM_002129 | HMGB2 | **-1.30** |  | - |
| NM_031844 | HNRPU | **-1.30** |  | - |
| NM_002806 | PSMC6 | **-1.30** |  | - |
| XM_379872 | - | **-1.30** |  | - |
| NM_016095 | GINS2 | **-1.30** |  | - |
| NM_170711 | DAZAP1 | **-1.30** |  | - |
| NM_006452 | PAICS | **-1.31** |  | - |
| NM_016424 | CROP | **-1.31** |  | - |
| NM_005520 | HNRPH1 | **-1.31** |  | - |
| NM_022085 | TXNDC5 | **-1.31** |  | - |
| CR620336 | - | **-1.31** |  | - |
| NM_170746 | C11orf31 | **-1.31** |  | - |
| NM_006392 | NOL5A | **-1.32** |  | - |
| NM_006331 | EMG1 | **-1.32** |  | - |
| AK026132 | - | **-1.32** |  | - |
| AY014272 | - | **-1.32** |  | - |
| NM_033070 | CECR5 | **-1.33** |  | - |
| NM_004595 | SMS | **-1.33** |  | - |
| NM_017838 | NOLA2 | **-1.34** |  | - |
| NM_016458 | C8orf30A | **-1.35** |  | - |
| NM_002139 | RBMX | **-1.35** |  | - |
| NM_005782 | THOC4 | **-1.36** |  | - |
| NM_001444 | FABP5 | **-1.36** |  | - |
| NM_003514 | HIST1H2AM | **-1.36** |  | - |
| NM_004548 | NDUFB10 | **-1.36** |  | - |
| NM_005348 | HSP90AA1 | **-1.36** |  | HSP90AA1 |
| NM_024658 | IPO4 | **-1.37** |  | - |
| NM_003095 | SNRPF | **-1.37** |  | - |
| NM_001536 | - | **-1.37** |  | - |
| NM_032636 | PSRC1 | **-1.37** |  | - |
| NM_006597 | HSPA8 | **-1.37** |  | HSPA8 |
| NM_024516 | C16orf53 | **-1.37** |  | - |
| NM_016587 | CBX3 | **-1.38** |  | - |
| NM_006325 | RAN | **-1.38** |  | - |
| NM_005274 | GNG5 | **-1.38** |  | - |
| NM_016202 | ZNF580 | **-1.38** |  | - |
| NM_001357 | DHX9 | **-1.39** |  | - |
| NM_023936 | MRPS34 | **-1.39** |  | - |
| NM_012124 | CHORDC1 | **-1.40** |  | - |
| NM_003845 | DYRK4 | **-1.41** |  | - |
| NM_001212 | C1QBP | **-1.41** |  | - |
| D28589 | - | **-1.42** |  | - |
| NM_033445 | HIST3H2A | **-1.43** |  | - |
| NM_002819 | PTBP1 | **-1.44** |  | - |
| NM_003385 | VSNL1 | **-1.44** |  | - |
| NM_173609 | - | **-1.46** |  | - |
| AK022713 | C20orf27 | **-1.48** |  | - |
| NM_020810 | TRMT5 | **-1.48** |  | - |
| NM_001101 | ACTB | **-1.49** |  | - |
| XM_379625 | - | **-1.49** |  | - |
| NM_014783 | ARHGAP11A | **-1.50** |  | - |
| NM_032359 | C3orf26 | **-1.50** |  | - |
| NM_003380 | VIM | **-1.51** |  | - |
| NM_002539 | ODC1 | **-1.51** |  | - |
| NM_002157 | HSPE1 | **-1.55** |  | - |
| NM_004309 | ARHGDIA | **-1.55** |  | - |
| NM_178014 | TUBB | **-1.56** |  | - |
| NM_005030 | PLK1 | **-1.58** |  | PLK1 |
| NM_014042 | C11orf51 | **-1.62** |  | - |
| NM_207418 | - | **-1.64** |  | - |
| NM_025049 | C15orf20 | **-2.95** |  | - |

| **C. Modification of gene expression by Cisplatin and Chk1 inhibitor in diploid and tetraploid cells** | | | | |
| --- | --- | --- | --- | --- |
|  |  |  |  |  |
| **Cisplatin + Chk1 inhibitor Diploid specific** | | | | |
|  |  |  |  |  |
| **Accession** | **Symbol** |  | **FoldChangeD** | **p53 regulated** |
|  |  |  |  |  |
| NM_000584 | IL8 |  | 4.91 | - |
| NM_020777 | SORCS2 |  | 3.61 | - |
| NM_007032 | TRIOBP |  | 2.06 | - |
| NM_001165 | BIRC3 |  | 2.02 | - |
| AL117661 | - |  | 1.98 | - |
| NM_002276 | KRT19 |  | 1.93 | - |
| NM_000270 | NP |  | 1.76 | - |
| NM_014314 | DDX58 |  | 1.75 | - |
| NM_014624 | S100A6 |  | 1.73 | - |
| NM_001004301 | FLJ16542 |  | 1.70 | - |
| NM_153687 | IKIP |  | 1.69 | - |
| NM_021980 | - |  | 1.67 | - |
| AK127993 | - |  | 1.66 | - |
| NM_002273 | KRT8 |  | 1.64 | - |
| NM_012118 | CCRN4L |  | 1.62 | - |
| NM_198489 | CCDC84 |  | 1.62 | - |
| NM_176072 | P2RY2 |  | 1.60 | - |
| NM_021818 | SAV1 |  | 1.59 | - |
| AF086126 | - |  | 1.57 | - |
| NM_005501 | - |  | 1.56 | - |
| NM_004699 | FAM50A |  | 1.56 | - |
| AK024303 | - |  | 1.56 | - |
| NM_001671 | ASGR1 |  | 1.56 | - |
| NM_173624 | FLJ40504 |  | 1.55 | - |
| NM_021052 | HIST1H2AE |  | 1.55 | - |
| XM_497978 | - |  | 1.54 | - |
| NM_134270 | - |  | 1.54 | - |
| AF277181 | - |  | 1.54 | - |
| NM_016038 | SBDS |  | 1.53 | - |
| NM_001124 | ADM |  | 1.53 | - |
| NM_004068 | AP2M1 |  | 1.53 | - |
| NM_001831 | - |  | 1.52 | - |
| AF216862 | - |  | 1.52 | - |
| AK097947 | - |  | 1.51 | - |
| NM_022074 | FAM111A |  | 1.50 | - |
| NM_002751 | MAPK11 |  | 1.49 | - |
| NM_080665 | MGC19604 |  | 1.48 | - |
| NM_019848 | SLC10A3 |  | 1.48 | - |
| NM_005402 | RALA |  | 1.48 | - |
| NM_013299 | SAC3D1 |  | 1.48 | - |
| AI423557 | ISG15 |  | 1.47 | - |
| NM_017414 | USP18 |  | 1.47 | - |
| NM_004669 | CLIC3 |  | 1.46 | - |
| NM_014736 | KIAA0101 |  | 1.45 | - |
| NM_013290 | PSMC3IP |  | 1.45 | - |
| NM_001034 | RRM2 |  | 1.44 | - |
| NM_004925 | AQP3 |  | 1.44 | - |
| NM_005112 | - |  | 1.43 | - |
| NM_002018 | FLII |  | 1.42 | - |
| NM_024733 | ZNF665 |  | 1.42 | - |
| NM_001320 | CSNK2B |  | 1.41 | - |
| NM_079425 | - |  | 1.41 | - |
| NM_021065 | HIST1H2AD |  | 1.41 | - |
| NM_001719 | BMP7 |  | 1.41 | - |
| NM_015590 | - |  | 1.40 | - |
| NM_005399 | PRKAB2 |  | 1.40 | - |
| NM_001753 | CAV1 |  | 1.39 | CAV1 |
| XM_293276 | - |  | 1.38 | - |
| NM_001712 | CEACAM1 |  | 1.38 | - |
| NM_203282 | ZNF539 |  | 1.37 | - |
| AY029066 | - |  | 1.37 | - |
| AK122613 | - |  | 1.37 | - |
| NM_032711 | - |  | 1.36 | - |
| NM_001540 | HSPB1 |  | 1.36 | HSPB1 |
| NM_206920 | MAMDC4 |  | 1.36 | - |
| NM_001628 | AKR1B1 |  | 1.35 | - |
| NM_018193 | KIAA1794 |  | 1.35 | - |
| NM_002966 | S100A10 |  | 1.35 | - |
| NM_178191 | ATPIF1 |  | 1.35 | - |
| NM_080430 | SELM |  | 1.34 | - |
| NM_016551 | TM7SF3 |  | 1.34 | - |
| NM_004231 | ATP6V1F |  | 1.34 | - |
| NM_014999 | RAB21 |  | 1.34 | - |
| AK026078 | - |  | 1.33 | - |
| NM_014851 | KLHL21 |  | 1.33 | - |
| BC000845 | - |  | 1.33 | - |
| NM_002307 | LGALS7 |  | 1.33 | - |
| NM_016400 | HYPK |  | 1.33 | - |
| XM_377998 | - |  | 1.33 | - |
| NM_020933 | ZNF317 |  | 1.32 | - |
| NM_000374 | UROD |  | 1.32 | - |
| NM_003768 | PEA15 |  | 1.32 | - |
| NM_024038 | C19orf43 |  | 1.32 | - |
| NM_001107 | - |  | 1.32 | - |
| NM_001323 | CST6 |  | 1.32 | - |
| NM_017503 | SURF2 |  | 1.31 | - |
| NM_002876 | RAD51C |  | 1.31 | - |
| NM_145055 | C18orf25 |  | 1.30 | - |
| BC020891 | - |  | 1.30 | - |
| NM_032731 | TXNL5 |  | 1.30 | - |
| NM_014463 | LSM3 |  | 1.30 | - |
| NM_012170 | FBXO22 |  | 1.30 | - |
| NM_006460 | HEXIM1 |  | 1.30 | - |
| NM_003333 | UBA52 |  | 1.29 | - |
| NM_022343 | C9orf19 |  | 1.29 | - |
| NM_153425 | TRADD |  | 1.29 | - |
| NM_020239 | CDC42SE1 |  | 1.29 | - |
| CR600908 | - |  | 1.29 | - |
| NM_016538 | SIRT7 |  | 1.29 | - |
| NM_001493 | GDI1 |  | 1.28 | - |
| AK092921 | - |  | 1.28 | - |
| NM_176795 | - |  | 1.28 | - |
| NM_003164 | STX5 |  | 1.28 | - |
| NM_001823 | CKB |  | 1.27 | - |
| NM_145645 | NSUN5B |  | 1.27 | - |
| NM_004328 | BCS1L |  | 1.27 | - |
| NM_024661 | CCDC51 |  | 1.27 | - |
| BC017996 | - |  | 1.27 | - |
| NM_004688 | NMI |  | 1.26 | - |
| NM_016046 | EXOSC1 |  | 1.26 | - |
| NM_133337 | FER1L3 |  | 1.26 | - |
| BC036909 | LOC284889 |  | 1.26 | - |
| NM_005526 | HSF1 |  | 1.26 | - |
| NM_019884 | GSK3A |  | 1.25 | - |
| NM_004909 | CSAG2 |  | 1.25 | - |
| NM_001069 | TUBB2A |  | 1.25 | - |
| NM_021128 | POLR2L |  | 1.24 | - |
| NM_145249 | - |  | 1.24 | - |
| NM_156036 | HOXB6 |  | 1.24 | - |
| AB002313 | PLXNB2 |  | 1.24 | PLXNB2 |
| AY033611 | - |  | 1.24 | - |
| BG213931 | - |  | 1.24 | - |
| NM_052960 | RBP7 |  | 1.23 | - |
| NM_001030 | RPS27 |  | 1.23 | - |
| NM_177533 | NUDT14 |  | 1.22 | - |
| NM_000108 | DLD |  | -1.23 | - |
| NM_178042 | ACTL6A |  | -1.24 | - |
| NM_017735 | TTC27 |  | -1.24 | - |
| NM_007350 | PHLDA1 |  | -1.25 | PHLDA1 |
| NM_019895 | CLDND1 |  | -1.26 | - |
| NM_006469 | - |  | -1.26 | - |
| NM_024604 | FLJ21908 |  | -1.27 | - |
| NM_002789 | PSMA4 |  | -1.27 | - |
| CR624200 | - |  | -1.27 | - |
| NM_145303 | LOC202459 |  | -1.27 | - |
| AK057359 | LOC146346 |  | -1.27 | - |
| NM_002375 | MAP4 |  | -1.28 | - |
| NM_006100 | ST3GAL6 |  | -1.28 | - |
| NM_001469 | XRCC6 |  | -1.28 | - |
| NM_014017 | MAPBPIP |  | -1.28 | - |
| NM_018361 | AGPAT5 |  | -1.29 | - |
| NM_145808 | MTPN |  | -1.29 | - |
| NM_005898 | GPIAP1 |  | -1.29 | - |
| NM_013233 | STK39 |  | -1.29 | - |
| NM_016548 | GOLPH2 |  | -1.29 | - |
| NM_006726 | LRBA |  | -1.29 | - |
| NM_014390 | SND1 |  | -1.30 | - |
| AK098818 | ZDHHC20 |  | -1.30 | - |
| NM_018374 | TMEM106B |  | -1.31 | - |
| AI792678 | - |  | -1.31 | - |
| BC033829 | - |  | -1.31 | - |
| NM_178812 | MTDH |  | -1.31 | - |
| NM_205842 | NCKAP1 |  | -1.32 | - |
| BG719281 | - |  | -1.32 | - |
| NM_014765 | TOMM20 |  | -1.32 | - |
| NM_013943 | CLIC4 |  | -1.33 | CLIC4 |
| NM_004192 | ASMTL |  | -1.33 | - |
| NM_000414 | HSD17B4 |  | -1.33 | - |
| NM_014865 | CNAP1 |  | -1.33 | - |
| NM_005574 | LMO2 |  | -1.34 | - |
| NM_019063 | EML4 |  | -1.34 | - |
| BC010266 | HNRPA1 |  | -1.34 | - |
| AF130080 | - |  | -1.34 | - |
| NM_000904 | NQO2 |  | -1.35 | - |
| NM_006868 | RAB31 |  | -1.35 | - |
| NM_144726 | FLJ31951 |  | -1.35 | - |
| BX537360 | - |  | -1.35 | - |
| NM_015161 | ARL6IP |  | -1.36 | ARL6IP |
| AB033058 | DLG3 |  | -1.36 | - |
| NM_018834 | - |  | -1.36 | - |
| NM_004134 | HSPA9B |  | -1.36 | - |
| NM_001915 | - |  | -1.37 | - |
| NM_133375 | MGC4562 |  | -1.37 | - |
| NM_175863 | ARID1B |  | -1.37 | - |
| AK055986 | MAPT |  | -1.37 | - |
| NM_032947 | MST150 |  | -1.37 | - |
| NM_174936 | PCSK9 |  | -1.37 | - |
| NM_003816 | ADAM9 |  | -1.39 | - |
| XM_372996 | LOC391555 |  | -1.39 | - |
| NM_017905 | TMCO3 |  | -1.39 | - |
| XM_062025 | - |  | -1.39 | - |
| XM_296117 | - |  | -1.40 | - |
| NM_016270 | KLF2 |  | -1.40 | - |
| NM_007270 | FKBP9 |  | -1.40 | - |
| NM_003071 | SMARCA3 |  | -1.41 | - |
| NM_003786 | - |  | -1.41 | - |
| NM_013322 | SNX10 |  | -1.41 | - |
| NM_007261 | CD300A |  | -1.41 | - |
| BC048298 | TBL1XR1 |  | -1.41 | - |
| NM_014026 | DCPS |  | -1.41 | - |
| NM_005114 | HS3ST1 |  | -1.42 | HS3ST1 |
| NM_006055 | LANCL1 |  | -1.42 | - |
| NM_139076 | CCDC98 |  | -1.43 | - |
| NM_005542 | - |  | -1.43 | - |
| NM_014254 | TMEM5 |  | -1.44 | - |
| AF119896 | - |  | -1.45 | - |
| NM_004990 | MARS |  | -1.45 | - |
| NM_018394 | ABHD10 |  | -1.45 | - |
| NM_031989 | PCBP2 |  | -1.45 | - |
| NM_001432 | EREG |  | -1.46 | - |
| NM_018084 | KIAA1212 |  | -1.46 | - |
| NM_003129 | SQLE |  | -1.46 | - |
| NM_002709 | PPP1CB |  | -1.46 | - |
| NM_006457 | PDLIM5 |  | -1.46 | - |
| NM_002136 | HNRPA1 |  | -1.46 | - |
| NM_001483 | GBAS |  | -1.47 | - |
| NM_005110 | GFPT2 |  | -1.47 | - |
| NM_003546 | HIST1H4L |  | -1.47 | - |
| NM_001677 | ATP1B1 |  | -1.47 | - |
| NM_004563 | PCK2 |  | -1.49 | - |
| XM_293570 | - |  | -1.49 | - |
| NM_016245 | DHRS8 |  | -1.49 | - |
| NM_173685 | C8orf36 |  | -1.51 | - |
| NM_000903 | NQO1 |  | -1.52 | - |
| NM_153332 | THEX1 |  | -1.52 | - |
| NM_005481 | THRAP5 |  | -1.52 | - |
| NM_006547 | IGF2BP3 |  | -1.52 | - |
| NM_003318 | TTK |  | -1.53 | - |
| NM_144497 | AKAP12 |  | -1.53 | AKAP12 |
| NM_033222 | PSIP1 |  | -1.53 | - |
| NM_000245 | MET |  | -1.54 | - |
| NM_003483 | HMGA2 |  | -1.54 | - |
| NM_024665 | TBL1XR1 |  | -1.54 | - |
| CR603865 | - |  | -1.55 | - |
| NM_033360 | KRAS |  | -1.55 | - |
| NM_032466 | ASPH |  | -1.56 | - |
| AK021751 | - |  | -1.56 | - |
| NM_002340 | LSS |  | -1.58 | - |
| NM_016108 | AIG1 |  | -1.59 | - |
| NM_014857 | RABGAP1L |  | -1.60 | - |
| NM_033429 | CALML4 |  | -1.61 | - |
| AK095791 | - |  | -1.61 | - |
| NM_153344 | C6orf141 |  | -1.63 | - |
| NM_139177 | SLC39A11 |  | -1.63 | - |
| NM_014783 | ARHGAP11A |  | -1.64 | - |
| AK021858 | - |  | -1.69 | - |
| NM_000097 | CPOX |  | -1.72 | CPOX |
| CN479126 | ID1 |  | -1.75 | ID1 |
| NM_006047 | RBM12 |  | -1.78 | - |
| NM_145753 | PHLDB2 |  | -1.88 | - |
| NM_080650 | ATPBD4 |  | -1.91 | - |
| NM_001010971 | SAMD13 |  | -1.94 | - |
|  |  |  |  |  |
| **Cisplatin + Chk1 inhibitor Common** | | | | |
|  |  |  |  |  |
| **Accession** | **Symbol** | **FoldChangeT** | **FoldChangeD** | **p53 regulated** |
|  |  |  |  |  |
| NM_004881 | TP53I3 | 7.16 | 6.05 | TP53I3 |
| NM_139314 | - | 3.60 | 5.65 | - |
| NM_017527 | LY6K | 3.61 | 4.50 | - |
| NM_020299 | AKR1B10 | 3.78 | 4.48 | - |
| NM_001511 | CXCL1 | 3.32 | 4.42 | - |
| NM_078467 | CDKN1A | 4.87 | 4.10 | - |
| NM_004864 | GDF15 | 3.71 | 4.02 | GDF15 |
| NM_182507 | LOC144501 | 3.90 | 3.78 | - |
| NM_000389 | CDKN1A | 4.07 | 3.53 | - |
| NM_004024 | ATF3 | 4.60 | 3.49 | ATF3 |
| AK023754 | - | 3.16 | 3.33 | - |
| NM_000228 | - | 2.95 | 3.30 | - |
| NM_002639 | SERPINB5 | 3.16 | 3.29 | SERPINB5 |
| NM_000227 | LAMA3 | 2.74 | 3.22 | - |
| NM_017957 | EPN3 | 2.69 | 3.19 | - |
| NM_022772 | EPS8L2 | 3.16 | 3.17 | - |
| NM_018837 | SULF2 | 3.51 | 3.12 | - |
| NM_004073 | PLK3 | 3.70 | 3.10 | - |
| NM_014568 | GALNT5 | 2.15 | 3.07 | - |
| NM_006142 | SFN | 3.45 | 3.04 | SFN |
| NM_001450 | - | 3.09 | 2.99 | - |
| BC006795 | MGC5370 | 3.21 | 2.96 | - |
| NM_004031 | IRF7 | 2.44 | 2.94 | - |
| BC062776 | - | 2.55 | 2.89 | - |
| NM_000526 | KRT14 | 2.57 | 2.86 | - |
| NM_000693 | ALDH1A3 | 1.62 | 2.75 | - |
| NM_005101 | ISG15 | 2.95 | 2.72 | - |
| NM_004110 | - | 3.13 | 2.71 | - |
| NM_006186 | NR4A2 | 1.79 | 2.68 | - |
| NM_000107 | DDB2 | 3.07 | 2.61 | DDB2 |
| NM_006516 | SLC2A1 | 1.97 | 2.56 | SLC2A1 |
| NM_023915 | GPR87 | 2.46 | 2.55 | - |
| NM_001613 | ACTA2 | 2.50 | 2.51 | ACTA2 |
| NM_002228 | JUN | 2.28 | 2.49 | - |
| AK025631 | - | 2.86 | 2.47 | - |
| NM_023039 | ANKRA2 | 2.27 | 2.46 | - |
| NM_021732 | AVPI1 | 2.29 | 2.45 | - |
| NM_000835 | GRIN2C | 2.47 | 2.43 | - |
| NM_003004 | SECTM1 | 1.77 | 2.41 | - |
| NM_013376 | SERTAD1 | 2.44 | 2.41 | - |
| NM_152426 | APOBEC3D | 2.29 | 2.40 | - |
| NM_031308 | EPPK1 | 2.31 | 2.40 | - |
| NM_201612 | IKIP | 2.32 | 2.39 | - |
| NM_001657 | AREG | 2.02 | 2.39 | - |
| NM_007135 | ZNF79 | 2.48 | 2.36 | - |
| NM_005682 | - | 2.06 | 2.35 | - |
| NM_014278 | HSPA4L | 2.22 | 2.35 | - |
| NM_003620 | PPM1D | 2.42 | 2.31 | PPM1D |
| NM_198545 | C1orf187 | 1.96 | 2.27 | - |
| NM_012396 | PHLDA3 | 3.08 | 2.25 | PHLDA3 |
| NM_006096 | NDRG1 | 1.60 | 2.24 | NDRG1 |
| NM_002275 | KRT15 | 1.81 | 2.24 | KRT15 |
| NM_018370 | FLJ11259 | 2.06 | 2.20 | - |
| NM_014398 | LAMP3 | 1.99 | 2.20 | - |
| AK024898 | - | 2.13 | 2.18 | - |
| NM_001086 | AADAC | 1.97 | 2.17 | - |
| NM_016399 | TRIAP1 | 2.44 | 2.16 | - |
| NM_016357 | LIMA1 | 1.56 | 2.16 | - |
| BE378852 | - | 2.08 | 2.15 | - |
| AK056245 | - | 2.23 | 2.14 | - |
| NM_021137 | TNFAIP1 | 1.93 | 2.13 | - |
| NM_004900 | APOBEC3B | 2.25 | 2.13 | - |
| NM_004138 | KRTHA3A | 1.85 | 2.12 | - |
| NM_016606 | REEP2 | 1.78 | 2.11 | - |
| NM_000043 | FAS | 2.03 | 2.11 | FAS |
| NM_004292 | RIN1 | 2.07 | 2.11 | - |
| NM_006504 | PTPRE | 2.05 | 2.10 | - |
| NM_001554 | CYR61 | 3.26 | 2.10 | - |
| XM_291270 | - | 1.96 | 2.10 | - |
| NM_021005 | NR2F2 | 1.57 | 2.09 | - |
| NM_007110 | TEP1 | 1.99 | 2.09 | - |
| NM_004472 | FOXD1 | 1.84 | 2.08 | - |
| NM_001670 | ARVCF | 2.09 | 2.07 | - |
| NM_021615 | CHST6 | 1.90 | 2.07 | - |
| NM_032463 | LAT2 | 1.66 | 2.07 | - |
| NM_004419 | DUSP5 | 2.17 | 2.04 | DUSP5 |
| NM_032876 | JUB | 1.93 | 2.04 | - |
| NM_001009991 | SYTL3 | 2.02 | 2.03 | - |
| NM_001924 | GADD45A | 2.25 | 2.03 | GADD45A |
| NM_016201 | AMOTL2 | 2.74 | 2.02 | - |
| NM_002201 | ISG20 | 1.88 | 2.02 | - |
| NM_004431 | EPHA2 | 1.89 | 2.02 | EPHA2 |
| NM_014578 | RHOD | 2.35 | 2.01 | - |
| NM_004561 | OVOL1 | 1.65 | 2.00 | - |
| NM_003584 | DUSP11 | 1.72 | 2.00 | - |
| NM_000593 | TAP1 | 1.93 | 1.98 | TAP1 |
| NM_001002914 | KCTD11 | 1.95 | 1.98 | - |
| NM_019556 | MOSPD1 | 1.61 | 1.97 | - |
| NM_000952 | PTAFR | 6.10 | 1.96 | - |
| NM_001004431 | METRNL | 1.71 | 1.95 | - |
| NM_002231 | CD82 | 1.76 | 1.95 | - |
| NM_006622 | PLK2 | 3.66 | 1.94 | PLK2 |
| AW972815 | - | 1.94 | 1.94 | - |
| XM_290516 | - | 1.94 | 1.93 | - |
| NM_012242 | DKK1 | 1.63 | 1.92 | DKK1 |
| NM_003842 | TNFRSF10B | 2.13 | 1.92 | TNFRSF10B |
| NM_006663 | PPP1R13L | 1.60 | 1.92 | - |
| CR749856 | LOC388558 | 1.85 | 1.91 | - |
| NM_025194 | ITPKC | 1.68 | 1.91 | - |
| BC007947 | - | 1.79 | 1.91 | - |
| NM_014454 | SESN1 | 2.10 | 1.90 | SESN1 |
| NM_021127 | PMAIP1 | 2.24 | 1.90 | PMAIP1 |
| NM_001312 | CRIP2 | 1.92 | 1.90 | - |
| NM_000676 | ADORA2B | 1.83 | 1.89 | - |
| NM_003975 | SH2D2A | 1.81 | 1.88 | - |
| NM_203302 | MGC70863 | 1.80 | 1.88 | - |
| NM_032772 | ZNF503 | 1.46 | 1.86 | - |
| AB041269 | LOC160313 | 1.59 | 1.85 | - |
| NM_004148 | NINJ1 | 1.76 | 1.85 | - |
| NM_020947 | KIAA1609 | 1.62 | 1.85 | - |
| NM_014475 | DHDH | 1.51 | 1.85 | - |
| NM_000581 | - | 1.98 | 1.84 | - |
| NM_004750 | CRLF1 | 1.72 | 1.84 | - |
| NM_033285 | TP53INP1 | 1.92 | 1.84 | - |
| NM_024101 | MLPH | 1.42 | 1.83 | - |
| NM_032324 | C1orf57 | 1.80 | 1.83 | - |
| NM_003714 | STC2 | 1.45 | 1.82 | - |
| NM_182919 | TICAM1 | 1.68 | 1.82 | - |
| NM_199132 | ZNF468 | 1.85 | 1.82 | - |
| NM_032510 | PARD6G | 1.63 | 1.80 | - |
| BC040043 | - | 1.73 | 1.80 | - |
| NM_175744 | RHOC | 1.75 | 1.80 | - |
| NM_080821 | C20orf108 | 1.43 | 1.79 | - |
| NM_007026 | DUSP14 | 1.78 | 1.79 | - |
| NM_032102 | SRP46 | 1.82 | 1.78 | - |
| NM_003516 | HIST2H2AA3 | 2.20 | 1.78 | - |
| NM_022873 | IFI6 | 2.05 | 1.77 | - |
| NM_006736 | DNAJB2 | 1.55 | 1.75 | - |
| NM_004760 | STK17A | 1.71 | 1.75 | - |
| NM_004165 | RRAD | 1.64 | 1.75 | - |
| NM_006404 | PROCR | 1.85 | 1.74 | - |
| NM_016423 | ZNF219 | 1.79 | 1.74 | - |
| NM_030912 | TRIM8 | 1.61 | 1.74 | - |
| NM_015920 | RPS27L | 2.56 | 1.73 | - |
| NM_002135 | NR4A1 | 2.25 | 1.73 | - |
| NM_001311 | CRIP1 | 1.96 | 1.73 | - |
| AF132197 | - | 1.72 | 1.73 | - |
| NM_006426 | DPYSL4 | 2.05 | 1.73 | - |
| NM_014301 | NIFUN | 1.74 | 1.73 | - |
| AB058761 | ZNF469 | 1.50 | 1.73 | - |
| NM_003651 | CSDA | 1.64 | 1.72 | - |
| NM_001008401 | FLJ16231 | 1.79 | 1.72 | - |
| NM_005978 | S100A2 | 2.29 | 1.72 | - |
| NM_001548 | IFIT1 | 1.85 | 1.72 | - |
| NM_002125 | HLA-DRB5 | 1.69 | 1.71 | - |
| NM_003979 | GPRC5A | 2.14 | 1.71 | - |
| NM_004293 | GDA | 1.34 | 1.71 | - |
| NM_001853 | COL9A3 | 1.82 | 1.70 | - |
| NM_016291 | IHPK2 | 1.74 | 1.70 | - |
| NM_004324 | - | 1.86 | 1.70 | - |
| NM_033120 | NKD2 | 1.48 | 1.69 | - |
| NM_004636 | SEMA3B | 1.61 | 1.69 | - |
| NM_006698 | BLCAP | 1.64 | 1.68 | - |
| NM_006509 | RELB | 1.52 | 1.68 | - |
| NM_001430 | EPAS1 | 1.31 | 1.68 | - |
| NM_005749 | TOB1 | 1.54 | 1.67 | - |
| NM_173809 | - | 1.65 | 1.67 | - |
| NM_014045 | LRP10 | 1.51 | 1.67 | - |
| NM_133180 | EPS8L1 | 1.60 | 1.66 | - |
| NM_016545 | IER5 | 1.81 | 1.66 | - |
| AK024480 | - | 1.55 | 1.66 | - |
| NM_030927 | TSPAN14 | 1.63 | 1.66 | - |
| NM_004417 | DUSP1 | 1.88 | 1.66 | DUSP1 |
| NM_024310 | PLEKHF1 | 1.59 | 1.66 | - |
| NM_022767 | ISG20L1 | 2.07 | 1.65 | - |
| NM_002658 | PLAU | 1.48 | 1.65 | - |
| NM_138764 | BAX | 1.75 | 1.65 | BAX |
| NM_000476 | AK1 | 2.16 | 1.65 | AK1 |
| NM_001550 | - | 1.80 | 1.65 | - |
| NM_004999 | MYO6 | 1.39 | 1.64 | - |
| NM_020375 | C12orf5 | 1.89 | 1.64 | - |
| NM_153337 | - | 1.52 | 1.64 | - |
| NM_005319 | HIST1H1C | 1.66 | 1.64 | - |
| NM_000713 | BLVRB | 1.36 | 1.63 | - |
| NM_016323 | HERC5 | 1.63 | 1.63 | - |
| NM_139266 | STAT1 | 1.59 | 1.63 | - |
| AK054960 | - | 1.91 | 1.63 | - |
| NM_006806 | BTG3 | 1.76 | 1.63 | - |
| NM_016352 | CPA4 | 2.57 | 1.63 | - |
| NM_033245 | PML | 1.89 | 1.62 | - |
| NM_006187 | OAS3 | 1.97 | 1.62 | - |
| NM_153256 | C10orf47 | 1.40 | 1.62 | - |
| NM_003761 | VAMP8 | 1.59 | 1.62 | - |
| NM_152640 | DCP1B | 1.50 | 1.61 | - |
| AL365412 | - | 1.57 | 1.61 | - |
| NM_014994 | MAPKBP1 | 1.59 | 1.61 | - |
| NM_014172 | PHPT1 | 1.74 | 1.60 | - |
| NM_016029 | DHRS7 | 1.28 | 1.60 | - |
| NM_013937 | OR11A1 | 1.54 | 1.59 | - |
| NM_021825 | MDS025 | 1.64 | 1.59 | - |
| NM_003302 | TRIP6 | 1.83 | 1.59 | - |
| BC000632 | - | 1.69 | 1.59 | - |
| NM_032872 | SYTL1 | 1.44 | 1.59 | - |
| NM_014417 | BBC3 | 1.57 | 1.59 | BBC3 |
| NM_003869 | CES2 | 1.48 | 1.58 | - |
| BC032478 | MAP2K3 | 1.57 | 1.58 | - |
| NM_005620 | S100A11 | 1.52 | 1.58 | - |
| NM_018244 | C20orf44 | 1.49 | 1.58 | - |
| NM_031904 | FKSG44 | 1.70 | 1.58 | - |
| NM_002292 | LAMB2 | 1.37 | 1.58 | - |
| NM_012081 | ELL2 | 1.55 | 1.57 | - |
| NM_138809 | LOC134147 | 1.57 | 1.57 | - |
| NM_024015 | HOXB4 | 1.59 | 1.57 | - |
| NM_002592 | PCNA | 1.82 | 1.57 | PCNA |
| NM_138768 | MYEOV | 1.51 | 1.56 | - |
| NM_003992 | CLK3 | 1.47 | 1.56 | - |
| NM_145323 | OSBPL3 | 1.73 | 1.56 | - |
| NM_001122 | ADFP | 1.55 | 1.56 | - |
| NM_032034 | SLC4A11 | 1.51 | 1.56 | - |
| NM_014860 | SUPT7L | 1.46 | 1.56 | - |
| NM_018161 | NADSYN1 | 1.76 | 1.56 | - |
| XM_377928 | - | 1.56 | 1.55 | - |
| NM_004161 | RAB1A | 1.29 | 1.54 | - |
| NM_006831 | HEAB | 1.56 | 1.54 | - |
| NM_012079 | DGAT1 | 1.52 | 1.54 | - |
| NM_020728 | FAM62B | 1.35 | 1.54 | - |
| NM_203394 | E2F7 | 1.83 | 1.54 | - |
| NM_198457 | ZNF600 | 1.51 | 1.54 | - |
| AK091153 | - | 1.37 | 1.53 | - |
| NM_014376 | CYFIP2 | 1.57 | 1.52 | CYFIP2 |
| NM_021913 | AXL | 1.55 | 1.52 | - |
| NM_003273 | TM7SF2 | 1.43 | 1.52 | - |
| NM_001983 | ERCC1 | 1.50 | 1.52 | ERCC1 |
| NM_007315 | STAT1 | 1.39 | 1.52 | - |
| NM_174905 | FAM98C | 1.69 | 1.52 | - |
| NM_052951 | DNTTIP1 | 1.27 | 1.52 | - |
| NM_031449 | DKFZp761I2123 | 1.31 | 1.52 | - |
| NM_004281 | BAG3 | 1.30 | 1.51 | - |
| NM_024041 | - | 1.44 | 1.51 | - |
| NM_019112 | ABCA7 | 1.66 | 1.51 | - |
| NM_001197 | BIK | 1.63 | 1.51 | BIK |
| NM_016368 | ISYNA1 | 1.66 | 1.50 | - |
| NM_001878 | CRABP2 | 1.58 | 1.50 | - |
| NM_016449 | LOC51233 | 1.34 | 1.50 | - |
| NM_002134 | HMOX2 | 1.44 | 1.49 | - |
| NM_003449 | TRIM26 | 1.53 | 1.49 | - |
| NM_031207 | HYI | 1.59 | 1.49 | - |
| NM_000434 | NEU1 | 1.50 | 1.48 | - |
| NM_000158 | GBE1 | 1.34 | 1.48 | - |
| NM_004370 | COL12A1 | 1.67 | 1.47 | - |
| NM_198446 | C1orf122 | 1.62 | 1.47 | - |
| AK092260 | DUSP5P | 1.49 | 1.47 | - |
| NM_000820 | GAS6 | 1.61 | 1.47 | - |
| NM_006425 | SLU7 | 1.40 | 1.47 | - |
| NM_015675 | GADD45B | 1.58 | 1.47 | - |
| NM_015399 | BRMS1 | 1.49 | 1.46 | - |
| NM_024723 | - | 1.52 | 1.46 | - |
| BC014776 | - | 1.69 | 1.46 | - |
| BC069216 | - | 1.36 | 1.46 | - |
| NM_001010925 | ANKRD19 | 1.57 | 1.45 | - |
| CR613736 | KRT8 | 1.58 | 1.45 | - |
| NM_002997 | - | 1.66 | 1.45 | - |
| NM_004879 | EI24 | 1.40 | 1.45 | EI24 |
| NM_015246 | MGRN1 | 1.54 | 1.44 | - |
| NM_003858 | CCNK | 1.43 | 1.44 | - |
| NM_004993 | ATXN3 | 1.48 | 1.43 | - |
| NM_005168 | RND3 | 1.48 | 1.43 | - |
| BX476001 | - | 1.46 | 1.43 | - |
| NM_005532 | IFI27 | 2.81 | 1.42 | - |
| NM_005562 | LAMC2 | 1.28 | 1.42 | - |
| AL117478 | - | 1.27 | 1.42 | - |
| NM_033520 | C19orf33 | 1.55 | 1.42 | - |
| NM_021034 | IFITM3 | 1.49 | 1.42 | - |
| NM_052871 | MGC4677 | 1.71 | 1.41 | - |
| XM_497663 | - | 1.50 | 1.41 | - |
| BX538238 | MALAT1 | 1.38 | 1.41 | - |
| NM_000224 | KRT18 | 1.49 | 1.41 | - |
| NM_000854 | GSTT2 | 1.38 | 1.41 | - |
| NM_007275 | TUSC2 | 1.51 | 1.41 | - |
| NM_005572 | LMNA | 1.32 | 1.40 | - |
| AK096811 | - | 1.33 | 1.40 | - |
| NM_006435 | IFITM2 | 1.48 | 1.40 | - |
| AK091178 | - | 1.60 | 1.40 | - |
| NM_145886 | - | 2.00 | 1.40 | - |
| NM_004295 | TRAF4 | 1.66 | 1.39 | TRAF4 |
| NM_021077 | NMB | 1.49 | 1.39 | - |
| NM_006802 | SF3A3 | 1.41 | 1.39 | - |
| NM_003900 | SQSTM1 | 1.34 | 1.39 | - |
| NM_000449 | RFX5 | 1.36 | 1.39 | - |
| NM_002818 | PSME2 | 1.37 | 1.39 | - |
| NM_022370 | ROBO3 | 1.35 | 1.39 | - |
| NM_032525 | TUBB6 | 1.40 | 1.39 | - |
| NM_005689 | ABCB6 | 1.46 | 1.38 | - |
| NM_002117 | HLA-C | 1.35 | 1.38 | - |
| NM_004707 | ATG12 | 1.37 | 1.38 | - |
| NM_001747 | CAPG | 1.28 | 1.38 | - |
| NM_018447 | TMEM111 | 1.32 | 1.38 | - |
| XM_498018 | - | 1.54 | 1.38 | - |
| XM_371762 | - | 1.37 | 1.37 | - |
| M21963 | HLA-C | 1.35 | 1.37 | - |
| NR_001590 | IFITM4P | 1.46 | 1.37 | - |
| NM_024667 | VPS37B | 1.35 | 1.36 | - |
| AK125231 | - | 1.34 | 1.36 | - |
| NM_052901 | - | 1.43 | 1.36 | - |
| NM_002133 | HMOX1 | 1.47 | 1.36 | - |
| NM_172199 | - | 1.49 | 1.36 | - |
| NM_020418 | - | 1.49 | 1.35 | - |
| NM_145201 | NAPRT1 | 1.25 | 1.35 | - |
| NM_017491 | WDR1 | 1.33 | 1.34 | - |
| NM_002127 | HLA-G | 1.31 | 1.34 | - |
| NM_001259 | CDK6 | 1.37 | 1.34 | - |
| M28016 | - | 1.62 | 1.33 | - |
| NM_012232 | PTRF | 1.41 | 1.33 | - |
| NM_006384 | CIB1 | 1.31 | 1.33 | - |
| NM_002359 | MAFG | 1.30 | 1.33 | - |
| NM_001694 | ATP6V0C | 1.39 | 1.32 | - |
| NM_015219 | - | 1.40 | 1.32 | - |
| NM_006058 | TNIP1 | 1.34 | 1.32 | - |
| NM_018043 | TMEM16A | 1.50 | 1.32 | - |
| AF168717 | DTWD1 | 1.31 | 1.31 | - |
| NM_000169 | GLA | 1.48 | 1.31 | - |
| NM_001625 | AK2 | 1.41 | 1.31 | - |
| NM_003254 | TIMP1 | 1.30 | 1.31 | - |
| NM_016062 | FAM96B | 1.37 | 1.30 | - |
| NM_015356 | - | 1.28 | 1.30 | - |
| NM_015953 | NOSIP | 1.26 | 1.30 | - |
| NM_014297 | ETHE1 | 1.32 | 1.29 | - |
| BC079831 | LOC284184 | 1.62 | 1.29 | - |
| AF391805 | - | 1.47 | 1.29 | - |
| NM_021075 | NDUFV3 | 1.24 | 1.28 | - |
| XM_294476 | - | 1.35 | 1.28 | - |
| NM_000156 | GAMT | 1.27 | 1.27 | - |
| NM_002166 | ID2 | 1.26 | 1.26 | ID2 |
| NM_001022 | RPS19 | 1.40 | 1.26 | - |
| NM_021173 | POLD4 | 1.43 | 1.26 | - |
| NM_006000 | TUBA1 | 1.45 | 1.26 | - |
| NM_005514 | HLA-B | 1.38 | 1.26 | - |
| NM_002896 | RBM4 | 1.41 | 1.25 | - |
| U41420 | HLA-C | 1.31 | 1.24 | - |
| BC010117 | - | 1.30 | 1.24 | - |
| NM_152318 | C12orf45 | 1.44 | 1.23 | - |
| NM_021237 | SELK | 1.31 | 1.23 | - |
| NM_007152 | ZNF195 | 1.23 | 1.21 | - |
| X56841 | - | 1.21 | 1.21 | - |
| NM_007273 | PHB2 | -1.22 | -1.24 | - |
| NM_006793 | PRDX3 | -1.33 | -1.24 | - |
| NM_182760 | SUMF1 | -1.40 | -1.24 | - |
| NM_001358 | DHX15 | -1.36 | -1.25 | - |
| NM_031157 | HNRPA1 | -1.36 | -1.25 | - |
| NM_005500 | SAE1 | -1.28 | -1.25 | - |
| XM_379885 | LOC402562 | -1.36 | -1.28 | - |
| NM_002493 | - | -1.27 | -1.28 | - |
| NM_181578 | RFC5 | -1.21 | -1.28 | - |
| NM_012227 | GTPBP6 | -1.33 | -1.30 | - |
| NM_005911 | MAT2A | -1.31 | -1.30 | - |
| NM_004559 | YBX1 | -1.26 | -1.31 | - |
| NM_153201 | HSPA8 | -1.24 | -1.31 | HSPA8 |
| NM_001979 | EPHX2 | -1.40 | -1.31 | - |
| BC062731 | - | -1.32 | -1.31 | - |
| NM_001533 | HNRPL | -1.33 | -1.32 | - |
| NM_194247 | HNRPA3 | -1.73 | -1.32 | - |
| NM_001004419 | CLEC2D | -1.37 | -1.34 | - |
| NM_017512 | ENOSF1 | -1.28 | -1.34 | - |
| NM_005826 | HNRPR | -1.30 | -1.34 | - |
| NM_172164 | NASP | -1.34 | -1.35 | - |
| NM_018245 | OGDHL | -1.40 | -1.35 | - |
| NM_002902 | RCN2 | -1.34 | -1.36 | - |
| NM_003011 | SET | -1.40 | -1.36 | - |
| NM_012201 | GLG1 | -1.54 | -1.36 | - |
| NM_080546 | SLC44A1 | -1.49 | -1.36 | - |
| NM_005038 | PPID | -1.36 | -1.36 | - |
| NM_001970 | EIF5A | -1.48 | -1.37 | - |
| NM_203380 | ACSL5 | -1.79 | -1.37 | - |
| NM_005434 | MALL | -1.44 | -1.38 | - |
| XM_379108 | - | -1.45 | -1.38 | - |
| BC009038 | - | -1.37 | -1.38 | - |
| NM_139207 | NAP1L1 | -1.48 | -1.38 | - |
| NM_033070 | CECR5 | -1.26 | -1.38 | - |
| NM_002520 | NPM1 | -1.43 | -1.39 | - |
| NM_014505 | KCNMB4 | -1.45 | -1.39 | - |
| BC045572 | - | -1.41 | -1.39 | - |
| NM_004176 | - | -1.61 | -1.39 | - |
| NM_018024 | C8orf32 | -1.39 | -1.39 | - |
| BX647217 | HNRPA3 | -1.65 | -1.40 | - |
| NM_006324 | CFDP1 | -1.46 | -1.40 | - |
| NM_006597 | HSPA8 | -1.27 | -1.40 | HSPA8 |
| NM_005869 | SDCCAG10 | -1.35 | -1.40 | - |
| NM_031466 | NIBP | -1.55 | -1.40 | - |
| NM_032737 | LMNB2 | -1.38 | -1.41 | - |
| CR616535 | - | -1.48 | -1.41 | - |
| NM_032814 | TMEM118 | -1.44 | -1.41 | - |
| NM_138369 | FAM44B | -1.50 | -1.41 | - |
| NM_032343 | CHCHD6 | -1.41 | -1.41 | - |
| NM_014412 | CACYBP | -1.36 | -1.41 | - |
| NM_030579 | CYB5B | -1.45 | -1.41 | - |
| NM_003539 | HIST1H4D | -1.49 | -1.42 | - |
| NM_002137 | HNRPA2B1 | -1.37 | -1.42 | - |
| NM_003542 | HIST1H4C | -1.50 | -1.42 | - |
| NM_145729 | MRPL24 | -1.36 | -1.43 | - |
| AK098256 | - | -1.41 | -1.43 | - |
| NM_054016 | FUSIP1 | -1.40 | -1.43 | - |
| NM_016587 | CBX3 | -1.34 | -1.43 | - |
| XM_496355 | - | -1.51 | -1.43 | - |
| NM_199185 | NPM1 | -1.50 | -1.44 | - |
| NM_003544 | HIST1H4B | -1.48 | -1.44 | - |
| CR616772 | HSP90AB1 | -1.56 | -1.44 | HSP90AB1 |
| NM_021066 | HIST1H2AJ | -1.29 | -1.44 | - |
| NM_002156 | HSPD1 | -1.49 | -1.44 | - |
| NM_002128 | HMGB1 | -1.44 | -1.45 | - |
| NM_000179 | MSH6 | -1.41 | -1.45 | - |
| NM_003104 | SORD | -1.61 | -1.45 | - |
| CR620336 | - | -1.46 | -1.45 | - |
| XM_047355 | DCAMKL3 | -1.45 | -1.45 | - |
| NM_145697 | CDCA1 | -1.54 | -1.45 | - |
| NM_005381 | NCL | -1.24 | -1.45 | - |
| NM_001280 | CIRBP | -1.44 | -1.46 | - |
| NM_007355 | HSP90AB1 | -1.62 | -1.46 | HSP90AB1 |
| NM_003486 | SLC7A5 | -1.34 | -1.46 | - |
| NM_018101 | CDCA8 | -1.30 | -1.47 | - |
| NM_006401 | ANP32B | -1.56 | -1.47 | - |
| NM_005993 | TBCD | -1.34 | -1.48 | - |
| NM_018492 | PBK | -1.45 | -1.48 | - |
| NM_007235 | XPOT | -1.54 | -1.48 | - |
| NM_173609 | - | -1.28 | -1.48 | - |
| BE898801 | - | -1.57 | -1.49 | - |
| NM_003017 | SFRS3 | -1.53 | -1.49 | SFRS3 |
| NM_002073 | GNAZ | -1.56 | -1.49 | - |
| NM_080820 | HARS2 | -1.67 | -1.49 | - |
| BG178211 | - | -1.62 | -1.49 | - |
| NM_005517 | HMGN2 | -1.30 | -1.50 | HMGN2 |
| NM_013238 | DNAJC15 | -1.36 | -1.50 | - |
| NM_002296 | LBR | -1.68 | -1.50 | - |
| AB020684 | - | -1.51 | -1.50 | - |
| NM_024420 | PLA2G4A | -1.73 | -1.51 | - |
| NM_000282 | PCCA | -1.73 | -1.51 | - |
| NM_203434 | IER5L | -1.51 | -1.51 | - |
| NM_004516 | ILF3 | -1.47 | -1.52 | - |
| NM_017867 | C4orf27 | -1.52 | -1.52 | - |
| NM_145269 | - | -1.50 | -1.52 | - |
| NM_003662 | PIR | -1.57 | -1.52 | - |
| NM_014595 | NT5C | -1.40 | -1.53 | - |
| NM_005327 | HADHSC | -1.51 | -1.53 | - |
| BC090057 | - | -1.39 | -1.53 | - |
| XM_497271 | HMGB1 | -1.54 | -1.53 | - |
| NM_006807 | CBX1 | -1.39 | -1.53 | - |
| AY007110 | - | -1.48 | -1.54 | - |
| NM_153824 | PYCR1 | -1.50 | -1.55 | - |
| NM_003113 | SP100 | -1.34 | -1.55 | - |
| BQ317309 | - | -1.52 | -1.55 | - |
| AI015919 | - | -1.50 | -1.55 | - |
| NM_030920 | ANP32E | -1.68 | -1.55 | - |
| XM_498178 | - | -1.47 | -1.56 | - |
| NM_022126 | LHPP | -2.01 | -1.56 | - |
| NM_001827 | CKS2 | -1.40 | -1.57 | - |
| AF170294 | PTMAP7 | -1.60 | -1.58 | - |
| NM_002823 | PTMA | -1.57 | -1.58 | - |
| NM_006276 | SFRS7 | -1.52 | -1.60 | - |
| NM_006739 | MCM5 | -1.36 | -1.60 | - |
| NM_031942 | CDCA7 | -1.46 | -1.60 | - |
| NM_032565 | EBPL | -1.44 | -1.61 | - |
| NM_031372 | HNRPDL | -1.55 | -1.61 | - |
| NM_004483 | GCSH | -1.64 | -1.61 | - |
| AK092810 | - | -1.63 | -1.62 | - |
| NM_016343 | CENPF | -1.63 | -1.62 | - |
| NM_006903 | - | -1.88 | -1.62 | - |
| XM_372040 | - | -2.15 | -1.64 | - |
| NM_001673 | ASNS | -2.10 | -1.64 | - |
| NM_002138 | HNRPD | -1.66 | -1.64 | - |
| NM_022731 | NUCKS1 | -1.58 | -1.65 | - |
| NM_018136 | ASPM | -1.68 | -1.66 | - |
| NM_015120 | ALMS1 | -1.61 | -1.67 | - |
| NM_152562 | CDCA2 | -1.45 | -1.67 | - |
| NM_006743 | - | -1.62 | -1.68 | - |
| NM_031370 | HNRPD | -1.73 | -1.69 | - |
| NM_017425 | SPA17 | -1.66 | -1.69 | - |
| NM_004701 | CCNB2 | -1.61 | -1.69 | - |
| XM_379625 | - | -1.49 | -1.69 | - |
| NM_005794 | - | -1.64 | -1.69 | - |
| NM_080927 | DCBLD2 | -1.81 | -1.70 | - |
| NM_031299 | CDCA3 | -1.39 | -1.72 | - |
| NM_206808 | CLYBL | -2.17 | -1.72 | - |
| NM_002106 | H2AFZ | -1.50 | -1.72 | - |
| NM_014750 | DLG7 | -1.70 | -1.73 | - |
| NM_012458 | TIMM13 | -1.36 | -1.74 | - |
| NM_002961 | S100A4 | -1.57 | -1.75 | S100A4 |
| NM_006716 | DBF4 | -1.63 | -1.76 | - |
| NM_182908 | DHRS2 | -1.80 | -1.76 | - |
| NM_024808 | FLJ22624 | -1.56 | -1.76 | - |
| NM_002129 | HMGB2 | -1.41 | -1.76 | - |
| NM_003600 | - | -1.58 | -1.77 | - |
| NM_005239 | ETS2 | -1.61 | -1.77 | - |
| NM_002165 | ID1 | -1.92 | -1.77 | ID1 |
| NM_005915 | MCM6 | -1.50 | -1.78 | - |
| BC044619 | FLJ42709 | -1.90 | -1.79 | - |
| NM_004853 | STX8 | -2.19 | -1.79 | - |
| NM_032636 | PSRC1 | -1.65 | -1.79 | - |
| NM_001168 | - | -1.53 | -1.80 | - |
| NM_003107 | SOX4 | -1.54 | -1.80 | - |
| NM_022743 | SMYD3 | -2.03 | -1.81 | - |
| NM_178448 | C9orf140 | -1.50 | -1.83 | - |
| NM_001453 | FOXC1 | -2.05 | -1.83 | - |
| NM_033661 | WDR4 | -1.67 | -1.84 | - |
| NM_000218 | KCNQ1 | -2.02 | -1.84 | - |
| NM_005063 | SCD | -2.00 | -1.85 | - |
| NM_207418 | - | -1.82 | -1.87 | - |
| NM_006026 | H1FX | -1.70 | -1.88 | - |
| NM_024042 | METRN | -1.45 | -1.90 | - |
| NM_004457 | ACSL3 | -2.33 | -1.90 | ACSL3 |
| NM_018205 | LRRC20 | -1.98 | -1.93 | - |
| NM_021009 | UBC | 1.42 | -1.93 | - |
| CR626729 | - | -1.51 | -1.94 | - |
| AF132203 | SCD | -2.30 | -1.95 | - |
| NM_005733 | KIF20A | -1.94 | -1.96 | - |
| NM_033515 | ARHGAP18 | -2.27 | -1.99 | - |
| NM_031966 | CCNB1 | -2.09 | -2.01 | CCNB1 |
| NM_004336 | BUB1 | -1.90 | -2.02 | BUB1 |
| NM_001255 | CDC20 | -1.72 | -2.04 | - |
| BC041925 | SLC7A11 | -2.21 | -2.04 | - |
| NM_207578 | PRKACB | -2.11 | -2.05 | - |
| AW946823 | SOX4 | -1.91 | -2.16 | - |
| NM_005030 | PLK1 | -1.64 | -2.16 | PLK1 |
| CA310244 | HIST1H2BN | -1.73 | -2.21 | - |
| BF511442 | HIST1H2BD | -1.85 | -2.23 | - |
| NM_018669 | - | -2.01 | -2.26 | - |
| NM_018291 | FLJ10986 | -2.09 | -2.32 | - |
| NM_014899 | RHOBTB3 | -1.99 | -2.37 | - |
| AF108138 | - | -2.63 | -2.93 | - |
| NM_005654 | NR2F1 | -2.79 | -3.62 | - |
|  |  |  |  |  |
|  |  |  |  |  |
| **Cisplatin + Chk1 inhibitor Tetraploid specific** | | | | |
|  |  |  |  |  |
| **Accession** | **Symbol** | **FoldChangeT** |  | **p53 regulated** |
|  |  |  |  |  |
| NM_002526 | NT5E | 2.91 |  | - |
| NM_000499 | CYP1A1 | 2.83 |  | - |
| NM_005329 | HAS3 | 2.82 |  | - |
| AL833872 | - | 2.67 |  | - |
| NM_002952 | RPS2 | 2.29 |  | - |
| NM_007021 | C10orf10 | 2.15 |  | - |
| NM_003236 | TGFA | 2.10 |  | TGFA |
| NM_004425 | ECM1 | 2.10 |  | - |
| NM_005581 | BCAM | 2.09 |  | - |
| NM_001345 | - | 1.98 |  | - |
| NM_024508 | ZBED2 | 1.96 |  | - |
| NM_005971 | FXYD3 | 1.94 |  | FXYD3 |
| NM_000147 | FUCA1 | 1.91 |  | - |
| NM_001172 | ARG2 | 1.90 |  | - |
| AB033060 | AHRR | 1.89 |  | - |
| NM_022164 | TINAGL1 | 1.88 |  | - |
| BC001973 | - | 1.86 |  | - |
| NM_003567 | BCAR3 | 1.86 |  | - |
| NM_024009 | GJB3 | 1.84 |  | - |
| NM_032943 | SYTL2 | 1.83 |  | - |
| X82545 | - | 1.81 |  | - |
| NM_003921 | BCL10 | 1.80 |  | - |
| NM_005953 | MT2A | 1.80 |  | - |
| BC007034 | - | 1.79 |  | - |
| NM_003890 | FCGBP | 1.79 |  | - |
| NM_022168 | IFIH1 | 1.79 |  | - |
| NM_015508 | TIPARP | 1.79 |  | - |
| AF217966 | - | 1.78 |  | - |
| NM_002842 | PTPRH | 1.76 |  | - |
| BC038432 | - | 1.76 |  | - |
| NM_020448 | NPAL3 | 1.75 |  | - |
| NM_015713 | RRM2B | 1.75 |  | RRM2B |
| NM_004628 | XPC | 1.71 |  | XPC |
| NM_007283 | MGLL | 1.71 |  | - |
| NM_003670 | BHLHB2 | 1.70 |  | - |
| AB095939 | - | 1.70 |  | - |
| NM_025249 | KIAA1683 | 1.68 |  | - |
| NM_006084 | ISGF3G | 1.68 |  | - |
| NM_001993 | F3 | 1.68 |  | - |
| NM_018425 | PI4KII | 1.67 |  | - |
| NM_002032 | FTH1 | 1.66 |  | - |
| AF086414 | - | 1.65 |  | - |
| NR_002205 | FTHL12 | 1.65 |  | - |
| NM_033027 | AXUD1 | 1.64 |  | - |
| NM_020672 | S100A14 | 1.63 |  | - |
| NM_004403 | DFNA5 | 1.63 |  | - |
| NM_005485 | - | 1.62 |  | - |
| NM_007150 | ZNF185 | 1.62 |  | - |
| NM_005242 | F2RL1 | 1.61 |  | - |
| NM_000805 | GAST | 1.61 |  | - |
| NM_021260 | ZFYVE1 | 1.61 |  | - |
| NM_145906 | RIOK3 | 1.61 |  | - |
| NM_002999 | SDC4 | 1.60 |  | - |
| NM_015954 | DERA | 1.60 |  | - |
| NM_080920 | GGTLA4 | 1.60 |  | - |
| NM_005755 | EBI3 | 1.60 |  | - |
| NM_172229 | - | 1.60 |  | - |
| U68019 | SMAD3 | 1.60 |  | - |
| NM_030949 | PPP1R14C | 1.60 |  | - |
| AK098314 | - | 1.60 |  | - |
| NM_001007139 | - | 1.58 |  | - |
| U14391 | MYO1E | 1.58 |  | - |
| NM_015444 | RIS1 | 1.58 |  | - |
| NM_016627 | AMZ2 | 1.57 |  | - |
| NM_033380 | COL4A5 | 1.57 |  | - |
| NM_012323 | MAFF | 1.57 |  | - |
| NM_003196 | TCEA3 | 1.57 |  | - |
| NM_177478 | FTMT | 1.56 |  | - |
| NM_182532 | TMEM61 | 1.56 |  | - |
| NM_001001661 | ZNF425 | 1.56 |  | - |
| NM_018381 | FLJ11286 | 1.56 |  | - |
| AL832534 | - | 1.56 |  | - |
| NM_032957 | - | 1.56 |  | - |
| NM_014010 | ASTN2 | 1.55 |  | - |
| BC063625 | KRTAP2-4 | 1.55 |  | - |
| NM_005490 | SH2D3A | 1.55 |  | - |
| NM_005950 | MT1G | 1.54 |  | - |
| NM_006640 | 9-sep | 1.54 |  | - |
| NM_024618 | - | 1.53 |  | - |
| NM_014338 | PISD | 1.53 |  | - |
| NM_015996 | SIDT2 | 1.53 |  | - |
| NM_005952 | MT1X | 1.53 |  | - |
| NM_153032 | - | 1.53 |  | - |
| NM_001153 | ANXA4 | 1.53 |  | - |
| BC012203 | - | 1.52 |  | - |
| NM_031885 | BBS2 | 1.52 |  | - |
| NM_033388 | ATG16L2 | 1.51 |  | - |
| NM_002230 | JUP | 1.51 |  | - |
| NM_003029 | SHC1 | 1.51 |  | - |
| NM_152392 | AHSA2 | 1.50 |  | - |
| NM_006763 | BTG2 | 1.50 |  | BTG2 |
| NM_004099 | - | 1.50 |  | - |
| NM_175622 | MT1JP | 1.50 |  | - |
| NM_002770 | PRSS2 | 1.49 |  | - |
| NM_006266 | RALGDS | 1.49 |  | - |
| AK095888 | CD109 | 1.49 |  | - |
| NM_020639 | RIPK4 | 1.48 |  | - |
| AY008274 | - | 1.48 |  | - |
| U60266 | MAN2B1 | 1.48 |  | - |
| NM_005947 | MT1B | 1.47 |  | - |
| NM_005902 | SMAD3 | 1.47 |  | - |
| AL834364 | TMEM64 | 1.47 |  | - |
| NM_022842 | CDCP1 | 1.47 |  | - |
| AY369207 | - | 1.47 |  | - |
| NM_016496 | MARCH2 | 1.47 |  | - |
| NM_003897 | IER3 | 1.46 |  | - |
| AB006622 | - | 1.46 |  | - |
| NM_014798 | PLEKHM1 | 1.46 |  | - |
| AB043587 | ACBD3 | 1.46 |  | - |
| NM_031894 | FTHL17 | 1.46 |  | - |
| NM_001860 | SLC31A2 | 1.45 |  | - |
| NM_031858 | NBR1 | 1.45 |  | - |
| AJ002425 | HSAJ2425 | 1.45 |  | - |
| X97261 | MT1L | 1.45 |  | - |
| NM_183422 | TSC22D1 | 1.44 |  | - |
| NM_014734 | KIAA0247 | 1.44 |  | - |
| AK126845 | - | 1.44 |  | - |
| NM_000527 | LDLR | 1.43 |  | - |
| AB007877 | HSPA12A | 1.43 |  | - |
| NM_018975 | TERF2IP | 1.43 |  | - |
| NM_002899 | RBP1 | 1.43 |  | - |
| NM_005951 | MT1H | 1.42 |  | - |
| NM_024430 | PSTPIP2 | 1.42 |  | - |
| NM_001873 | CPE | 1.42 |  | - |
| NM_152718 | VWCE | 1.42 |  | - |
| NM_024599 | RHBDF2 | 1.42 |  | - |
| NM_133645 | - | 1.42 |  | - |
| NM_002245 | KCNK1 | 1.42 |  | - |
| NM_021724 | NR1D1 | 1.42 |  | - |
| NM_014933 | SEC31L1 | 1.41 |  | - |
| NM_024836 | ZNF672 | 1.41 |  | - |
| NM_203305 | FAM102A | 1.41 |  | - |
| AL833749 | LOC146439 | 1.41 |  | - |
| NM_016086 | STYXL1 | 1.40 |  | - |
| NM_014964 | EPN2 | 1.40 |  | - |
| AB067470 | - | 1.40 |  | - |
| NM_017818 | WDR8 | 1.40 |  | - |
| NM_198471 | ANKRD47 | 1.39 |  | - |
| NM_018426 | TMEM63B | 1.39 |  | - |
| NM_004487 | GOLGB1 | 1.39 |  | - |
| NM_203330 | CD59 | 1.38 |  | - |
| NM_004233 | CD83 | 1.38 |  | - |
| AK023379 | - | 1.38 |  | - |
| NM_020896 | OSBPL5 | 1.38 |  | - |
| NM_020248 | CTNNBIP1 | 1.38 |  | - |
| NM_001300 | KLF6 | 1.37 |  | - |
| NM_005990 | STK10 | 1.37 |  | - |
| NM_004156 | - | 1.37 |  | - |
| NM_002771 | PRSS3 | 1.36 |  | - |
| NM_032409 | PINK1 | 1.36 |  | - |
| NM_152264 | SLC39A13 | 1.36 |  | - |
| NM_002473 | MYH9 | 1.36 |  | - |
| NM_032797 | AMID | 1.36 |  | - |
| NM_005437 | NCOA4 | 1.36 |  | - |
| NM_017458 | MVP | 1.36 |  | - |
| NM_015946 | PELO | 1.36 |  | - |
| NM_021960 | MCL1 | 1.35 |  | - |
| NM_017983 | WIPI1 | 1.35 |  | - |
| NM_004199 | P4HA2 | 1.35 |  | - |
| NM_014256 | B3GNT3 | 1.34 |  | - |
| NM_017572 | MKNK2 | 1.34 |  | - |
| NM_148965 | - | 1.34 |  | - |
| NM_018428 | UTP6 | 1.34 |  | - |
| NM_004251 | RAB9A | 1.34 |  | - |
| NM_012103 | AUP1 | 1.33 |  | - |
| NM_022818 | MAP1LC3B | 1.33 |  | - |
| NM_001333 | CTSL2 | 1.32 |  | - |
| NM_005415 | SLC20A1 | 1.32 |  | - |
| NM_002872 | RAC2 | 1.32 |  | - |
| NM_144626 | TMEM125 | 1.32 |  | - |
| NM_006024 | TAX1BP1 | 1.32 |  | - |
| BC033256 | FAM83H | 1.32 |  | - |
| NM_133491 | SAT2 | 1.32 |  | - |
| BC047452 | - | 1.32 |  | - |
| NM_021199 | SQRDL | 1.31 |  | - |
| NM_015517 | MIZF | 1.31 |  | - |
| AY007122 | - | 1.31 |  | - |
| NM_031484 | MARVELD1 | 1.31 |  | - |
| NM_002859 | PXN | 1.31 |  | - |
| NM_002087 | GRN | 1.30 |  | - |
| NM_000181 | GUSB | 1.30 |  | - |
| AF289596 | - | 1.30 |  | - |
| NM_020412 | CHMP1B | 1.30 |  | - |
| NM_014313 | TMEM50A | 1.30 |  | - |
| NM_016203 | PRKAG2 | 1.30 |  | - |
| AB067491 | - | 1.30 |  | - |
| AK055659 | TSPAN5 | 1.30 |  | - |
| NM_015383 | NBPF14 | 1.30 |  | - |
| NM_018158 | SLC4A1AP | 1.29 |  | - |
| NM_024928 | OBFC1 | 1.29 |  | - |
| AL137347 | - | 1.29 |  | - |
| NM_006795 | EHD1 | 1.29 |  | - |
| NM_032305 | POLR3GL | 1.29 |  | - |
| NM_013445 | GAD1 | 1.29 |  | - |
| NM_002309 | LIF | 1.28 |  | LIF |
| AK027693 | - | 1.28 |  | - |
| NM_004747 | DLG5 | 1.28 |  | - |
| NM_003295 | TPT1 | 1.28 |  | - |
| K03200 | LOC440995 | 1.27 |  | - |
| NM_012142 | - | 1.27 |  | - |
| NM_015994 | ATP6V1D | 1.27 |  | - |
| NM_024061 | - | 1.27 |  | - |
| NM_001001433 | STX16 | 1.27 |  | - |
| NM_177939 | - | 1.27 |  | - |
| NM_003312 | TST | 1.26 |  | - |
| NM_032901 | C12orf62 | 1.26 |  | - |
| NM_012094 | PRDX5 | 1.24 |  | - |
| NM_015518 | - | 1.24 |  | - |
| BX647358 | - | 1.23 |  | - |
| NM_014872 | ZBTB5 | 1.23 |  | - |
| NM_138452 | DHRS1 | 1.22 |  | - |
| NM_012245 | SNW1 | 1.22 |  | - |
| NM_144505 | KLK8 | 1.22 |  | - |
| NM_001394 | DUSP4 | 1.22 |  | - |
| AK122734 | - | 1.20 |  | - |
| NM_031844 | HNRPU | -1.21 |  | - |
| NM_001416 | EIF4A1 | -1.22 |  | - |
| NM_017785 | CCDC99 | -1.22 |  | - |
| AF355801 | CYP3A5 | -1.23 |  | - |
| NM_018983 | NOLA1 | -1.23 |  | - |
| NM_001101 | ACTB | -1.23 |  | - |
| NM_172251 | MRPL54 | -1.24 |  | - |
| NM_021874 | CDC25B | -1.24 |  | - |
| NM_002788 | PSMA3 | -1.24 |  | - |
| NM_003146 | SSRP1 | -1.24 |  | - |
| NM_004541 | NDUFA1 | -1.24 |  | - |
| NM_024789 | C10orf77 | -1.24 |  | - |
| M26004 | CR2 | -1.25 |  | - |
| NM_015973 | GAL | -1.25 |  | - |
| NM_001001790 | FBXO10 | -1.25 |  | - |
| NM_002826 | QSCN6 | -1.26 |  | - |
| NM_022371 | TOR3A | -1.27 |  | - |
| NM_173714 | - | -1.27 |  | - |
| NM_014214 | IMPA2 | -1.27 |  | - |
| BQ304306 | - | -1.27 |  | - |
| NM_178014 | TUBB | -1.27 |  | - |
| NM_016059 | PPIL1 | -1.27 |  | - |
| NM_005175 | ATP5G1 | -1.28 |  | - |
| NM_005274 | GNG5 | -1.28 |  | - |
| NM_012124 | CHORDC1 | -1.28 |  | - |
| NM_003056 | SLC19A1 | -1.28 |  | - |
| NM_138730 | HMGN3 | -1.28 |  | - |
| NM_032356 | LSMD1 | -1.28 |  | - |
| NM_145212 | MRPL30 | -1.29 |  | - |
| NM_203304 | RKHD1 | -1.29 |  | - |
| NM_004548 | NDUFB10 | -1.29 |  | - |
| NM_182903 | KIF9 | -1.30 |  | - |
| NM_031426 | - | -1.30 |  | - |
| NM_022085 | TXNDC5 | -1.30 |  | - |
| NM_002882 | RANBP1 | -1.30 |  | - |
| NM_006243 | PPP2R5A | -1.30 |  | - |
| NM_017899 | TESC | -1.30 |  | - |
| NM_003981 | PRC1 | -1.31 |  | PRC1 |
| NM_080649 | APEX1 | -1.31 |  | - |
| NM_006938 | SNRPD1 | -1.31 |  | - |
| NM_012254 | SLC27A5 | -1.31 |  | - |
| NM_001536 | - | -1.31 |  | - |
| NM_016183 | C1orf33 | -1.31 |  | - |
| NM_000304 | PMP22 | -1.32 |  | - |
| NM_018235 | CNDP2 | -1.32 |  | - |
| NM_016202 | ZNF580 | -1.32 |  | - |
| NM_003720 | DSCR2 | -1.33 |  | - |
| NM_002528 | NTHL1 | -1.33 |  | - |
| NM_018719 | CDCA7L | -1.33 |  | - |
| NM_016424 | CROP | -1.33 |  | - |
| NM_002731 | PRKACB | -1.33 |  | - |
| NM_005782 | THOC4 | -1.33 |  | - |
| NM_000214 | JAG1 | -1.33 |  | - |
| NM_006848 | CCDC85B | -1.34 |  | - |
| NM_152274 | FAM58A | -1.34 |  | - |
| NM_004450 | ERH | -1.34 |  | - |
| NM_006392 | NOL5A | -1.34 |  | - |
| NM_002539 | ODC1 | -1.35 |  | - |
| NM_138699 | LOC93622 | -1.35 |  | - |
| NM_152834 | TMEM18 | -1.35 |  | - |
| NM_004219 | PTTG1 | -1.35 |  | PTTG1 |
| NM_023936 | MRPS34 | -1.35 |  | - |
| NM_019013 | FAM64A | -1.36 |  | - |
| NM_024658 | IPO4 | -1.36 |  | - |
| AK095849 | - | -1.36 |  | - |
| NM_032549 | IMMP2L | -1.36 |  | - |
| NM_005566 | LDHA | -1.36 |  | - |
| NM_170711 | DAZAP1 | -1.36 |  | - |
| D28589 | - | -1.37 |  | - |
| NM_020062 | SLC2A4RG | -1.37 |  | - |
| NM_005371 | METTL1 | -1.37 |  | - |
| NM_178868 | CMTM8 | -1.37 |  | - |
| BC000712 | - | -1.37 |  | - |
| NM_005176 | - | -1.38 |  | - |
| NM_018048 | FLJ10292 | -1.38 |  | - |
| NM_015604 | - | -1.38 |  | - |
| NM_001212 | C1QBP | -1.38 |  | - |
| NM_144998 | STRA13 | -1.38 |  | - |
| AK074122 | - | -1.38 |  | - |
| AK130101 | - | -1.39 |  | - |
| NM_003095 | SNRPF | -1.39 |  | - |
| NM_013293 | TRA2A | -1.39 |  | - |
| NM_002949 | MRPL12 | -1.40 |  | - |
| NM_001933 | DLST | -1.40 |  | - |
| NM_019610 | RBMXL1 | -1.40 |  | - |
| NM_002498 | NEK3 | -1.40 |  | - |
| NM_144999 | LRRC45 | -1.41 |  | - |
| NM_000392 | ABCC2 | -1.41 |  | - |
| BC009510 | - | -1.42 |  | - |
| NM_002139 | RBMX | -1.42 |  | - |
| NM_025259 | - | -1.43 |  | - |
| NM_021992 | TMSL8 | -1.43 |  | TMSL8 |
| NM_006452 | PAICS | -1.43 |  | - |
| NM_024834 | C10orf119 | -1.45 |  | - |
| NM_032457 | PCDH7 | -1.45 |  | - |
| NM_199342 | CCDC23 | -1.45 |  | - |
| XM_374273 | LOC389669 | -1.46 |  | - |
| BC071732 | PTMA | -1.46 |  | - |
| NM_170746 | C11orf31 | -1.46 |  | - |
| NM_002388 | MCM3 | -1.46 |  | - |
| NM_032730 | RTN4IP1 | -1.47 |  | - |
| NM_002358 | MAD2L1 | -1.47 |  | - |
| NM_001444 | FABP5 | -1.48 |  | - |
| NM_020810 | TRMT5 | -1.49 |  | - |
| NM_014316 | CARHSP1 | -1.49 |  | - |
| NM_024060 | AHNAK | -1.50 |  | - |
| NM_213723 | - | -1.50 |  | - |
| BC045739 | - | -1.50 |  | - |
| NM_002894 | RBBP8 | -1.51 |  | - |
| NM_020789 | IGSF9 | -1.53 |  | - |
| NM_001009936 | PHF19 | -1.54 |  | - |
| AF152351 | - | -1.54 |  | - |
| NM_002497 | NEK2 | -1.54 |  | - |
| BC044933 | - | -1.54 |  | - |
| NM_031217 | KIF18A | -1.56 |  | - |
| NM_001875 | CPS1 | -1.57 |  | - |
| NM_032359 | C3orf26 | -1.57 |  | - |
| NM_002105 | H2AFX | -1.58 |  | - |
| BX537987 | TRIM59 | -1.58 |  | - |
| NM_145231 | C14orf143 | -1.59 |  | - |
| NM_014501 | UBE2S | -1.60 |  | - |
| NM_016095 | GINS2 | -1.60 |  | - |
| NM_152524 | SGOL2 | -1.61 |  | - |
| NM_017779 | DEPDC1 | -1.61 |  | - |
| NM_014042 | C11orf51 | -1.65 |  | - |
| NM_002157 | HSPE1 | -1.65 |  | - |
| NM_006751 | SSFA2 | -1.65 |  | - |
| NM_001809 | CENPA | -1.69 |  | - |
| NM_003385 | VSNL1 | -1.81 |  | - |
| NM_003380 | VIM | -1.82 |  | - |
| NM_145244 | DDIT4L | -1.85 |  | - |
| NM_032438 | L3MBTL3 | -2.03 |  | - |
| NM_005573 | LMNB1 | -2.28 |  | - |
| NM_025049 | C15orf20 | -2.61 |  | - |
