# Supplementary material for: Inhibition of Chk1 Kills Tetraploid Tumor Cells through a p53-Dependent Pathway
Source: PLoS One. 2007 Dec 26;2(12):e1337. doi: 10.1371/journal.pone.0001337 (PMC2131784; doi:10.1371/journal.pone.0001337)
Supplement: Table S2 — (0.07 MB DOC) [file pone.0001337.s002.doc]

| **siRNA** | **Sense sequenc** | **Reference** |
| --- | --- | --- |
| ATF3a | 5’-GAGGCGACGAGAAAGAAAUTT-3’ |  |
| ATF3b | 5’-GAAGAAGGAGAAGACGGAGTT-3’ | Janz et al. (2006), Blood, 107, 2536-2539 |
| Atg12 | 5’-CAGAGGAACCUGCUGGCGATT-3’ | Boya et al. (2005), Mol Cell Biol, 25, 1025-1040 |
| AurB | 5’-GGAGGAGGAUCUACUUGAUTT-3’ |  |
| Bad a | 5’-AAGAAGGGACTTCCTCGCCCGTT-3’ | Jin et al. (2004), J Biol Chem, 279, 23837-23844. |
| Bad b | 5’-CUGGGCAGCCAUCUUGAAUTT-3’ |  |
| Bax | 5’-GGUGCCGGAACUGAUCAGATT-3’ |  |
| Bcl-2 | 5’-GCUGCACCUGACGCCCUUCTT-3’ | Maley et al. (2004), Cancer Res, 64, 7629-7633. |
| Bcl-X_L_ | 5’-CAGGGACAGCAUAUCAGAGTT-3’ | Jiang and Milner (2003), Genes Dev, 17, 832-837 |
| Bid | 5’-GAAGACAUCAUCCGGAAUATT-3’ | Wagner et al. (2004), J Biol Chem, 279, 35047-35052 |
| Bub1 | 5’-AUACCACAAUGACCCAAGATT-3’ | Johnson et al. (2004), J Cell Sci, 117, 1577-1589 |
| BubR1 | 5'-AAGGGAAGCCGAGCUGUUGAC-3’ | Wang et al. (2004), Blood, 103, 1278-1285. |
| Chk1a | 5’-GCGUGCCGUAGACUGUCCATT-3’ |  |
| Chk1b | 5’-UCGUGAGCGUUUGUUGAACTT-3’ | Pichierri and Rosselli (2004), Embo J, 23, 1178-1187 |
| Chk2a | 5’-UGUGUGAAUGACAACUACUTT-3’ |  |
| Chk2b | 5’-AUUGCACUGUCACUAAGCATT-3’ |  |
| Mad2 | 5’-ACCUUUACUCGAGUGCAGATT3’ | Nitta et al. (2004), Oncogene, 23, 6548-6558 |
| p53a | 5’-GACUCCAGUGGUAAUCUACTT-3’ |  |
| p53b | 5’-GUGAGCGCUUCGAGAUGUUTT-3’ | Gu et al. (2004), Oncogene, 23, 1300-1307 |
| p53BP1 | 5’-GAACGAGGAGACGGUAATATT-3’ | DiTullio et al. (2002), Nat Cell Biol, 4, 998-1002 |
| PUMAa | 5’-UCUCAUCAUGGGACUCCUGTT-3’ |  |
| PUMAb | 5’-UUGAGGUCGUCCGCCAUCCTT-3’ |  |
| SSATa | 5’-GGUGCUCUCCAAAACCAUCTT-3’ |  |
| SSATb | 5’-GGACACAGCAUUGUUGGUUTT-3’ | Chen et al. (2003), Mol Pharmacol, 64, 1153-1159 |
| Survivin a | 5’-GCGCCUGCACCCCGGAGCGTT-3’ |  |
| Survivin b | 5’-GGCUGGCUUCAUCCACUGCTT-3’ | Ling and Li (2004). Biotechniques, 36, 450-454, 456-460 |
| VDAC1 | 5’-GUACGGCCUGACGUUUACATT-3’ |  |
| Villin 3a | 5’-AAGGAAUCCUUAGCGAUGAGATT-3’ |  |
| Villin 3b | 5’-CCCCAAAGAUUGGCUUUCCTT-3’ | Wan et al. (2005), Cancer Res, 65, 2406-2411 |

**Table S2: siRNA sequences**
