# Supplementary figures and images for: Inhibition of Chk1 Kills Tetraploid Tumor Cells through a p53-Dependent Pathway
Source: PLoS One. 2007 Dec 26;2(12):e1337. doi: 10.1371/journal.pone.0001337 (PMC2131784; doi:10.1371/journal.pone.0001337)

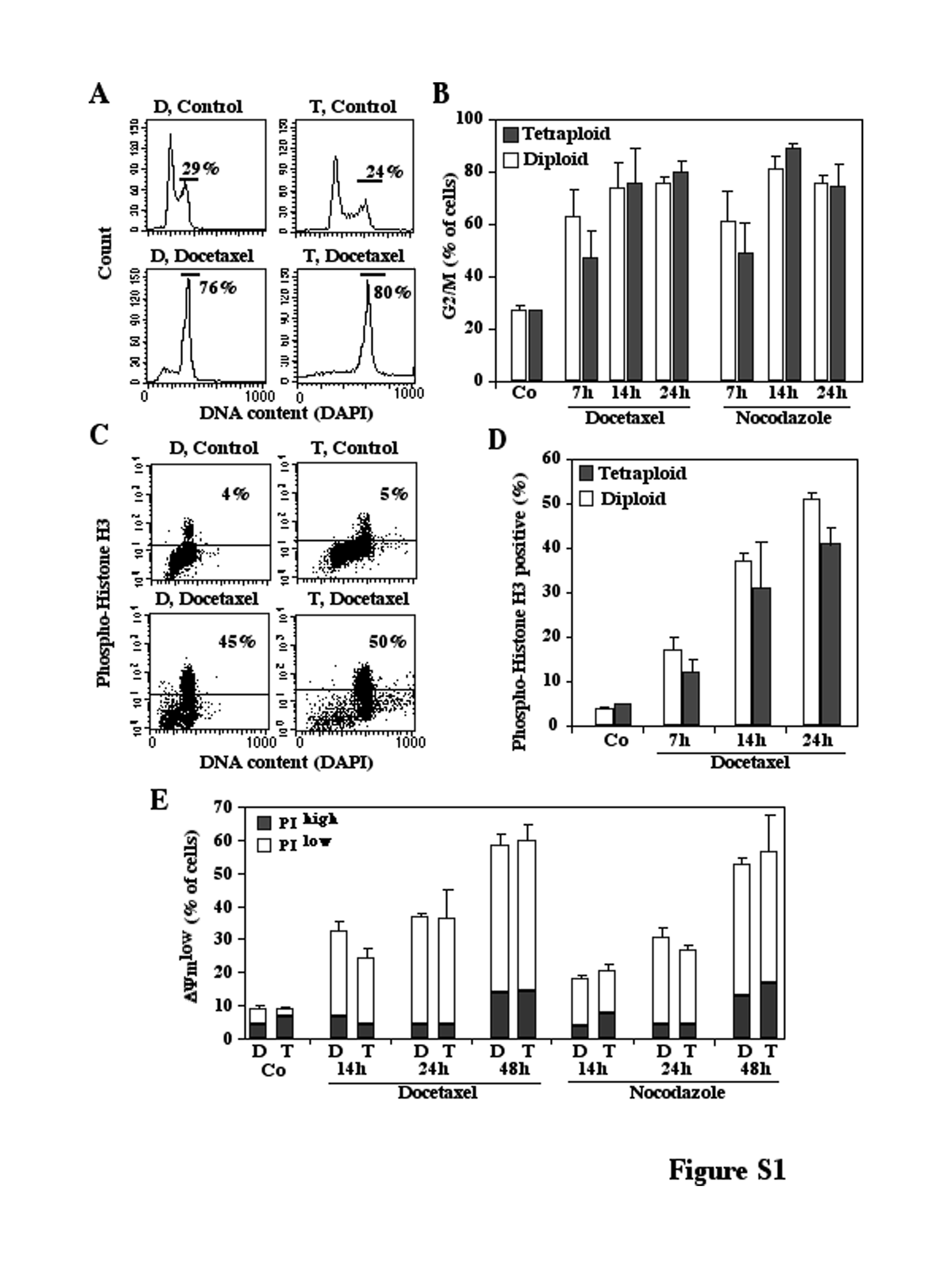

Supplement: Figure S1 — Intact SAC in tetraploid HCT116 cells. Diploid (D) or tetraploid (T) HCT116 cells were left untreated or cultured in the presence of docetaxel (1 µM) or nocodazole (1 µM) for the indicated period (24 h in A and C) and subjected to cell cycle analyses (A, B) by labeling with 4,6-diamidino-2-phenylindole (DAPI) or stained for the detection of phospho-histone H3, a histone that is specifically phosphorylated during mitosis (C, D). Representative FACS data are shown in A and C and quantitative data (X±SEM, n = 3) are shown in B and D. In addition the toxicity of docetaxel and nocodazole was determined by staining with DiOC6(3)/PI, yielding information on the frequency of dying (DiOC6(3)low PI-) or dead (DiOC6(3)low PI+) cells (E) (2.88 MB TIF) [file pone.0001337.s003.tif]

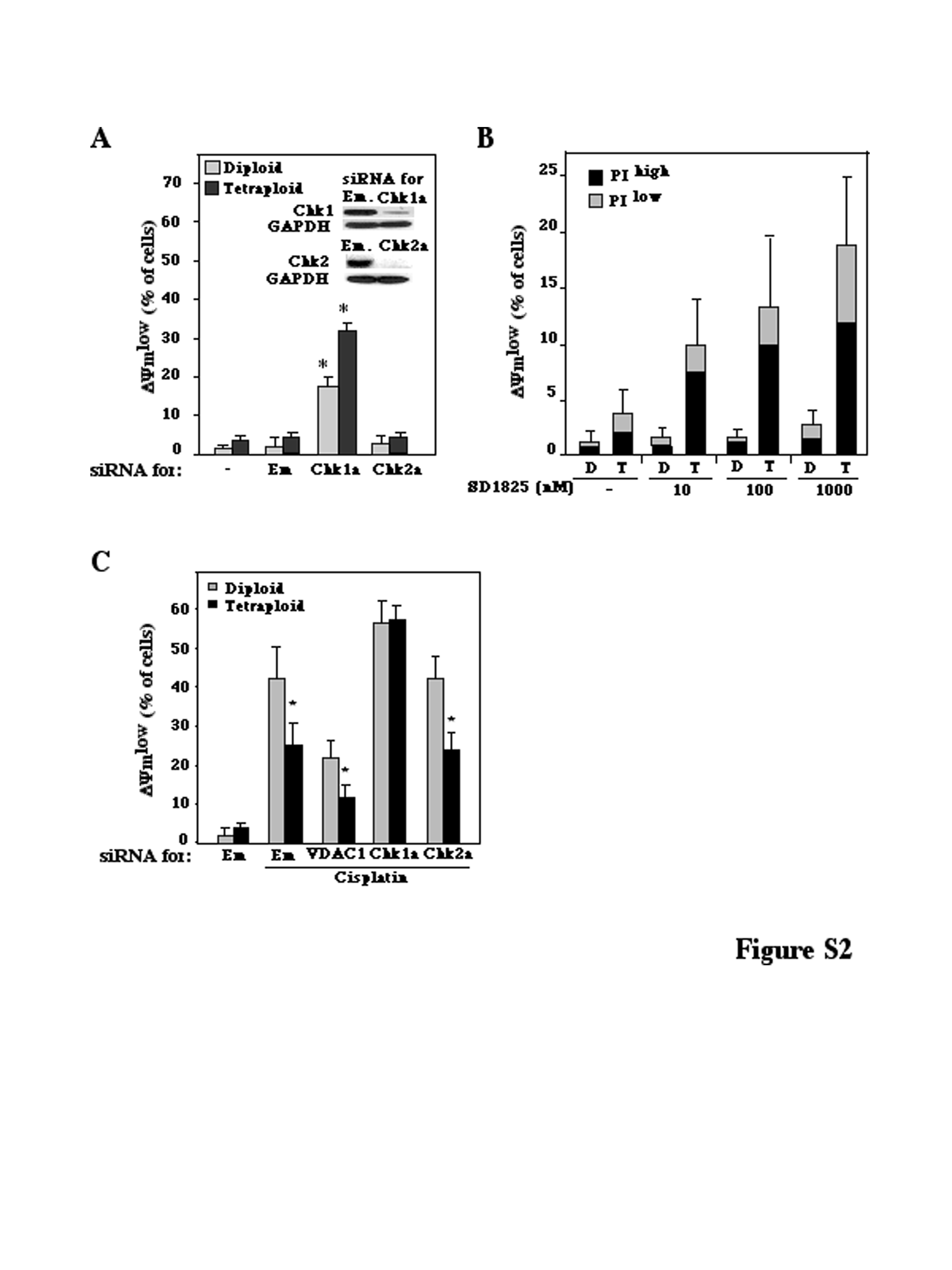

Supplement: Figure S2 — Apoptosis induction by depletion or inhibition of Chk1 in tetraploid RKO cells. A. Chk1 depletion kills tetraploid RKO colon cancer cells. RKO cells were transfected with siRNAs that deplete Chk1 (Chk1a) or Chk2 (Chk2a), as demonstrated by the immunoblot performed 48 h after transfection. The frequency of dying (ΔΨmlow) cells was determined by staining with tetramethyl rhodamine methylester (TMRM, 150 nM, 15 min). Asterisks mark significant (p<0.01) effects of Chk1 depletion. B. Chk1 inhibition by SD1825 kills tetraploid RKO cells. RKO cells were cultured with the indicated doses of SD1825, and the frequency of dead and dying cells was measured by simultaneous staining with DiOC6(3) and PI (as in Fig. 3A) 48 h later. C. Combined effects of Chk1 inhibition and cisplatin (20 µM) on tetraploid RKO cells. Diploid or tetraploid RKO cells were treated by siRNAs targeting emerin (as a negative control), VDAC1 (as a positive control of apoptosis inhibition), Chk1 or Chk2, followed by staining with TMRM to measure ΔΨm dissipation (1.49 MB TIF) [file pone.0001337.s004.tif]

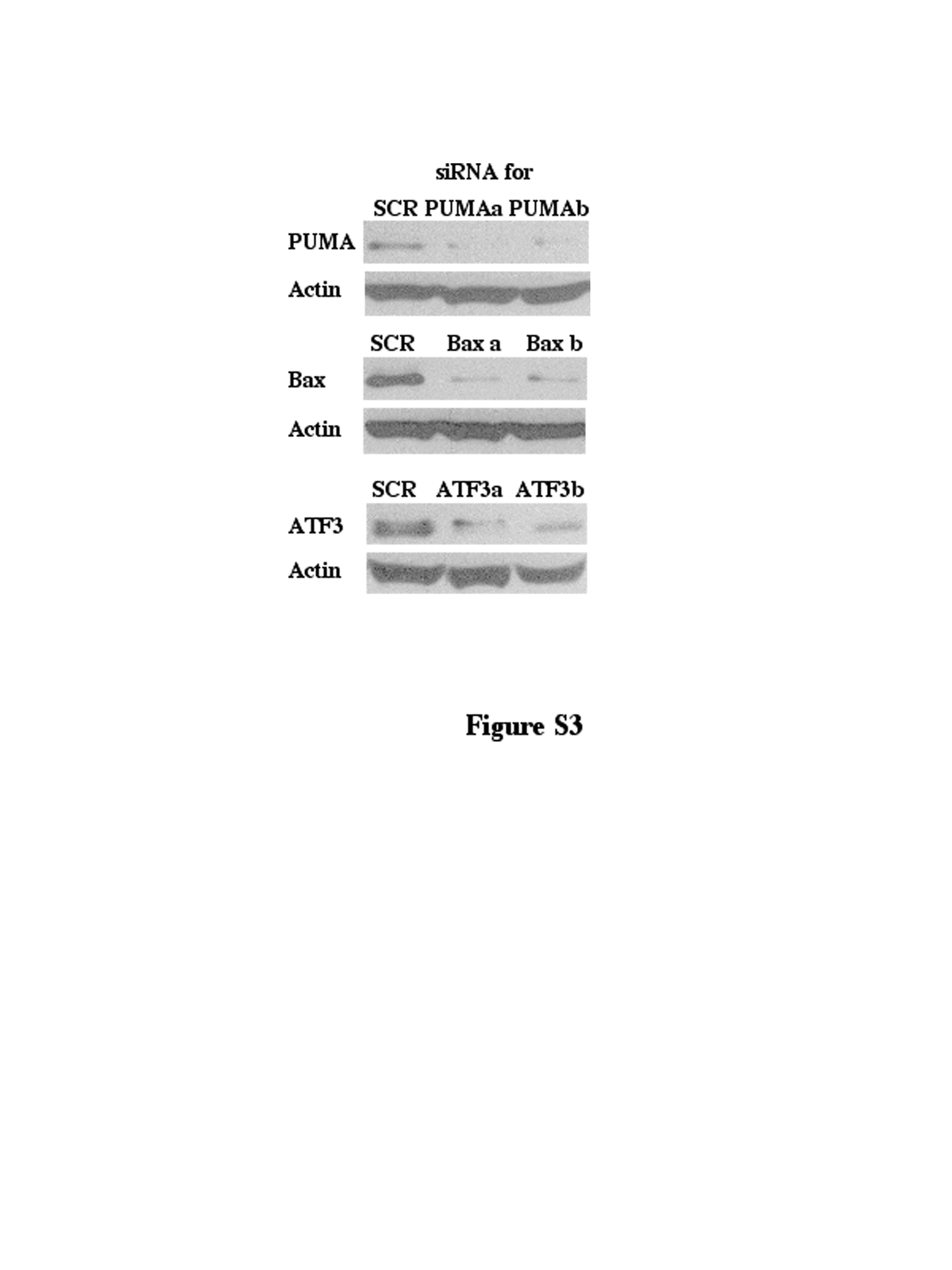

Supplement: Figure S3 — The efficacy of the siRNAs specific for Puma, ATF3 and Bax was determined by immunoblot, 48 hours after transfection of tetraploid HCT116 cells with control scrambled (SCR) siRNA or the indicated specific siRNAs. The polyclonal rabbit antibodies specific for Puma, ATF3 and Bax were from Ψ ProSci Incorporated, Santa Cruz Biotechnology, and Upstate Biotechnology, respectively. Equal loading was determined with anti-actin antibody (monoclonal mouse IgG1 from AbCys) (3.69 MB TIF) [file pone.0001337.s005.tif]

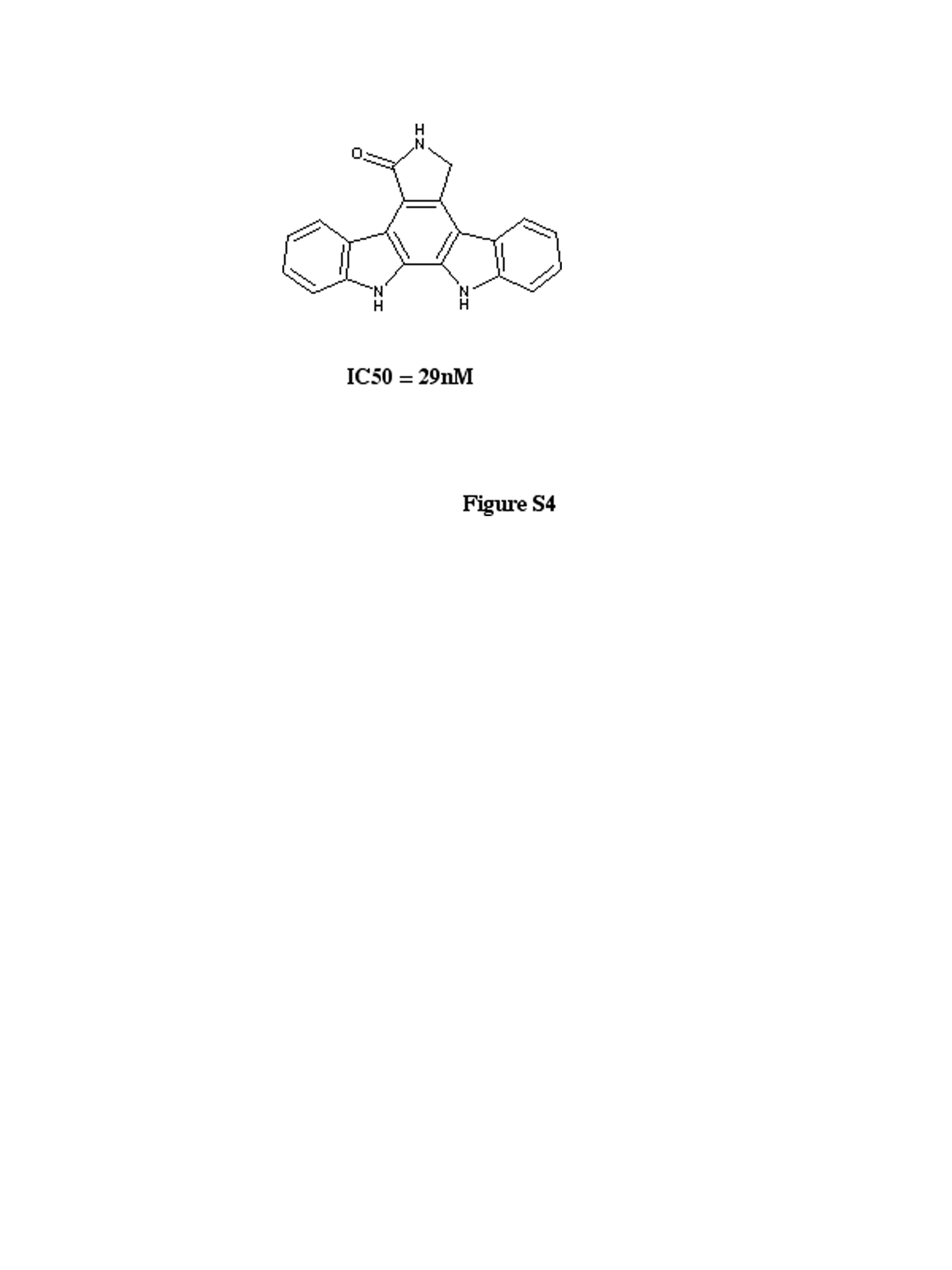

Supplement: Figure S4 — Chemical structure of SD 1825 and its IC50 for recombinant Chk1 (0.96 MB TIF) [file pone.0001337.s006.tif]
